# Verbalizing phylogenomic conflict: Representation of node congruence across competing reconstructions of the neoavian explosion

**DOI:** 10.1371/journal.pcbi.1006493

**Published:** 2019-02-15

**Authors:** Nico M. Franz, Lukas J. Musher, Joseph W. Brown, Shizhuo Yu, Bertram Ludäscher

**Affiliations:** 1 School of Life Sciences, Arizona State University, Tempe, Arizona, United States of America; 2 Richard Gilder Graduate School and Department of Ornithology, American Museum of Natural History, New York, New York, United States of America; 3 Department of Animal and Plant Sciences, University of Sheffield, Sheffield, United Kingdom; 4 Department of Computer Science, University of California at Davis, Davis, California, United States of America; 5 School of Information Sciences, University of Illinois at Urbana-Champaign, Champaign, Illinois, United States of America; Temple University, UNITED STATES

## Abstract

Phylogenomic research is accelerating the publication of landmark studies that aim to resolve deep divergences of major organismal groups. Meanwhile, systems for identifying and integrating the products of phylogenomic inference–such as newly supported clade concepts–have not kept pace. However, the ability to *verbalize* node concept congruence and conflict across multiple, in effect simultaneously endorsed phylogenomic hypotheses, is a prerequisite for building synthetic data environments for biological systematics and other domains impacted by these conflicting inferences. Here we develop a novel solution to the conflict verbalization challenge, based on a logic representation and reasoning approach that utilizes the language of Region Connection Calculus (RCC–5) to produce consistent *alignments* of node concepts endorsed by incongruent phylogenomic studies. The approach employs clade concept labels to individuate concepts used by each source, even if these carry identical names. Indirect RCC–5 modeling of *intensional* (property-based) node concept definitions, facilitated by the local relaxation of coverage constraints, allows parent concepts to attain congruence in spite of their differentially sampled children. To demonstrate the feasibility of this approach, we align two recent phylogenomic reconstructions of higher-level avian groups that entail strong conflict in the "neoavian explosion" region. According to our representations, this conflict is constituted by 26 instances of input "whole concept" overlap. These instances are further resolvable in the output labeling schemes and visualizations as "split concepts", which provide the labels and relations needed to build truly synthetic phylogenomic data environments. Because the RCC–5 alignments fundamentally reflect the trained, logic-enabled judgments of systematic experts, future designs for such environments need to promote a culture where experts routinely assess the intensionalities of node concepts published by our peers–even and especially when we are not in agreement with each other.

## Author summary

Synthetic platforms for phylogenomic knowledge tend to manage conflict between different evolutionary reconstructions in the following way: "If we do not agree, then it is either our view over yours, or we just collapse all conflicting node concepts into polytomies". We argue that this is not an equitable way to realize synthesis in this domain. For instance, it would not be an adequate solution for building a unified data environment where authors can endorse and yet also reconcile their diverging perspectives, side by side. Hence, we develop a novel system for *verbalizing*–i.e., consistently identifying and aligning–incongruent node concepts that reflects a more forward-looking attitude: "We may not agree with you, but nevertheless we *understand* your phylogenomic inference well enough to *express* our disagreements in a logic-compatible syntax. We can therefore maximize the translatability of data linked to our diverging phylogenomic hypotheses". We show that achieving phylogenomic synthesis fundamentally depends on the application of trained expert judgment to assert parent node congruence in spite of incongruently sampled children.

## Introduction

Three years ago, Jarvis et al. (2014; henceforth 2014.JEA) [1] published a landmark reconstruction of higher-level bird relationships. Within 12 months, however, another analysis by Prum et al. (2015; henceforth 2015.PEA) [2] failed to support several of the deep divergences recovered in the preceding study, particularly within the Neoaves sec. (*secundum* = according to) Sibley et al. (1988) [3]. Thomas (2015) [4] used the term "neoavian explosion" to characterize the lack of congruence between inferences of early-diverging lineages (see also [5]). Similarly, after reviewing six phylogenomic studies, Suh [6] concluded that the root region of the Neoaves constitutes a "hard polytomy". Multiple analyses have dissected the impact of differential biases in terminal and genome sampling, as well as evolutionary modeling and analysis constraints, on resolving this complex radiation [7, 8, 9]. Suh [6] argues that a well resolved consensus is not imminent (though see [10]). Brown et al. (2017) [11] analyzed nearly 300 avian phylogenies, finding that the most recent studies "continue to contribute new edges".

These recent advancements provide an opportunity to reflect on how synthesis should be realized in the age of phylogenomics [11, 12, 13]. The neoavian explosion can be considered a use case where multiple studies provide strong signals for conflicting hierarchies. Resolution towards a single, universally adopted tree is unlikely in the short term.

Rather than focusing on the analytical challenges along the path towards unitary resolution [9], we turn to the issue of how the persistence of conflict affects the design of synthetic data infrastructures. In other words, how do we build a data service for phylogenomic knowledge in the face of persistent conflict? This question is of broad relevance to systematists, comparative evolutionary biologists, and designers of biological information services interested in robust, reproducible, and reusable phylogenomic data. And it turns on the issue of improving identifiers and identifier-to-identifier relationships for this domain.

Particularly *verbal* representations of the neoavian explosion are not well designed for conflict representation and synthesis [14]. To alleviate this, some authors use tree alignment graphs in combination with color and width variations to identify regions (edges) of phylogenomic congruence and conflict [15]. Other authors may show multiple incongruent trees side-by-side, using color schemes for congruent clade sections [9]. Yet others may use tanglegrams that are enhanced to highlight congruence [4], rooted galled networks [16] or neighbor-net visualizations [17] that show split networks for conflicting topology regions, or simply provide a consensus tree in which incongruent bifurcating branch inferences are collapsed into polytomy [6].

Verbalizing phylogenomic congruence and conflict in open, synthetic knowledge environments [13] constitutes a novel challenge for which traditional naming solutions in systematics are inadequate. The aforementioned studies implicitly support this claim. All use overlapping sets of Code-compliant [18] and other higher-level names in the Linnaean tradition, with sources including [19] or [20]. To identify these source-specific name usages, we will utilize the *taxonomic concept label* convention of [14]. Accordingly, name usages sec. 2014.JEA are prefixed with "2014.", whereas name usages sec. 2015.PEA are prefixed with "2015."

We diagnose the verbalization challenge as follows. (1) In some instances, identical clade names are polysemic–i.e., have multiple meanings–across studies. For instance, 2015.Pelecaniformes excludes 2015.Phalacrocoracidae, yet 2014.Pelecaniformes includes 2014.Phalacrocoracidae; reflecting on two incongruent meanings of "Pelecaniformes". (2) In other cases, two or more non-identical names have congruent meanings, e.g., 2015.Strisores and 2014.Caprimulgimorphae. (3) Names that are unique to just one study–e.g., 2015.Aequorlitornithes or 2014.Cursorimorphae–are not always reconcilable in meaning without additional human effort, thereby adding an element of referential uncertainty to the apparent conflict. (4) Lastly, many of the newly inferred and conflicting edges are not named at all. There is an implicit preference for labeling edges when suitable names are already available. However, unnamed edges can create situations where conflict cannot be verbalized and reconciled in a data environment, due to the lack of syntactic structure ("names").

Jointly, the effects of polysemic names, synonymous names, exclusive yet hard-to-reconcile names, and conflicting unnamed edges are symptomatic of an information culture that is not ready for the identifier and identifier-to-identifier relationship challenges inherent in representing phylogenomic conflict. Suppose we wish to build a collaborative knowledge environment towards inferring "the tree of life" (though see [12]). The design should allow us to individually represent and at the same integrate conflicting hierarchies, from the tips to the root. The system should respond to name-based data queries across these hierarchies, and return whether they are congruent or how they conflict in meaning. Clearly, the name usages of each individual source are not suited for this integration task. Traditional, Linnaean conventions allow for names to have evolving phylogenomic meanings across hierarchies and are therefore too under-powered for our purpose [21].

At root, this is a novel *conceptual* challenge for systematics and comparative evolutionary biology, made imperative by the accelerated generation and ingestion of phylogenomic trees into open, dynamic knowledge bases for reliable integration and re-use [11, 13, 22, 23, 24]. The services that such environments aspire to provide require an appropriate theory of *node identity*, and hence a conception of multi-node congruence or incongruence across individual trees and entire synthesis versions.

Here we propose a solution to the phylogenomic conflict representation challenge. This solution requires collaboration between systematic experts, platform designers, and users of phylogenomic information. It is an extension of prior "concept taxonomy" research [14, 25, 26], and deploys logic reasoning to align tree hierarchies based on Region Connection Calculus (RCC–5) assertions of node congruence [27, 28, 29]. We demonstrate the feasibility of this approach by aligning subregions and entire phylogenomic trees inferred by 2015.PEA and 2014.JEA. In doing so, we address key representation challenges; such as the paraphyly of classification schemes used to label tree regions, and the inference of higher-level node congruence in spite of differentially sampled terminals. The alignment products for this use case constitute a novel answer to our central question: "how to build a synthetic knowledge environment in the face of persistent phylogenomic conflict?" The discussion focuses on the feasibility and desirability of creating such an integration service, emphasizing the role of trained expert judgment in providing them [30].

## Methods

### Syntactic and semantic conventions

**1. Taxa are models, concepts are mimics.** We typically refrain from using the terms "taxon", "taxa", or "clade(s)". We take taxa to constitute evolutionary, causally sustained entities whose members are manifested in the natural realm. The task for systematics is to successively approximate the identities and limits of these entities. Thus, we assign the status of 'models' to taxa, which systematists aim to 'mimic' through empirical theory making. This perspective allows for realism about taxa, and also for the possibility to let our representations *stand for* taxa [31], at any given time and however imperfectly, to support evolutionary inferences.

In reserving a model status for taxa, we can create a separate design space for the human theory- and language-making domain. In the latter, we speak only of taxonomic or phylogenomic *concepts–*the products of inference making [21].

**2. Sameness is limited to the same source.** Therefore, for the purpose of aligning the neoavian explosion use case, we need not speak of the "same taxa" or "same clades" at all. Similarly, we need not judge whether one reconstruction or the other more closely aligns with deep-branching avian taxa, i.e., which is (more) 'right'? Instead, our alignment is only concerned with modeling congruence and conflict across two sets of *concept* hierarchies. The concepts are *labeled* with the "sec." convention to maintain a one-to-one modeling relationship between concept labels and concepts (clade identity theories). Accordingly, there is also no need to say that, in recognizing each a concept with the taxonomic name Neornithes, the two author teams are authoring "the same concept". Instead, we model the two labels 2015.Neornithes and 2014.Neornithes, each of which symbolizes an individually generated phylogenomic theory region. As an outcome of our alignment, we may say that these two concepts are *congruent*, or not, reflecting the intensional alignment (to be specified below) of two phylogenomic theories. But, by virtue of their differential sources (authorship provenance), the two concepts 2015.Neornithes and 2014.Neornithes are never "the same". "Sameness" is limited in our approach to concepts whose labels contain an identical taxonomic name *and* which originate from a single phylogenomic hierarchy and source. That is, 2015.Neornithes and 2015.Neornithes are (labels for) the same concept.

### Knowledge representation and reasoning

The methods used herein are consistent with [14, 26, 32]. They utilize three core conventions: (1) taxonomic concept labels to identify concepts; (2) *is_a* relationships to assemble single-source hierarchies via parent/child relationships; and (3) RCC–5 articulations to express the relative congruence of concept regions across multi-sourced hierarchies. The RCC–5 articulation vocabulary entails (with corresponding symbol): congruence (= =), proper inclusion (>), inverse proper inclusion (<), overlap (><), and exclusion (!). *Disjunctions* of these articulations are a means to express uncertainty; as in: 2015.Neornithes {= = *or* > *or* <} 2014.Neornithes. All possible disjunctions generate a lattice of 32 relationships (R_32_), where the "base five" are the most logically constraining subset [33].

The alignments are generated with the open source Euler/X software toolkit [28]. The toolkit ingests multiple trees (T_1_, T_2_, T_3_, etc.) and articulation sets (A_1–2_, A_2–3_, etc.), converting them into a set of logic constraints. Together with other default or facultative constraints (C) needed for modeling tree hierarchies, these input constraints are then submitted to a logic reasoner that provide two main services. First, the reasoner infers whether all input constraints are jointly logically consistent, i.e., whether they permit at least one "possible world". Second, if consistency is attained, the reasoner infers the set of *Maximally Informative Relations* (MIR). The MIR constitute that unique set of RCC–5 articulations for every possible concept pair across the input sources from which the truth or falseness of any relationship in the R_32_ lattice can be deduced [14, 26, 33]. Many toolkit options and functions are designed to encode variable alignment input and output conditions, and to interactively obtain adequately constrained alignments. The toolkit also features a stylesheet-driven alignment input/output visualization service that utilizes directed acyclical graphs [28]. A step-wise account of the user/toolkit workflow interaction is provided in [26].

### Special challenges for multi-phylogeny alignments

Aligning phylogenomic trees entails several special representation and reasoning challenges. We address three aspects here that have not been dealt with extensively in previous publications.

**1. Representing intensional parent concept congruence via locally relaxed coverage.** The first challenge relates directly to the issue of parent node identity. Unlike comprehensive classifications or revisions [14, 26, 34], phylogenomic reconstructions typically do *not* aspire to sample low-level entities exhaustively. Instead, select exemplars are sampled among all possible low-level entities. The aim is to represent lower-lever diversity sufficiently well to infer reliable higher-level relationships. Often, terminal sampling is not only incomplete for any single reconstruction, but purposefully complementary to that of other analyses. Generating informative genome-level data remains resource-intensive [10]. This makes it prudent to coordinate terminal sampling globally, by prioritizing the reduction of gaps over redundant terminal sampling. In the case of 2015.PEA (198 terminals) versus 2014.JEA (48 terminals), only 12 species-level concept pairs have labels with identical taxonomic names.

By default, the logic toolkit applies a *coverage constraint* to every input concept region. Coverage means that the region of a parent is strictly circumscribed by the union of its children [35]. However, this constraint is relaxable, either globally for all concepts, or locally for select concepts. To relax coverage *locally*, the prefix "nc_" (no coverage) is used in the input, as in 2014.nc_Psittacidae. This means: *either* a parent concept's referential extension is circumscribed by the union of its explicitly included children, *or* there is a possibility of additional children being subsumed under that parent *but not mentioned* in the source phylogeny. Either scenario can yield consistent alignments. In other words, if a parent concept has relaxed coverage, it can attain congruence with another parent concept in spite of each parent having incongruent sets of child concepts.

Managing coverage in the toolkit input is not trivial. Relaxing coverage globally is akin to saying "anything goes", i.e., any parent could potentially include any child. This would yield innumerable possible worlds, and therefore has no value for our purpose. On the other hand, applying coverage globally means–counter-intuitively in the case of phylogenomic trees–that only parents with completely congruent sets of children can themselves attain congruence. The challenge for experts providing the input is thus to relax coverage *locally*, and strictly in the service of 'neutralizing' lower-level sampling differences between trees that should not yield conflict at higher levels.

**Fig 1:**
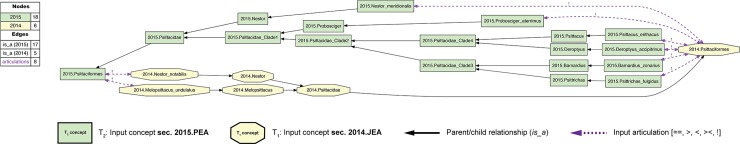
Input visualization for the 2015./2014.Psittaciformes alignment, with coverage globally applied. In all toolkit visualizations, the input and aligned, non-congruent concepts sec. 2015.PEA are shown as green rectangles (T_2_−18 concepts). Input and aligned, non-congruent concepts sec. 2014.JEA are shown as yellow octagons (T_1_−6 concepts). Congruent sets of aligned, multi-sourced concepts (first shown in Fig 4) are rendered in gray rectangles with rounded corners. In this input visualization, each phylogenomic tree is separately assembled via parent/child (*is_a*) relationships (solid black arrows). All species-level concepts sec. 2015.PEA and 2014.JEA are exclusive of each other. Under strict application of the coverage constraint, this is represented by asserting eight articulations (dashed magenta arrows) of disjointness (!) of each species-level concept from the other-sourced order-level concept. The legend indicates the numbers of nodes and edges for each input tree, parent/child relationships, and expert-asserted input articulations. See also S1 File.

**Fig 2:**
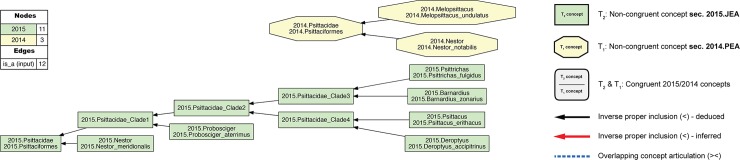
Alignment visualization for the 2015./2014.Psittaciformes alignment, with coverage globally applied. This alignment corresponds to the Fig 1 input, and shows reasoner-inferred non-/congruent concepts and articulations (see legend)–i.e., none in this particular case. The reasoner infers 108 logically implied articulations that constitute the set of MIR. See also S2 File. Although the input and alignment of Figs 1 and 2 are empirically defensible, they fail to capture certain intuitions we have regarding the higher-level 2015./2014.Psittaciformes relationship. For instance, we may wish to say: "Sure, the author teams sampled complementary species-level concepts. Yet these trees are not actually in conflict. At higher levels, there likely is agreement that parrots are parrots, and non-parrots are non-parrots". That is: 2015.Psittaciformes = = 2014.Psittaciformes. To obtain this intuitive alignment, we have to *locally* relax coverage at select lower levels (Fig 3). In particular, 2015.PEA include five genus- and species-level concepts under 2015.Psittacidae that have no corresponding region under 2014.Psittacidae. However, if we relax coverage for 2014.Psittacidae–i.e., we assert 2014.nc_Psittacidae as an input constraint–then we can include each of these; for instance: 2015.Probosciger_aterrimus < 2014.Psittacidae, 2015.Psittacus_erithacus < 2014.Psittacidae, etc. Conversely, if we locally relax coverage for 2015.Psittacidae (2015.nc_Psittacidae), we can specify 2014.Melopsittacus_undulatus < 2015.Psittacidae. At the genus level, we can align 2015.Nestor = = 2014.Nestor if we relax coverage for each (2015.nc_Nestor, 2014.nc_Nestor), in spite of the mutually exclusive species-level concepts sampled. Jointly, these *four* instances of relaxing coverage render the articulation 2015.Psittacidae = = 2014.Psittacidae consistent, and hence also 2015.Psittaciformes = = 2014.Psittaciformes (Fig 4).

**Fig 3:**
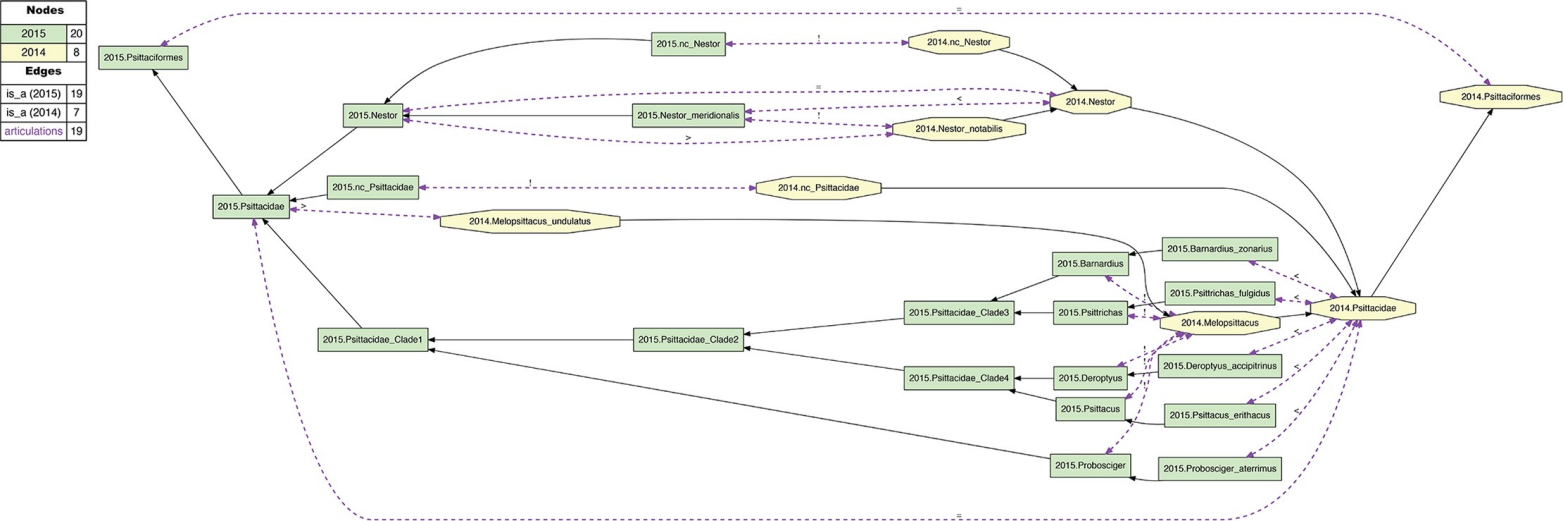
Input visualization for the 2015./2014.Psittaciformes alignment, with coverage locally relaxed. Compare with Fig 1. Here, coverage is relaxed for two family-level concepts (2015./2014.nc_Pittacidae) and two genus-level concepts (2015./2014.nc_Nestor). The eight species-level concepts of the alignment are correspondingly included as members of these higher-level concepts. In addition, three instances of congruence are asserted for 2015./2014.{Psittaciformes, Psittacidae, Nestor}. See also S3 File.

**Fig 4:**
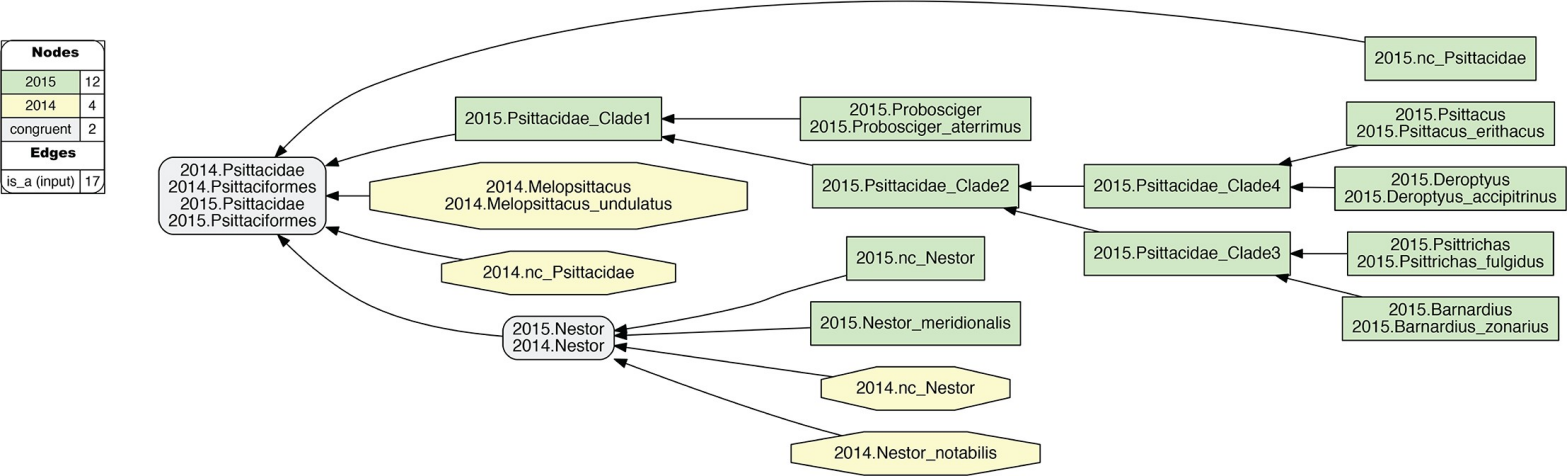
Alignment visualization for the 2015./2014.Psittaciformes alignment, with coverage locally relaxed. Compare with Fig 2. Local relaxing of coverage, and assertions of congruence of paired higher-level concepts (Fig 3), will yield the intuitive alignment of 2015.Psittaciformes = = 2014.Psittaciformes, 2015.Psittacidae = = 2014.Psittacidae, and 2015.Nestor = 2014.Nestor; in spite of wholly incongruent sampling of species-level concepts. The reasoner infers 160 logically implied articulations that constitute the set of MIR. See also S4 File.

The effect of locally relaxed coverage is illustrated in Figs 1–4, using the example of parrots– 2015./2014.Psittaciformes. At the species level, the author teams sampled wholly exclusive sets of concepts for this alignment region (Figs 1 and 3). Even at the genus level, only 2015./2014.Nestor is redundantly sampled, yet with the articulation: 2015.Nestor_meridionalis ! 2014.Nestor_notabilis at the child level. Therefore, if no species-level concept sec. 2015.PEA has an explicitly sampled and congruent region in 2014.Psittaciformes, and, vice-versa, no species-level concept sec. 2014.JEA has such a region in 2015.Psittaciformes, then under global application of the coverage constraint we obtain the alignment: 2015.Psittaciformes ! 2014.Psittaciformes (Fig 2). The absence of even partial concept region overlap at the terminal level 'propagates up' to the highest-level parent concepts, which are therefore also exclusive of each other.

Asserting higher-level node congruence in light of lower-level node incongruence requires a conception of node identity that affirms counter-factual statements of the following type: if 2014.JEA had sampled 2014.Psittacus_erithacus, then the authors would have included this species-level concept as a child of 2014.Psittacidae. This is to say that 2015./2014.Psittacidae, and hence their respective parents, are *intensionally* defined [25, 36, 37].

Using a combination of published topological information (and support), more or less direct reiterations of phenotypic traits (cf. discussions and supplementary data of 2015.PEA and 2014.JEA), and trained judgment [30], we align these concept regions as if there are congruent *property* criteria that each region entails, i.e., something akin to an implicit set of synapomorphies or uniquely diagnostic features. Of course, the phylogenomic data provided by 2015.PEA and 2014.JEA do not signal intensional definitions directly. But neither do their genome-based topologies for parrots provide evidence to challenge the status of such definitions as previously proposed [38]. In addition, particularly 2015.PEA (supplementary information; sections on "detailed justification for fossil calibrations" and "detailed phylogenetic discussion; pp. 3–21) provide a provide an in-depth account of how their preferred topology relates to published, property-centered circumscriptions of dozens of higher-level clade concepts. We have to assume, fallibly and non-trivially, that such topology-to-synapomorphy relations are also implied by JEA.2014, as reflected (*inter alia*) in their discussion.

Three clarifications are in order. First, Region Connection Calculus is at best a means of *translating* the signal of an intensional definition. The congruent (= =) symbol means, only: two regions are congruent in their extension. The RCC–5 vocabulary is obviously not appropriate for reasoning directly over genomic or phenomic property statements. The reasoner does not assess whether 2015.Psittacidae, or any included child or aligned concept, has 'the relevant synapomorphies'. Doing so would not be trivial even if property-based definitions were provided for all higher-level node concepts, because we would still have to make theory-laden assumptions about their congruent phylogenomic scopes [26, 39, 40]. Second, we are not providing detailed textual narratives that would *justify* each assertion of higher-level congruence. Such narratives are possible, and even needed to understand disagreements, because they explain the reasoning process behind an expert-made assertion. However, our main objective here is to focus on the issue of RCC–5 translation of systematic signals; not on a character-by-character dissection of each congruent articulation. Third, a sensible intensional alignment strategy uses a *minimal* number of instances of locally relaxed coverage in order to compensate for differential child sampling at lower levels, so that parent coverage can *remain in place* at higher levels to expose incongruent node concepts. The benefits of this strategy will be shown below.

**2. Representing clade concept labels.** Our modeling approach requires that every region in each source tree receives a taxonomic or clade concept label. However, the source publications only provide such labels for a subset of the inferred nodes. In particular, 2015.PEA (p. 570: Fig 1) obtained 41 nodes above the ordinal level. Of these, 17 nodes (41.5%) were explicitly labeled in either the published figure or supplement (pp. 9–12). The authors also cite [20] as the primary source for valid name usages, yet that list is not concerned with supra-ordinal names. Similarly, 2014.JEA (p. 1322: Fig 1) inferred 37 nodes above the ordinal level, of which 23 nodes (62.2%) were given an explicit label. They provide an account (cf. supplementary materials SM6: 22–24) of their preferred name usages, sourced mainly to [20] and [41].

In assigning clade concept labels at the supra-ordinal level when the authors may have failed to do so (consistently), we nevertheless made a good faith effort–through examination of the supplementary information and additional sources [1, 3, 42, 43, 44, 45, 46, 47]–to represent the authors' preferred name usages. Where usages were not explicit, we selected the only or most commonly applied clade concept name at the time of publication. This effort yielded 13 additional labels for 2015.PEA (Table 1), and 7 such labels for 2014.JEA (Table 2).

**Table 1:** Supra-ordinal clade concept labels used for the phylogenomic tree of 2015.PEA, with sources from which the names were obtained. "Franz et al. 2018" means: the label was assigned pragmatically in this study. See main text for further detail.

#	Clade concept label	Utilized name source	Immediate child concepts
**P01**	2015.Neornithes	Livezey & Zusi 2007 [43]	2015.Palaeognathae, 2015.Neognathae
**P02**	2015.Palaeognathae	Prum et al. 2015 [2]	2015.Notopalaeognathae, 2015.Struthioniformes
**P03**	2015.Notopalaeognathae	Yuri et al. 2013 [46]	2015.Novaeratitae, 2015.Rheiformes
**P04**	2015.Novaeratitae	Yuri et al. 2013 [46]	2015.Apterygiformes, 2015.Novaeratitae_Clade1
**P05**	2015.Novaeratitae_Clade1	Franz et al. 2018	2015.Casuariiformes, 2015.Tinamiformes
**P06**	2015.Neognathae	Jarvis et al. 2014 [1]	2015.Galloanserae, 2015.Neoaves
**P07**	2015.Galloanserae	Prum et al. 2015 [2]	2015.Anseriformes, 2015.Galliformes
**P08**	2015.Neoaves	Prum et al. 2015 [2]	2015.Strisores, 2015.Neoaves_Clade1
**P09**	2015.Strisores	Prum et al. 2015 [2]	2015.Caprimulgidae, 2015.Strisores_Clade1
**P10**	2015.Strisores_Clade1	Franz et al. 2018	2015.Nyctibiidae, 2015.Steatornithidae, 2015.Strisores_Clade2
**P11**	2015.Strisores_Clade2	Franz et al. 2018	2015.Apodiformes, 2015.Aegothelidae, 2015.Podargidae
**P12**	2015.Neoaves_Clade1	Franz et al. 2018	2015.Columbaves, 2015.Neoaves_Clade2
**P13**	2015.Columbaves	Prum et al. 2015 [2]	2015.Columbimorphae, 2015.Otidimorphae
**P14**	2015.Columbimorphae	Prum et al. 2015 [2]	2015.Columbiformes, 2015.Columbimorphae_Clade1
**P15**	2015.Columbimorphae_Clade1	Franz et al. 2018	2015.Mesitornithiformes, 2015.Pterocliformes
**P16**	2015.Otidimorphae	Prum et al. 2015 [2]	2015.Musophagiformes, 2015.Otidimorphae_Clade1
**P17**	2015.Otidimorphae_Clade1	Franz et al. 2018	2015.Mesitornithiformes, 2015.Ptercoclidiformes
**P18**	2015.Neoaves_Clade2	Franz et al. 2018	2015.Gruiformes, 2015.Neoaves_Clade3
**P19**	2015.Neoaves_Clade3	Franz et al. 2018	2015.Aequorlitornithes, 2015.Inopinaves
**P20**	2015.Aequorlitornithes	Prum et al. 2015 [2]	2015.Aequorlitornithes_Clade1, 2015.Ardeae
**P21**	2015.Aequorlitornithes_Clade1	Franz et al. 2018	2015.Charadriiformes, 2015. 2015.Phoenicopterimorphae
**P22**	2015.Phoenicopterimorphae	Jarvis et al. 2014 [1]	2015.Phoenicopteriformes, 2015.Podicipediformes
**P23**	2015.Ardeae	Brodkorb 1963 [42]	2015.Aequornithia, 2015.Phaethontimorphae
**P24**	2015.Aequornithia	Prum et al. 2015 [2]	2015.Aequornithia_Clade1, 2015.Gaviiformes
**P25**	2015.Aequornithia_Clade1	Franz et al. 2018	2015.Pelecanimorphae, 2015.Procellariimorphae
**P26**	2015.Pelecanimorphae	Prum et al. 2015 [2]	2015.Ciconiiformes, 2015.Pelecanimorphae_Clade1
**P27**	2015.Pelecanimorphae_Clade1	Franz et al. 2018	2015.Pelecaniformes, 2015.Suliformes
**P28**	2015.Procellariimorphae	Prum et al. 2015 [2]	2015.Procellariiformes, 2015.Sphenisciformes
**P29**	2015.Phaethontimorphae	Prum et al. 2015 [2]	2015.Eurypygiformes, 2015.Phaethontiformes
**P30**	2015.Inopinaves	Prum et al. 2015 [2]	2015.Opisthocomiformes, 2015.Telluraves
**P31**	2015.Telluraves	Prum et al. 2015 [2]	2015.Accipitriformes, 2015.Eutelluraves
**P32**	2015.Eutelluraves	Prum et al. 2015 [2]	2015.Australaves, 2015.Coracornithia
**P33**	2015.Australaves	Prum et al. 2015 [2]	2015.Cariamiformes, 2015.Eufalconimorphae
**P34**	2015.Eufalconimorphae	Suh et al. 2011 [45]	2015.Falconiformes, 2015.Passerimorphae
**P35**	2015.Passerimorphae	Sibley et al. 1988 [3]	2015.Passeriformes, 2015.Psittaciformes
**P36**	2015.Coracornithia	Claramunt & Cracraft 2015 [47]	2015.Coraciimorphae, 2015.Strigiformes
**P37**	2015.Coraciimorphae	Prum et al. 2015 [2]	2015.Coliiformes, 2015.Eucavitaves
**P38**	2015.Eucavitaves	Yuri et al. 2013 [46]	2015.Cavitaves, 2015.Leptosomiformes
**P39**	2015.Cavitaves	Yuri et al. 2013 [46]	2015.Picocoraciae, 2015.Trogoniformes
**P40**	2015.Picocoraciae	Mayr 2010 [44]	2015.Bucerotiformes, 2015.Picodynastornithes
**P41**	2015.Picodynastornithes	Yuri et al. 2013 [46]	2015.Coraciiformes, 2015.Piciformes

**Table 2:** Supra-ordinal clade concept labels used for the phylogenomic tree of 2014.JEA, with sources from which the names were obtained. "Franz et al. 2018" means: the label was assigned pragmatically in this study. See main text for further detail.

#	2014.JEA clade concept label	Utilized name source	Immediate child concepts
**J01**	2014.Neornithes	Jarvis et al. 2014 [1]	2014.Palaeognathae, 2014.Neognathae
**J02**	2014.Palaeognathae	Jarvis et al. 2014 [1]	2014.Struthioniformes, 2014.Tinamiformes
**J03**	2014.Neognathae	Jarvis et al. 2014 [1]	2014.Galloanseres, 2014.Neoaves
**J04**	2014.Galloanseres	Jarvis et al. 2014 [1]	2014.Anseriformes, 2014.Galliformes
**J05**	2014.Neoaves	Jarvis et al. 2014 [1]	2014.Columbea, 2014.Passerea
**J06**	2014.Columbea	Jarvis et al. 2014 [1]	2014.Columbimorphae, 2014.Phoenicopterimorphae
**J07**	2014.Columbimorphae	Jarvis et al. 2014 [1]	2014.Columbiformes, 2014.Columbimorphae_Clade1
**J08**	2014.Columbimorphae_Clade1	Franz et al. 2018	2014.Mesitornithiformes, 2014.Pterocliformes
**J09**	2014.Phoenicopterimorphae	Jarvis et al. 2014 [1]	2014.Phoenicopteriformes, 2014.Podicipediformes
**J10**	2014.Passerea	Jarvis et al. 2014 [1]	2014.Passerea_Clade1, 2014.Passerea_Clade4
**J11**	2014.Passerea_Clade1	Franz et al. 2018	2014.Passerea_Clade2, 2014.Passerea_Clade3
**J12**	2014.Passerea_Clade2	Franz et al. 2018	2014.Ardeae, 2014.Telluraves
**J13**	2014.Ardeae	Brodkorb 1963 [42]	2014.Aequornithia, 2014.Phaethontimorphae
**J14**	2014.Aequornithia	Jarvis et al. 2014 [1]	2014.Aequornithia_Clade1, 2014.Gaviimorphae
**J15**	2014.Aequornithia_Clade1	Franz et al. 2018	2014.Pelecanimorphae, 2014.Procellariimorphae
**J16**	2014.Pelecanimorphae	Jarvis et al. 2014 [1]	2014.Pelecaniformes
**J17**	2014.Procellariimorphae	Jarvis et al. 2014 [1]	2014.Procellariiformes, 2014.Sphenisciformes
**J18**	2014.Gaviimorphae	Jarvis et al. 2014 [1]	2014.Gaviiformes
**J19**	2014.Phaethontimorphae	Jarvis et al. 2014 [1]	2014.Eurypygiformes, 2014.Phaethontiformes
**J20**	2014.Telluraves	Jarvis et al. 2014 [1]	2014.Afroaves, 2014.Australaves
**J21**	2014.Afroaves	Jarvis et al. 2014 [1]	2014.Accipitrimorphae, 2014.Coracornithia
**J22**	2014.Accipitrimorphae	Jarvis et al. 2014 [1]	2014.Accipitriformes
**J23**	2014.Coracornithia	Claramunt & Cracraft 2015 [47]	2014.Coraciimorphae, 2014.Strigiformes
**J24**	2014.Coraciimorphae	Jarvis et al. 2014 [1]	2014.Coliiformes, 2014.Eucavitaves
**J25**	2014.Eucavitaves	Yuri et al. 2013 [46]	2014.Cavitates, 2014.Leptosomiformes
**J26**	2014.Cavitates	Yuri et al. 2013 [46]	2014.Picocoraciae, 2014.Trogoniformes
**J27**	2014.Picocoraciae	Mayr 2010 [44]	2014.Bucerotiformes, 2014.Picodynastornithes
**J28**	2014.Picodynastornithes	Yuri et al. 2013 [46]	2014.Coraciiformes, 2014.Piciformes
**J29**	2014.Australaves	Jarvis et al. 2014 [1]	2014.Cariamiformes, 2014.Eufalconimorphae
**J30**	2014.Eufalconimorphae	Suh et al. 2011 [45]	2014.Falconiformes, 2014.Passerimorphae
**J31**	2014.Passerimorphae	Jarvis et al. 2014 [1]	2014.Passeriformes, 2014.Psittaciformes
**J32**	2014.Passerea_Clade3	Franz et al. 2018	2014.Cursorimorphae, 2014.Opisthocomiformes
**J33**	2014.Cursorimorphae	Jarvis et al. 2014 [1]	2014.Charadriiformes, 2014.Gruiformes
**J34**	2014.Passerea_Clade4	Franz et al. 2018	2014.Caprimulgimorphae, 2014.Otidimorphae
**J35**	2014.Caprimulgimorphae	Jarvis et al. 2014 [1]	2014.Caprimulgiformes
**J36**	2014.Otidimorphae	Jarvis et al. 2014 [1]	2014.Cuculiformes, 2014.Otidimorphae_Clade1
**J37**	2014.Otidimorphae_Clade1	Franz et al. 2018	2014.Musophagiformes, 2014.Otidiformes

If no suitable label was available, we chose a simple naming convention of adding "_Clade1", "_Clade2", etc., to the available and immediately higher-level node label, e.g. 2014.Passerea_Clade1. The numbering of such labels along the tree topology starts with the most immediate child of a properly named parent, and typically follows down one section of the source tree entirely ("depth-first"), before continuing with the higher-level sister section. Using this approach, we added 11 labels for 2015.PEA (Table 1) and 7 labels for JEA.2014 (Table 2). If greater numbers of labels need to be generated, including siblings, then it is sensible to have a rule for ordering sibling nodes, e.g. by assigning the next-lowest number to the sibling whose child's name appears first in the alphabet. Our numbering of the labels 2014.Passerea_Clade2 (child with first-appearing letter: 2014.Ardeae) and 2014.Passerea_Clade3 (child: 2014.Cursorimorphae) adhere to this rule.

The clade concept labeling convention was not applied below the family level, where instead phylogenomic resolution was collapsed into polytomy (exception: Figs 1–4). In the case of 2014.JEA, only four family-level concepts include two children, whereas the remainder have a single child sampled. Resolving the monophyly of subfamilial clade concepts was not the primary aim of 2014.JEA. The same applies to 2015.PEA, who sampled 104/125 family-level concepts with only 1–2 children.

**Fig 5:**
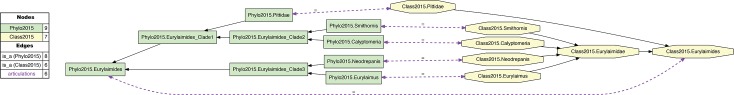
Input visualization of the alignment of the phylogenomic reconstruction of passeriform *clade* concepts sec. 2015.PEA–prefixed with "Phylo2015"–with the corresponding *classification* concepts sec. Gill & Donsker (2015) [20]–prefixed with "Class2015". The phylogenomic topology renders that of Class2015.Eurylaimidae paraphyletic, and hence the name "Eurylaimidae" is not represented in any clade concept label sec. 2015.PEA. See also Prum et al. (2015). See also S5 File.

**Fig 6:**
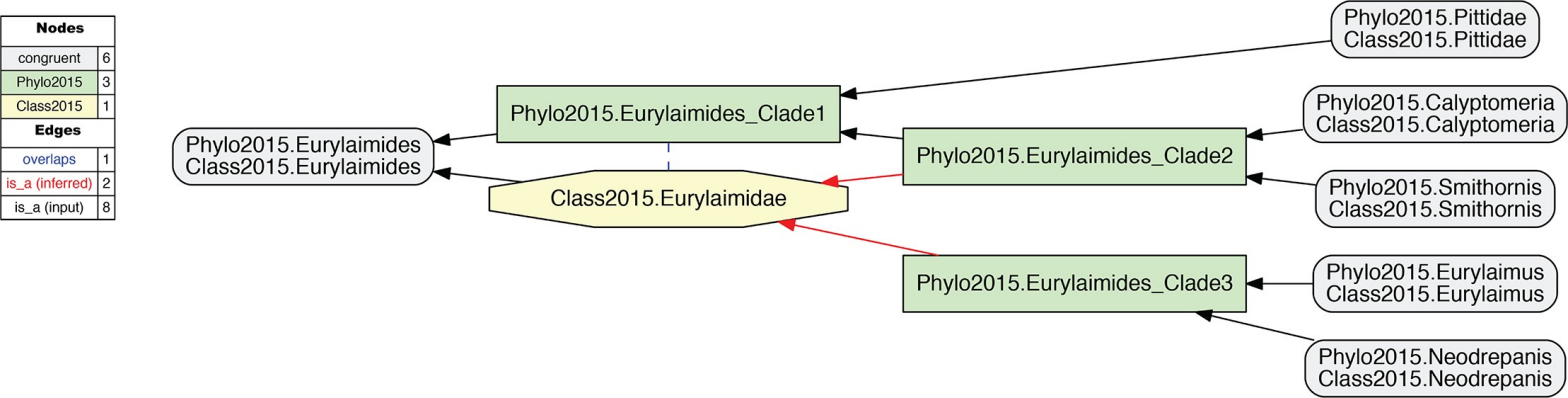
Alignment visualization corresponding to Fig 5. The alignment shows an overlapping articulation (dashed blue line) between the phylogenomic clade concept sec. 2015.PEA (Phylo2015.Passeriformes_Clade1) and the Eurylaimidae sec. Gill & Donsker (2015) [20] (Class2015.Eurylaimidae). The two dashed red arrows symbolize reasoner-inferred relationships not explicit in the input constraints. See also S6 File.

**3. Representing phylogeny/classification paraphyly.** A third, relatively minor challenge is the occurrence of clade concepts in 2015.PEA's phylogenomic tree that are not congruently aligned with higher-level concepts of [20]. We highlight these instances here because they represent a widespread phenomenon in phylogenomics. It is useful to understand how such discrepancies can be modeled with RCC–5 alignments (Figs 5 and 6).

Fig 5 exemplifies the phylogenetic tree/classification incongruence observed in 2015.PEA. The authors state (supplementary Table 1, p. 1): "Taxonomy follows Gill and Donsker (2015; fifth ed)". As shown in Fig 5, their phylogeny accommodates four sampled genus-level concepts that would correspond to children of the family-level concept Eurylaimidae sec. Gill & Donsker (2015) [20]. However, these concepts are arranged paraphyletically in relation to the reference classification. There is no parent concept that can be labeled 2015.Eurylaimidae and would not also (1) include 2015.Pittidae, i.e., 2015.Passeriformes_Clade1 in Fig 6, or (2) just represent aligned subset of the Eurylaimidae sec. Gill and Donsker (2015) [20], i.e., 2015.Passeriformes_Clade2 or 2015.Passeriformes_Clade3 in Fig 6. The concept Eurylaimidae sec. Gill and Donsker (2015) [20] has an overlapping (><) articulation with 2015. Passeriformes_Clade1.

In summary, our approach represents non-monophyly as an incongruent alignment of the phylogenomic tree and the source classification used to provide labels for that tree's monophyletic clade concepts. There are four distinct regions in the phylogeny of 2015.PEA where such alignments are needed: {Caprimulgiformes, Eurylaimidae, Hydrobatidae, Procellariidae, Tityridae} sec. Gill & Donsker (2015) [20]. Each of these is provided in the S7–S9 Files.

### Configuration of input constraints and alignment partitioning

The source phylogenies specify 703 and 216 clade or taxonomic concepts, respectively. The frequent instances of locally relaxed coverage increase the reasoning complexity in relation to multi-classification alignments [14], making specialized RCC–5 reasoning useful [48]. The reasoning and visualization challenges commend a partitioned alignment approach. To keep the Results concise, we show visualizations of the larger input and alignment partitions only in the Supporting Information. A detailed account of the input configuration and partitioning workflow is given below.

Underlying all alignments is the presumption that at the terminal (species) level, the taxonomic concept labels of 2015.PEA and 2014.JEA are reliable indicators of either pairwise congruence or exclusion [14, 26, 32]. That is, e.g., 2015.Cariama_cristata = = 2014.Cariama_cristata, or 2015.Charadrius_hiaticula ! 2014.Charadrius_vociferus. Because the time interval separating the two publications is short in comparison to the time needed for taxonomic revisions to effect changes in classificatory practice, the genus- or species-level taxonomic concepts are unlikely to show much incongruence; though see [49] or [50]. We note that 2015.PEA (p. 571) use the label 2015.Urocolius(_indicus) in their phylogenomic tree, which also corresponds to the genus-level name endorsed in [20] Gill & Donsker (2015). However, in their Supplementary Table 1 the authors use 2015.Colius_indicus. We chose 2015.Urocolius and 2015.Urocolius_indicus as the labels to apply in the alignments.

The toolkit workflow favors a partitioned, bottom-up approach [29]. The process of generating, checking, and regenerating input files must be handled 'manually' on the desktop (note: improved workflow documentation and semi-automation of input-output-input changes are highly desirable). The performance of different toolkit reasoners was benchmarked in [28].

To work efficiently, the large problem of aligning all concepts at once is broken down into multiple smaller alignment problems, e.g. 2015./2014.Psittaciformes (Figs 3 and 4). To manage one particular order-level alignment, we start with assembling each input phylogeny separately, with relaxed coverage applied as needed (Fig 3). The RCC–5 articulations for low-level concept pairs are provided incrementally, e.g., in sets of 1–5 articulations at a time. Following such an increment, the toolkit reasoning process is re-/deployed to validate input consistency and infer the number of possible worlds. There is an option to specify that only one possible world is sought as output, which is equivalent to *just* checking for input consistency, as opposed to inferring all possible worlds. Doing so saves time as long as the input remains (vastly) under-specified. The stepwise approach of adding a small number of articulations at a time leads to increasingly constrained alignments, while minimizing the risk of introducing many new. difficult-to-diagnose inconsistencies.

Once a set of small, topographically adjacent alignment partitions is well specified, these can serve as building blocks for the next, larger partition. Hence, the basic sequence of building up larger alignments is: (1) obtain a well-specified low- (order- or family-) level alignment; (2) record the inferred parent-level articulations from this alignment; (3) propagate the latter–now as low-level *input* articulations–for the next, more inclusive alignment; (4) as needed, prune the lowest-level (sub-ordinal) input concepts and articulations of (1) from this alignment; (5) repeat (1) to (4) for another paired region; (6) assemble the more inclusive alignment by (manually) connecting the pruned, propagated concepts and articulations from two or more lower-level alignments, by adding to them the higher-level concepts from each input phylogeny. Depending on the interplay between (ranked) higher-level names recognized in each phylogeny and the number of terminal concepts sampled, steps (1) to (6) may be iterated once (e.g., 2015./2014.{Falconiformes, Psittaciformes}) or multiple times (e.g., 2015./2014.Passeriformes) to cover a supra-/ordinal alignment. An example of the latter is the 2015./2014.Passerimorphae alignment, which includes two order-level concepts and their children in each source phylogeny. Such mid-level partitions eventually form the basis for the largest alignment partitions, e.g. 2015./2014.Telluraves.

Sometimes, coverage will have to be relaxed even at higher levels. In all, 2014.JEA sample children of 34 order-level concepts in their phylogeny, whereas 2015.PEA recognize 40 order-level concepts. The latter authors represent four order-level concepts for which no analogous children are included in 2014.JEA, i.e.: 2015.{Apterygiformes, Casuariiformes, Ciconiiformes, Rheiformes}. Three of these are assigned to 2015.Palaeognathae, whereas 2015.Ciconiiformes are subsumed under 2015.Pelecanimorphae–in each case under relaxed parent coverage. The remaining 36 order-level concepts sec. 2015.PEA show some child-level overlap with those of 2014.JEA.

Our partitioning approach for this use case started with specifying the input constraints for nearly 35 paired order-level concepts and their respective children, as demonstrated in Figs 3 and 4. The largest order-level partition is 2015./2014.Passeriformes, with 148 x 22 input concepts, seven instances of relaxed parent coverage, and 101 input articulations. This alignment completes in less than 15 seconds on an individual 2.0 GHz processor, yielding 3,256 MIR.

As the partitions grew, we configured the following six, non-overlapping alignments as building blocks for the global alignment: 2015./2014.Palaeognathae (34 x 12 input concepts, four instances of relaxed coverage, and 25 articulations; same data sequence used for following alignments), 2015./2014.Galloanserae (49, 16, 7, 46), 2015.Columbaves/2014.Columbimorphae + 2014.Otidimorphae (53, 37, 13, 37), 2015.Strisores/2014.Caprimulgimorphae (44, 17, 8, 32), 2015./2014.Ardeae (100, 55, 19, 75), and the largest partition of 2015./2014.Telluraves (316, 104, 37, 241).

At the next more inclusive level, the inferred congruence of 2015.Telluraves = = 2014.Telluraves presented an opportunity to partition the entire alignment into two similarly sized regions, where the complementary region includes all 2015./2014.Neornithes concepts (392, 174, 58, 259), *except* those subsumed under 2015./2014.Telluraves, which are therein only represented with two concepts labels and one congruent articulation. These two complements are the core partitions that inform our use case alignment, globally. The corresponding S10 and S11 Files include the input constraint (.txt) and visualization (.pdf) files, along with the alignment visualization (.pdf) and MIR (.csv).

The two large partitions yield unambiguous RCC–5 articulations from the species concept level to that of 2015./2014.Neornithes. They can be aggregated into a synthetic, root-to-order level alignment, where all subordinal concepts and articulations are secondarily pruned away (see above). Such an alignment retains the logic signal derived from the bottom-up approach, but represents only congruent order-level concept labels as terminal regions, except in cases where there is incongruence. We present this alignment as an analogue to Fig 1 in [4] (p. 515), and compare how each conveys information about congruent and conflicting higher-level clade concepts.

Lastly, we further reduce the root-to-order alignment to display only 5–6 clade concept levels below the congruent 2015./2014.Neoaves. This region of the alignment is the most conflicting, and therefore forms the basis for our Discussion.

## Results

### Higher-level congruence

Our alignments show widespread higher-level congruence across the neoavian explosion use case; along with several minor regions of conflict and one strongly conflicting region between concepts placed immediately below the 2015./2014.Neoaves.

We focus first on the large complementary partitions, i.e. 2015./2014.Neornithes (without) / 2015./2014.Telluraves (see S10 and S11 Files). Jointly, they entail 707 concepts sec. 2015.PEA and 283 concepts sec. 2014.JEA. Among these, 34 "no coverage" regions were added to 2015.PEA's phylogeny, whereas 61 instances of relaxing parent coverage were assigned to 2014.JEA's phylogeny, for a total of 95 instances of relaxing this constraint. The 2015./2014.Neornithes partition shows 305 aligned regions– 247 without the "no coverage" regions–of which 60 congruently carry at least one concept label from each source phylogeny. This alignment also shows eight congruent species-level concept regions. These would be the *only* instances of congruence if coverage were globally applied (Figs 1 and 2). Therefore, relaxing the coverage constraint yields 52 *additional* instances of higher-level node congruence. Similarly, the 2015./2014.Telluraves partition has 231 aligned regions– 194 without the "no coverage" regions–of which 38 are congruent. This corresponds to an increase of 34 regions, compared to four congruent species-level concept regions present under strict coverage. Correcting for the redundant 2015./2014.Telluraves region, we 'gain' 85 congruent parent node regions across the two phylogenies *if* node identity is encoded intensionally (Figs 3 and 4). Indeed, this approach yields the intuitive articulation 2015.Neornithes = = 2014.Neornithes at the highest level.

### Two kinds of conflict: Differential granularity and overlap

We now focus on characterizing the conflict between 2015.PEA and 2014.JEA. Phylogenomic incongruence can be divided into two general categories: (1) differential granularity or resolution of clade concepts (RCC–5 translation: < or >), and (2) overlapping clade concepts (RCC–5 translation: ><).

The first of these is less problematic from a standpoint of achieving integration: for a given alignment subregion, the more densely sampled phylogeny will entail additional, more finely resolved clade concepts in comparison to its counterpart. Typically, this distinction belongs to the phylogeny of 2015.PEA, due to the 4:1 ratio of terminals sampled. There are 83 above species-level clade concepts sec. 2015.PEA that can be interpreted as *congruent refinements* of the 2014.JEA topology (see S10 and S11 Files). Conversely, only two such instances of added resolution are contributed by 2014.JEA: (1) 2014.Passeriformes_Clade3 which entails 2014.Passeridae and 2014.Thraupidae; and (2) 2014.Haliaeetus with two subsumed species-level concepts. Nevertheless, the joint 97 congruent node regions and 85 refining node regions cover a large section of the alignment where integration is either reciprocally (= =) or unilaterally (< or >) feasible.

### Topological overlap

**Fig 7:**
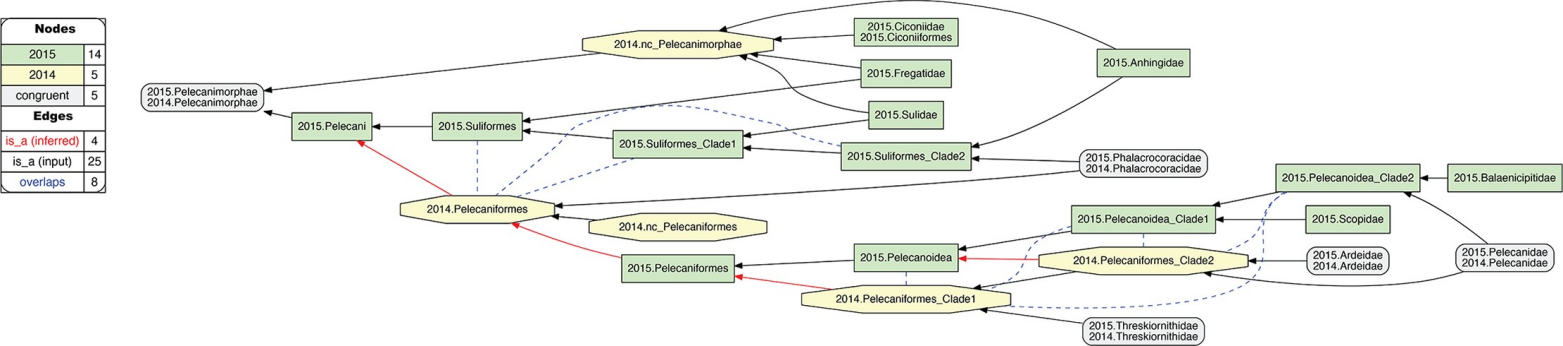
Alignment visualization for the 2015./2014.Pelecanimorphae alignment, with eight overlapping relationships. See text for further detail. The reasoner infers 200 logically implied articulations that constitute the set of MIR. See also S12 File.

**Fig 8:**
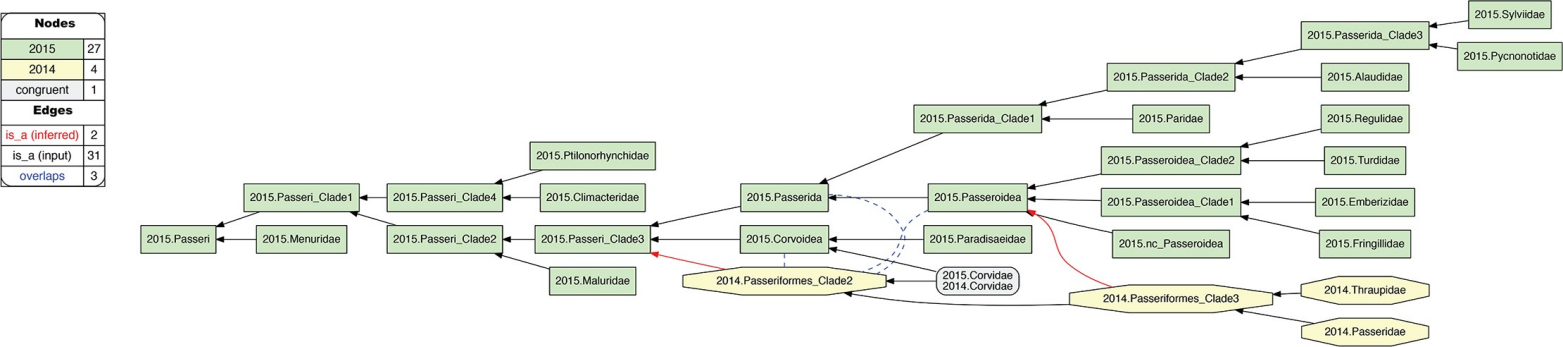
Alignment visualization for the 2015.Passeri/2014.Passeriformes_Clade2 alignment, with three overlapping relationships. See text for further detail. The reasoner infers 135 logically implied articulations that constitute the set of MIR. See also S13 File.

**Fig 9:**
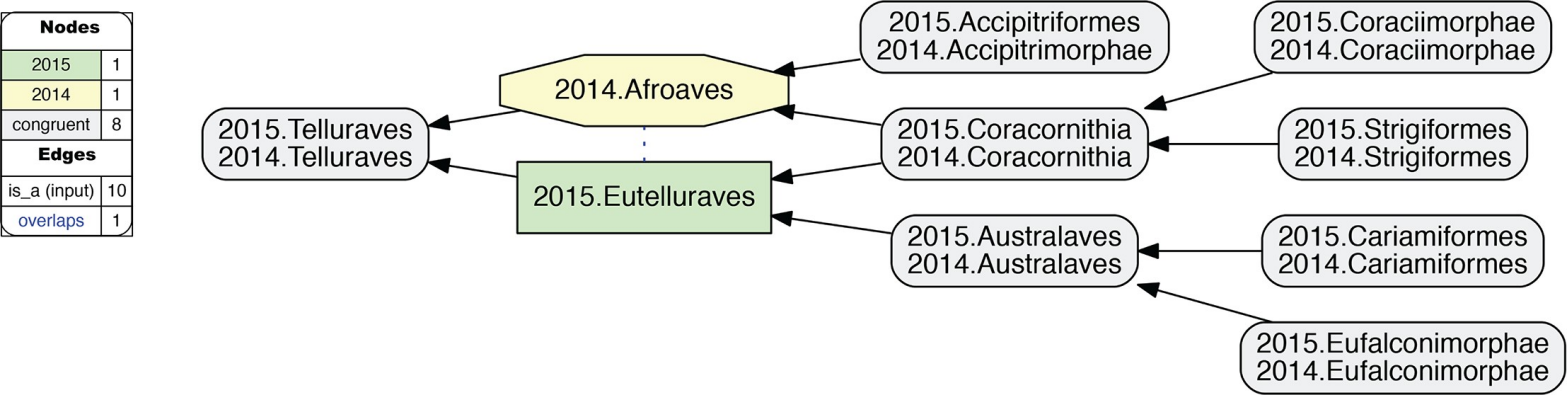
Alignment visualization for the 2015./2014.Telluraves alignment, under whole-concept resolution, with one overlapping relationship. Compare with Fig 10; see text for further detail. The reasoner infers 81 logically implied articulations that constitute the set of MIR. See also S14 File.

**Fig 10:**
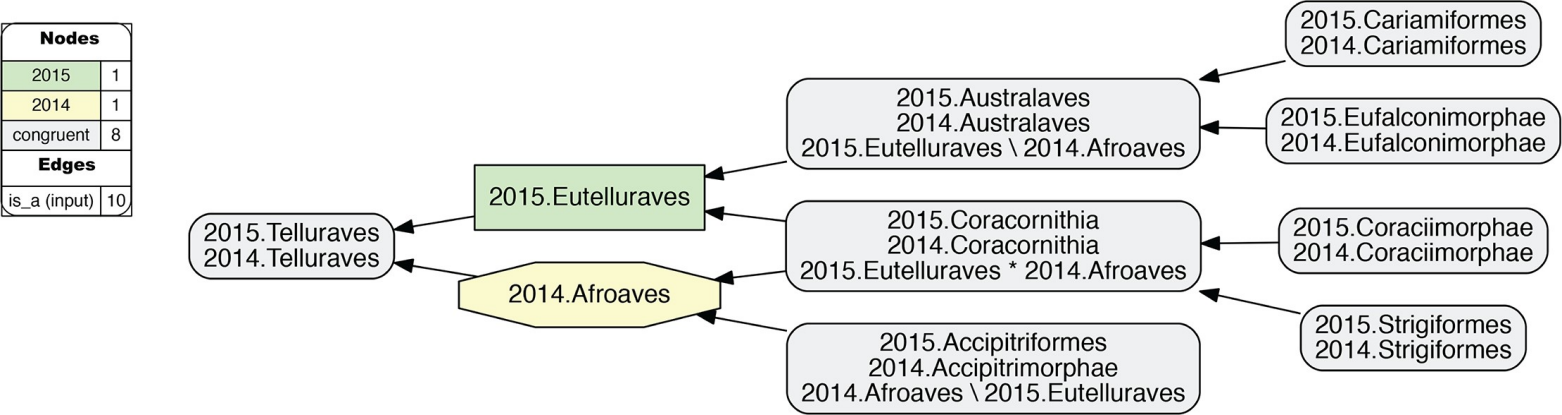
Alignment visualization for the 2015./2014.Telluraves alignment, under split-concept resolution, resolving the overlapping relationship. Compare with Fig 9; see text for further detail. The reasoner infers 81 logically implied articulations that constitute the set of MIR. See also S15 File.

**Fig 11:**
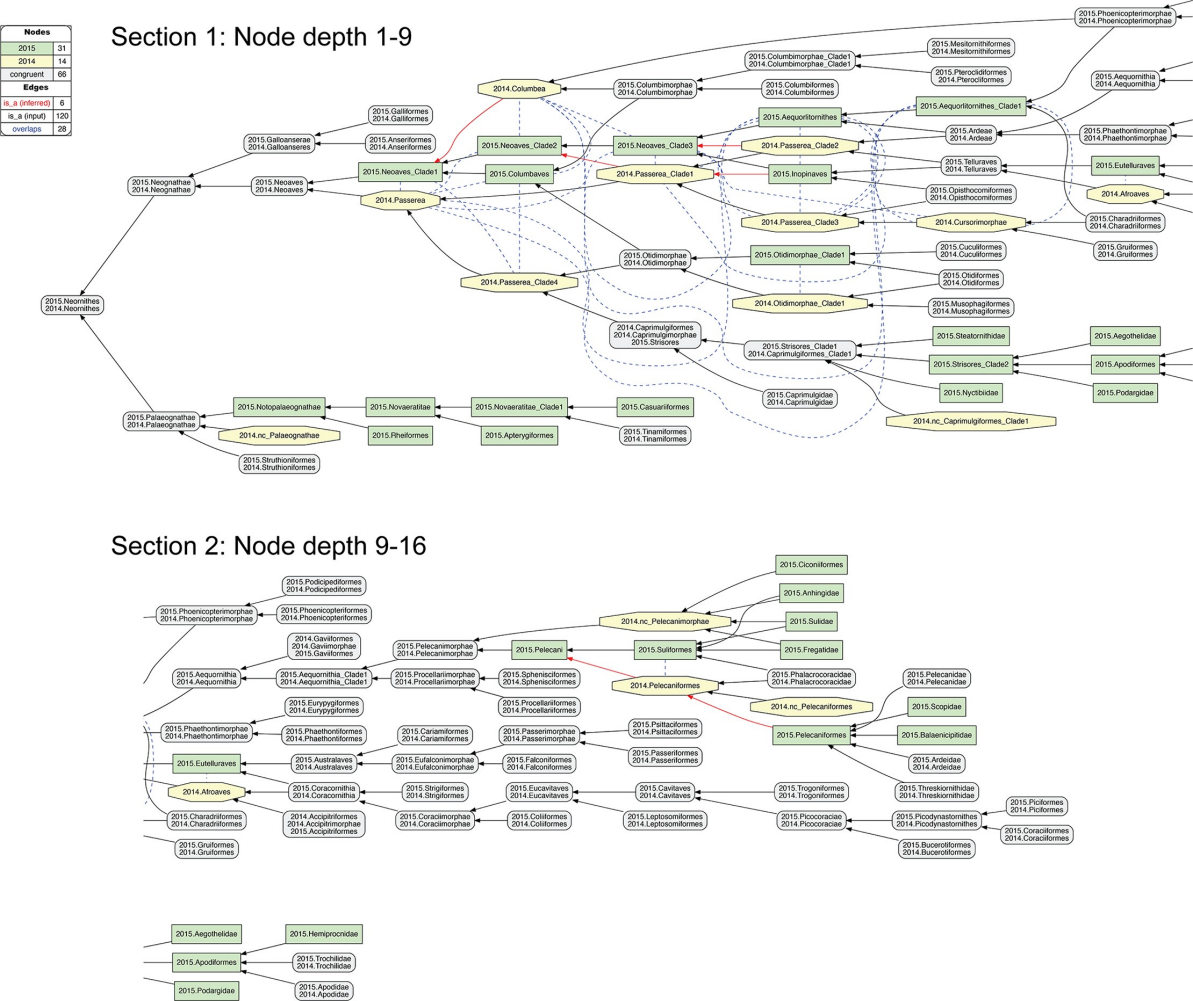
Alignment visualization for the 2015./2014.Neornithes alignment, under whole-concept resolution, ranging from the root to the ordinal level (with exceptions where needed), and with 28 overlapping relationships. Compare with Figs 7, 9 and 10; see text for further detail. The reasoner infers 8,051 logically implied articulations that constitute the set of MIR. See also S16 File.

**Fig 12:**
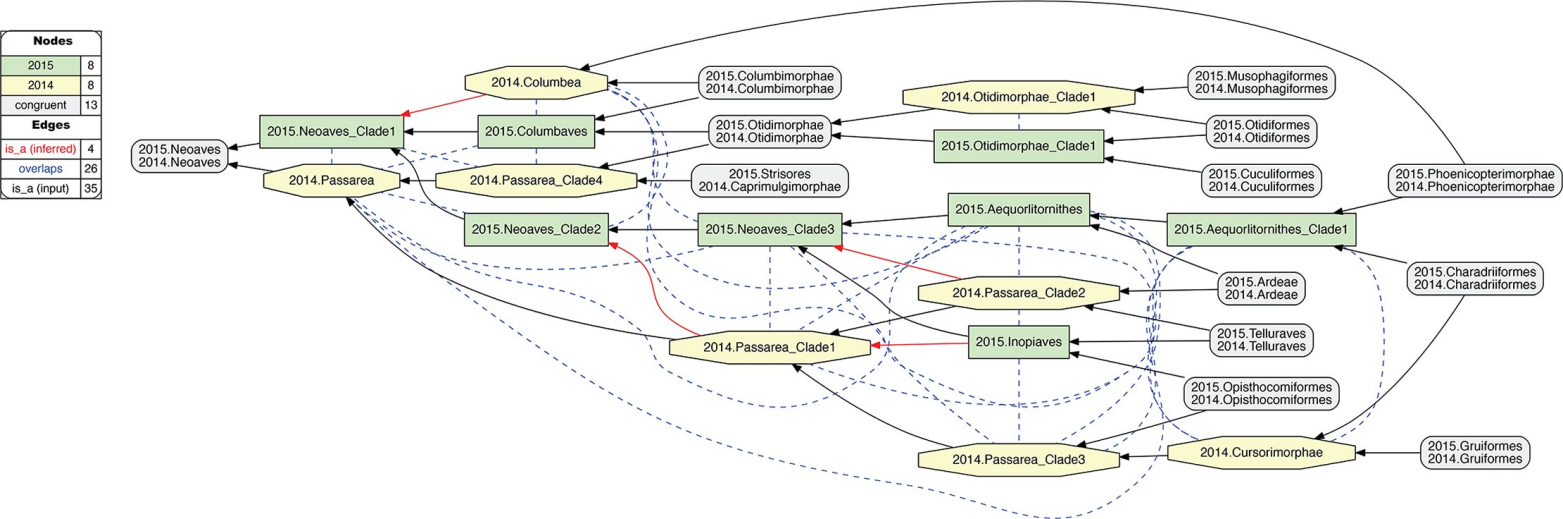
Alignment visualization for the 2015./2014.Neoaves alignment, under whole-concept resolution, limited to the main conflict region, and with 26 overlapping relationships. Compare with Fig 11. The reasoner infers 441 logically implied articulations that constitute the set of MIR. See also S17 File.

**Fig 13:**
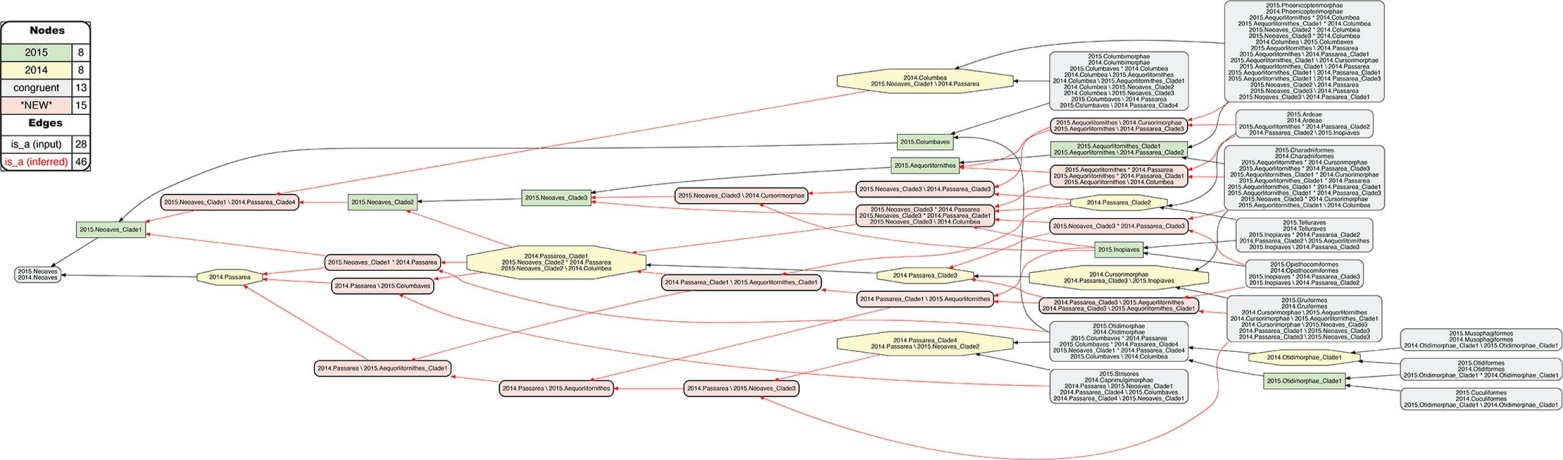
Alignment visualization for the 2015./2014.Neoaves alignment, under split-concept resolution, limited to the main conflict region, and resolving the 26 overlapping relationships. Compare with Fig 12; the 15 salmon-colored regions are *only* identifiable via split-concept resolution labels. Compare with Fig 12 and Table 4. See text for further detail. The reasoner infers 441 logically implied articulations that constitute the set of MIR. See also S18 File.

The remaining 38 instances of overlapping articulations between constitute the most profound conflict. These instances are clustered in four distinct regions, i.e.: 2015./2014.Pelecanimorphae (8 overlaps; Fig 7 and S12 File); 2015.Passeri/2014.Passeriformes_Clade2 (3 overlaps; Fig 8 and S13 File); 2015.Eutelluraves/2014.Afroaves (1 overlap; Figs 9 and 10, and S14 and S15 Files); and finally, 2015./2014.Neoaves (26 overlaps; Figs 11–13, and S16–S18 Files). We will examine each of these in sequence.

**1. 2015./2014.Pelecanimorphae.** The two author teams sampled four family-level concepts congruently for this alignment region (Fig 7). However, 2015.PEA's phylogeny entails six additional family-level concepts that have no apparent match in 2014.JEA. Moreover, the latter authors recognize only one order-level concept, 2014.Pelecaniformes, under which all four family-level concepts are subsumed, including 2014.Phalacrocoracidae. In contrast, 2015.PEA infer an intensionally less inclusive concept of 2015.Pelecaniformes, and place their congruent 2015.Phalacrocoracidae in the order-level concept 2015.Suliformes. This is the first instance where we may plausibly reject the proposition: "Had 2014.JEA sampled 2014.Phalacrocoracidae, they would have assigned this concept to 2014.Suliformes". The assertion is no longer counter-factual: 2014.JEA *did* sample the corresponding child concept (2014.Phalacrocoracidae), but did *not* assign it to a parent concept separate from 2014.Pelecaniformes. Accordingly, we obtain three overlapping, 'cascading' articulations between concepts that form the 2015.Suliformes higher-level topology and 2014.Pelecaniformes. Meanwhile, the uniquely sampled 2015.Ciconiiformes are subsumed under 2014.Pelecanimorphae with relaxed parent coverage.

Within 2015.Pelecaniformes, we obtain five additional overlapping articulations between five concepts that make up the 2015/2014 supra-familial topologies in this alignment (Fig 7). This conflict is due to the differential assignment of 2015./2014.Pelecanidae. Specifically, 2015.PEA inferred a sister relationship of 2015.Pelecanidae with 2015.Balaenicipitidae, for which 2014.JEA have no sampled match. Meanwhile, the latter authors inferred a sister relationship of 2014.Pelecanidae with 2014.Ardeidae. The latter concept *is* matched in 2015.PEA with 2015.Ardeidae, though not as the most immediate sister concept of 2015.Pelecanidae. Of course, we may posit that a 2015.Ardeidae/2015.Pelecanidae sister relationship is what 2015.PEA *would* have obtained, had these authors not also sampled 2015.Balaenicipitidae and 2015.Scopidae. But they did, and hence obtained two clade concepts that include 2015.Pelecanidae yet exclude 2015.Ardeidae; i.e., 2015.Pelecanoidea_Clade1 and 2015.Pelecanoidea_Clade2. While relaxing parent coverage for 2014.Pelecaniformes_Clade2 could serve to mitigate this conflict, we deem the overlapping relationship to better represent 2015.PEA's phylogenomic signal, which happens to 'break up' the lowest supra-familiar clade concept supported by 2014.PEA.

**2. 2015.Passeri/2014.Passeriformes_Clade2.** This alignment region is another instance where relaxing parent coverage can only partially mitigate conflict (Fig 8). In this case, 2015.PEA and 2014.JEA sampled two sets of family-level concepts that are wholly exclusive of each other, *except* for 2015./2014.Corvidae. Regarding the only two additional family-level concepts recognized in 2014.JEA–i.e., 2014.Passeridae and 2014.Thraupidae–we may posit counter-factually that these would be subsumed under 2015.Passeroidea with relaxed coverage [47]. However, further assertions of congruence are difficult to justify, given the limited sampling of 2014.JEA. Thus, in our current representation, 2014.Passeriformes_Clade2 shows an overlapping relationship with 2015.Passeroidea, its immediate parent 2015.Passerida, and also with 2015.Corvoidea.

**3. 2015.Eutelluraves/2014.Afroaves.** A single overlap occurs just within the congruent parent concepts 2015./2014.Telluraves (Fig 9). Two levels below this paired parent region, both author teams recognize three congruent children; viz. 2015./2014.{Australaves, Coracornithia, Accipitrimorphae/Accipitriformes}. However, 2015.Prum group the former two concepts under 2015.Eutelluraves, with 2015.Accipitriformes as sister; whereas 2014.JEA cluster the latter two concepts under 2014.Afroaves, with 2014.Australaves as sister. This is the first occurrence of conflict that *cannot* justifiably be resolved by relaxing parent coverage, but instead reflects divergent phylogenomic signals.

### Whole-concept and split-concept resolution

How to *speak* of such overlap? In Fig 9, we utilize clade concept labels that pertain to each input phylogeny. In the resulting alignment, the articulation 2015.Eutelluraves >< 2014.Afroaves is visualized as a dashed blue line between these regions. Yet Fig 9 also specifies the extent of regional overlap at the next lower level. Accordingly, only the region 2015./2014.Coracornithia is subsumed under each of the overlapping parents. This is indicated by the two inclusion arrows that extend 'upward' from this region. The other two paired child regions are respectively members of one parent region.

If we call the input regions 2015.Eutelluraves "A" and 2014.Afroaves "B", we can use the following syntax to identify output regions that result from overlapping input concepts [26]: A*B (read: "A *and* B") constitutes the output region shared by two parents, whereas A\b ("A, *not* b") and B\a ("B, *not* a") are output regions with only one parent. We call this more granular syntax *split-concept resolution* ("merge concepts" in [26]), as opposed to *whole-concept resolution* which preserves the syntax and granularity provided by the input concept labels.

In Fig 10, the 2015./2014.Telluraves overlap is represented with split-concept resolution. This eliminates the need to visualize a dashed blue line between 2015.Eutelluraves and 2014.Afroaves (Fig 9). Moreover, in this case the split-concept resolution syntax is redundant or unnecessary, because each of the three resolved regions under "A" (2015.Eutelluraves) and "B" (2014.Afroaves) is congruent with two regions already labeled in the corresponding input phylogenies. We will see, however, that this granular syntax is essential for verbalizing the outcomes of more complex alignments that contain many overlapping regions.

### Zooming in on the neoavian explosion

**4. 2015./2014.Neoaves.** The remaining 26 instances of overlap are shown under different alignment visualizations in Figs 11–13. They occur 1–5 levels below the congruent concept pair 2015./2014.Neoaves, and jointly make up the primary region of conflict between these reconstructions. Because parent coverage was already and selectively applied at lower levels, none of the 26 overlaps in the alignment are caused by differential child sampling. Therefore parent coverage must *hold* here, resulting in genuine conflict in the higher-level arrangement of congruent sets of children.

Our Fig 11 is intended to be an RCC–5 alignment *analogue* to Fig 1 in [3]. The alignment reaches from the root to the ordinal level, and to the family level in the two subregions where order-level concepts are conflicting (see Fig 4 and S12 File). The visualization provides an intuitive signal of the distribution of in-/congruence throughout the alignment. In all, 66/111 regions (59.5%) are congruent, of which 22 are located in the 2015./2014.Telluraves; 15 are contained in the 2015./2014.Ardeae; and 5 are part of the 2015./2014.Columbimorphae. Outside of the 2015./2014.Neoaves, 8 such regions are present. In other words, the two phylogenies are congruent at the highest level and also in several intermediate regions above the ordinal level.

Fig 12 shows just the neoavian explosion region under whole-concept resolution. Each phylogeny contributes 21 input concepts to this 'zoomed-in' alignment, which yields 13 congruent regions. Of these, only 2015./2014.Neoaves and 2015./2014.Otidimorphae represent non-terminal concepts.

Unpacking the complexity of this conflict region requires a stepwise analysis. From the perspective of 2015.PEA, the 2015.Neoaves are split into a sequence of three unnamed, higher-level clade concepts, i.e. 2015.{Neoaves_Clade1, Neoaves_Clade2, Neoaves_Clade3}, with 2015.{Strisores, Columbaves, Gruiformes} as corresponding sister concepts. The two children of 2015.Neoaves_Clade3 are 2015.{Aequorlitornithes, Inopinaves}. The authors accept the nomenclature of [44] for 2015.Strisores, with is congruent with 2014.Caprimulgimorphae; and the region 2015./2014.Gruiformes is congruent as well. However, the remaining six high-level concepts of 2015.PEA are in conflict with the two highest-level neoavian concepts of 2014.JEA, i.e. 2014.{Columbea, Passerea}, and also with any of the four unnamed clade concepts below 2014.Passerea. In particular, the node sequence 2015.{Neoaves_Clade3, Aequorlithornites, Aequorlithornites_Clade1} participates in 16/26 overlaps, as summarized in Table 3. Loosely corresponding to this sequence are the concepts 2014.{Passerea_Clade1, Passerea_Clade2, Cursorimorphae}, jointly with 10 overlaps. These overlaps are grounded in the incongruent assignment of five paired, lower-level concept regions; viz. 2015./2014.{Ardeae, Charadriiformes, Opisthocomiformes, Phoenicopterimorphae, Telluraves}. Two conflicting placements contribute most to the number of overlaps: (1) 2015./2014.Charadriiformes in 2015.Aequorlithornites_Clade1 (sister to 2015.Phoenicopterimorphae) versus 2014.Cursorimorphae (sister to 2014.Gruiformes); and (2) 2015./2014.Phoenicopterimorphae in 2015.Aequorlithornites_Clade1 versus 2014.Columbea (sister to 2014.Columbimorphae). The newly proposed yet unnamed 2015. Aequorlithornites_Clade1, consisting of certain "waterbirds", in effect causes the most topological incongruence with 2014.JEA. This concept, together with its four superseding parents, 'triggers' 20/26 overlaps with the phylogenomic tree of 2014.JEA.

**Table 3:** Overview of 26 pairwise 2015/2014 concept overlaps in main neoavian conflict region. See also Figs 11 and 12.

Clade concept label	2014. Passerea	2014. Columbea	2014.Passerea _Clade3	2014.Cursori- morphae	2014.Passerea _Clade1	2014.Passerea _Clade2	2014.Passerea _Clade4	2014.Otidimor- phae_Clade1	Totals
**2015.Aequorlithornites**	**><**	**><**	**><**	**><**	**><**	**><**			**6**
**2015.Aequorlithornites_Clade1**	**><**	**><**	**><**	**><**	**><**				**5**
**2015.Neoaves_Clade3**	**><**	**><**	**><**	**><**	**><**				**5**
**2015.Columbaves**	**><**	**><**					**><**		**3**
**2015.Neoaves_Clade1**	**><**						**><**		**2**
**2015.Neoaves_Clade2**	**><**	**><**							**2**
**2015.Inopinaves**			**><**			**><**			**2**
**2015.Otidimorphae_Clade1**								**><**	**1**
**Totals**	**6**	**5**	**4**	**3**	**3**	**2**	**2**	**1**	**26**

Two additional clusters of conflict are identifiable in Fig 12. The first concerns the alignment of the two concepts 2015.Inopinaves and 2014.Passerea_Clade2, which share the child regions 2015./2014.Telluraves, yet which differentially accommodate the congruent regions 2015./2014.Ardeae and 2015./2014.Opisthocomiformes. This further contributes to the abundance of overlaps along the respective 2015.Neoaves_Clade{1–3}/Aequorlithornites/_Clade1 and 2014.Passerea/_Clade{1–3}/Cursorimorphae chains. Second, the two paired regions 2015./2014.Columbimorphae and 2015./2014.Otidimorphae are incongruently assigned to three overlapping parents, i.e. 2015.Columbaves and 2014.{Columbea, Passerea_Clade4}. From the perspective of 2015.PEA, 2014.JEA's bifurcation of 2014.Columbea and 2014.Passerea is the most conflicting, as these two concepts participate in 11 overlaps. A third and more minor incongruence concerns the placement of three concept regions within the 2015./2014.Oditimorphae.

### Split-concept resolution for the neoavian explosion

In Fig 13, the same 'zoomed-in' alignment is shown under split-concept resolution. This permits identifying all output regions created by the 26 overlaps of the neoavian explosion (see Table 4). The entire set consists of 78 labels; i.e., 26 labels for each split-resolution product {A*B, A\b, B\a} for one instance of input region overlap. Not all of these split-concept resolution labels are semantically redundant with those provided in the input. Specifically, 51 labels are generated 'in addition' for the 12 terminal congruent regions (compare with Fig 12). These are indeed unnecessary synonyms for regions already identified in the input. However, the relative *number* of additional labels generated per input region is telling. This number will be highest for regions whose differential placements are the primary drivers of incongruence. As explained above, these are: 2015./2014.{Phoenicopterimorphae, Charadriiformes, Columbimorphae}, respectively with 14, 8, and 7 additional labels. Six redundant split-concept resolution labels are further produced for input regions that are unique to one phylogeny; e.g., 2014.Columbea is also labeled 2015.Neoaves_Clade1 \ 2014.Passerea (where the "\" means: not).

**Table 4:** Overview of 15 newly inferred split-concept resolution regions and labels (or label clusters) for the neoavian conflict region that lack appropriate input clade concept labels. See also Fig 13. For each split-concept resolution label (or label cluster), we provide the two immediate children or constituent concepts 1 and 2 –i.e., what is jointly subsumed 'underneath' the split–as well as the set of lower-level concept regions (using whole-concept resolution labels) that are differentially distributed by the split between the two source phylogenies. * = Two children listed.

#	Split-concept label(s)	Constituent clade concept 1	Constituent clade concept 2	Lower-level concept regions differently assigned by the split
**1**	2015.Neoaves_Clade1 * 2014.Passarea_Clade4	2015.Neoaves_Clade2	2014.Columbea	2015./2014.Otidimorphae, 2015.Strisores/2014.Caprimulgimorphae
**2**	2015.Neoaves_Clade1 * 2014.Passerea	2015./2014.Otidimorphae	2014.Passerea_Clade1	2015.Strisores/2014.Caprimulgimorphae, 2014.Columbea
**3**	2014.Passerea \ 2015.Columbaves	2015.Strisores/2014.Caprimulgimorphae	2014.Passerea_Clade1	2015./2014.Columbimorphae, 2015./2014.Otidimorphae
**4**	2014.Passerea \ 2015.Aequorlitornithes_Clade1	2015.Aequorlitornithes \ 2014.Passerea	2014.Passerea_Clade1 \ 2015.Aequorlitornithes_Clade1	2015./2014.Charadriiformes, 2015./2014.Phoenicopterimorphae
**5**	2014.Passerea \ 2015.Aequorlitornithes	2014.Passera \ 2015.Neoaves_Clade3	2014.Passerea_Clade1 \ 2015.Aequorlitornithes	2015./2014.Ardeae, 2015./2014.Charadriiformes, 2015./2014.Phoenicopterimorphae
**6**	2015.Neoaves_Clade3 \ 2014.Cursorimorphae	2015.Inopinaves	2015.Neoaves_Clade3 \ 2014.Passerea_Clade3	2015./2014.Charadriiformes, 2015./2014.Gruiformes
**7**	2014.Passerea \ 2015.Neoaves_Clade3	2015./2014.Gruiformes	2014.Passerea_Clade4	2015./2014.Ardeae, 2015./2014.Charadriiformes, 2015./2014.Opisthocomiformes, 2015./2014.Phoenicopterimorphae, 2015./2014.Telluraves
**8**	2014.Passerea_Clade1 \ 2015.Aequorlitornithes_Clade1	2014.Passerea_Clade1 \ 2015. Aequorlitornithes	2014.Passerea_Clade2	2015./2014.Charadriiformes, 2015./2014.Phoenicopterimorphae
**9**	{2015.Neoaves_Clade3 \ 2014.Columbea, 2015.Neoaves_Clade3 * 2014.Passerea, 2015.Neoaves_Clade3 * 2014.Passerea_Clade1}	{2015.Aequorlitornithes \ 2014.Columbea, 2015.Aequorlitornithes * 2014.Passerea, 2015.Aequorlitornithes * 2014.Passerea_Clade1}, 2015.Neoaves_Clade3 * 2014.Passerea_Clade3 *	2015.Inopinaves, 2014.Passerea_Clade2 *	2015./2014.Columbimorphae, 2015./2014.Gruiformes, 2015./2014.Otidimorphae, 2015./2014.Phoenicopterimorphae, 2015.Strisores/2014.Caprimulgimorphae
**10**	2015.Neoaves_Clade3 \ 2014.Passerea_Clade3	{2015.Aequorlitornithes \ 2014.Cursorimorphae, 2015.Aequorlitornithes \ 2014.Passerea_Clade3}	2014.Passerea_Clade2	2015./2014.Ardeae, 2015./2014.Charadriiformes, 2015./2014.Gruiformes, 2015./2014.Opisthocomiformes, 2015./2014.Phoenicopterimorphae, 2015./2014.Telluraves
**11**	2014.Passerea_Clade1 \ 2015.Aequorlitornithes	2014.Passerea_Clade3 \ 2015.Aequorlitornithes, 2014.Passerea_Clade3 \ 2015.Aequorlitornithes_Clade1}	2015.Inopinaves	2015./2014.Ardeae, 2015./2014.Charadriiformes, 2015./2014.Gruiformes, 2015./2014.Opisthocomiformes, 2015./2014.Phoenicopterimorphae, 2015./2014.Telluraves
**12**	{2015.Aequorlitornithes \ 2014.Cursorimorphae, 2015.Aequorlitornithes \ 2014.Passerea_Clade3}	2015./2014.Phoenicopterimorphae	2015./2014.Ardeae	2015./2014.Gruiformes, 2015./2014.Opisthocomiformes
**13**	{2015.Aequorlitornithes \ 2014.Columbea, 2015.Aequorlitornithes * 2014.Passerea, 2015.Aequorlitornithes * 2014.Passerea_Clade1}	2015./2014.Ardeae	2015./2014.Charadriiformes	2015./2014.Columbimorphae, 2015./2014.Gruiformes, 2015./2014.Opisthocomiformes, 2015./2014.Otidimorphae, 2015./2014.Phoenicopterimorphae, 2015.Strisores/2014.Caprimulgimorphae, 2015./2014.Telluraves
**14**	2015.Neoaves_Clade3 * 2014.Passerea_Clade3	2015./2014.Charadriiformes	2015./2014.Opisthocomiformes	2015./2014.Ardeae, 2015./2014.Gruiformes, 2015./2014.Phoenicopterimorphae, 2015./2014.Telluraves
**15**	{2014.Passerea_Clade3 \ 2015.Aequorlitornithes, 2014.Passerea_Clade3 \ 2015.Aequorlitornithes_Clade1}	2015./2014.Gruiformes	2015./2014.Opisthocomiformes	2015./2014.Ardeae, 2015./2014.Charadriiformes, 2015./2014.Phoenicopterimorphae

The remaining 21 split-concept resolution labels identify 15 salmon-colored alignment regions– 11 uniquely and 4 redundantly with 2–3 labels each–for which there are *no* suitable labels in either of the phylogenomic input trees (Table 4). Forty-six additional articulations are inferred to align these regions to those displayed in Fig 12. Although these novel regions are not congruent with any clade concepts recognized by the source phylogenies, they are needed to express how exactly the authors' respective clade concepts overlap.

Three distinct reference services are gained by generating the split-concept resolution labels. First, in cases where no whole-concept resolution labels are available, we obtain appropriately short and consistent labels to identify the split regions caused by overlapping clade concepts. Second, the {A*B, A\b, B\a} triplets have an explanatory function, by using the same syntactic set of input labels (A, B) to divide complementary alignment subregions of an overlap. If we focus on one label of a triplet, we can find the two complements, and thereby systematically explore the 'reach' of each split in the alignment. Third, the clade concept labels (A, B) used in the split-concept resolution labels will be exactly those that identify overlapping regions across the source phylogenies.

### Analysis of clade *name* performance

We can now also ask to what extent the clade names (syntax) used by the two author teams succeed or fail to identify congruent and incongruent concept regions (semantics). Such name:meaning (read: "name-to-meaning") analyses were carried out in three previous alignment use cases, with rather unfavorable outcomes for the respective names in use [14, 32, 51]. Here, based on the alignment of Fig 11, the 97 x 83 input concepts yield a set of 8,051 MIR (S16 File). Of these, 384 MIR involve one of four "no coverage" regions added to 2014.JEA concepts. We therefore restrict the name:meaning analysis to the remaining 7,667 MIR (Table 5).

**Table 5:** Clade name-to-RCC–5 relationship reliability analysis for the higher-level neoavian explosion alignment. Relationship data are derived from the set of MIR corresponding to Fig 11 and the S16 File.

Relationship (RCC–5 / clade name)	= =	>	<	><	!	Totals
**Same clade name**	62	0	1	1	0	**64**
**Different clade names**	7	625	691	27	6,253	**7,603**
**Totals**	**69**	**625**	**692**	**28**	**6,253**	**7,667**

Interestingly, the clades names used by the respective author teams fare rather well. Only nine of 7,667 pairings in the MIR (0.12%) are unreliable as identifiers of in-/congruence of the respective RCC–5 articulation. In seven instances, two congruent concepts have different names. Four of these merely involve changes in name endings, viz.: 2015.Accipitriformes = = 2014.Accipitrimorphae, 2015.Galloanserae = = 2014.Galloanseres, 2015.Gaviiformes = = 2014.Gaviimorphae, and 2015.Pteroclidiformes = = 2014.Pterocliformes. The other three instances involve the respectively preferred roots 2015.Strisores and 2014.Caprimulgi-{formes, morphae}. The articulation 2015.Pelecaniformes < 2014.Pelecaniformes is the single instance in which the meaning of the same name is less inclusive in one source (Fig 7). Lastly, the overlapping relationship 2015.Otidimorphae_Clade1 >< 2014.Otidimorphae_Clade1 involves the same name (Figs 12 and 13), though it is not actually used by the author teams (see Methods).

In summary, the clade concept names used by 2015.PEA and 2014.JEA rarely provide an *incorrect* signal regarding in-/congruence. This desirable outcome seems to reflect their recognition that newly inferred clade concepts merit the use of unique names.

### Comparison with other conflict visualizations

We now compare these results with conflict analysis and visualization tools created for the Open Tree of Life project (OToL)–a community-curated tree synthesis platform [13, 22, 23, 24]. The OToL approach is explained in [11, 15, 23, 52, 53]. The method starts off with 'normalizing' all terminal names in the source trees to a common taxonomy [24]. Having the same terminal name means taxonomic concept congruence (= =). To assess conflict from the perspective of one rooted input tree (A), a source edge *j* of that tree is taken to define a rooted bipartition *S*(*j*) = *S*_in_ | *S*_out_, where *S*_in_ and *S*_out_ are the tip sets of the ingroup and outgroup, respectively. The algorithm progresses sectionally from the leaves to the root. Concordance or conflict for a given edge *j* in tree A with that of tree B is a function of the relative overlap of the corresponding tip sets, as follows [23]. Concordance between two edges in the input trees A and B is obtained when B_in_ is a proper subset (⊂) of A_in_ *and* B_out_ ⊂ A_out_. On the other hand, two edges in trees A and B are conflicting if *none* of these sets are empty: A_in_ intersects (⋂) with B_in_, A_in_ ⋂ B_out_, or B_in_ ⋂ A_out_. In other words, conflict means that there is reciprocal overlap in the ingroup and outgroup bipartitions across the two trees.

**Fig 14:**
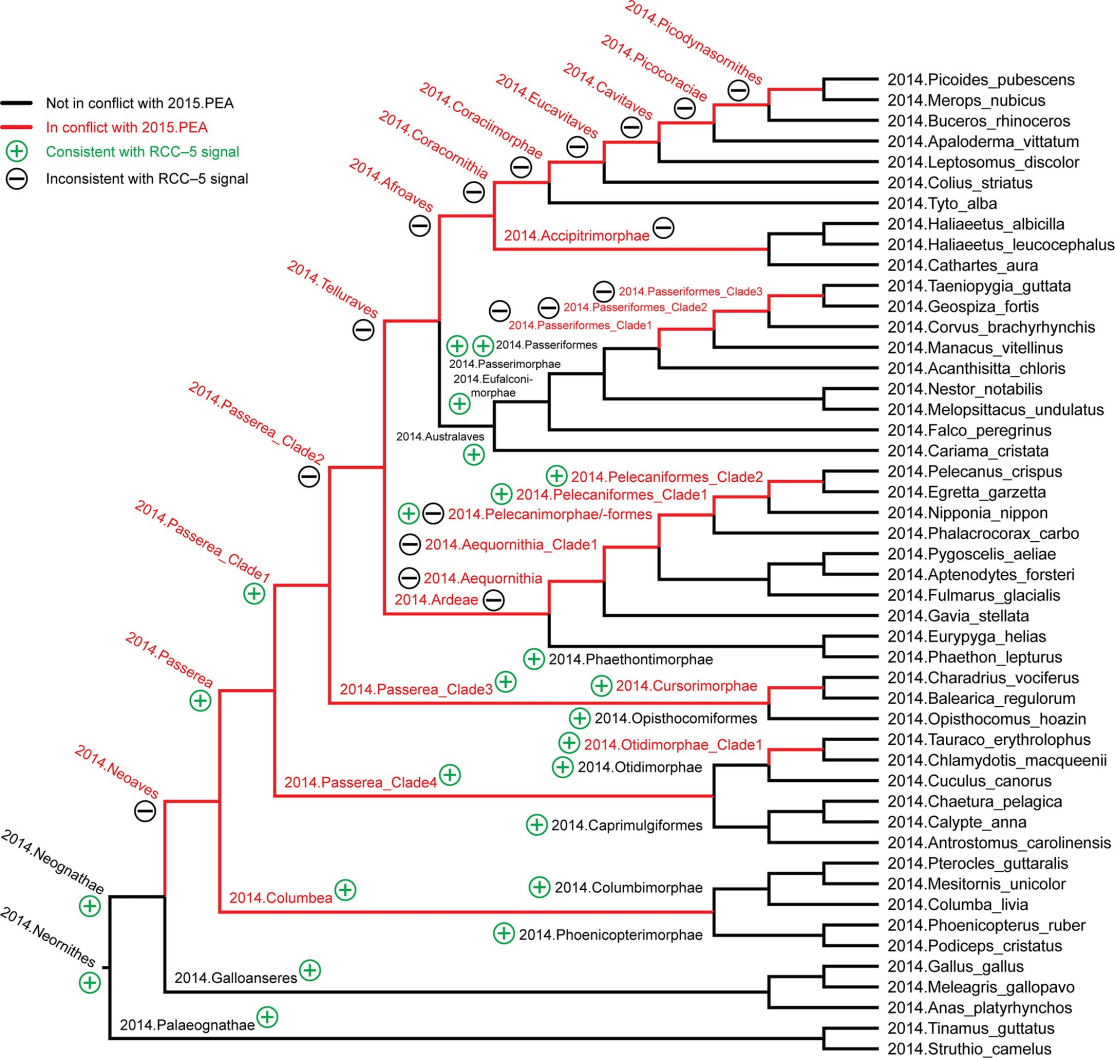
Conflict visualization for Avian phylogenomic relationships, using the method of [11, 15, 23], with 2014.JEA as the primary source phylogeny and 2015.PEA as the alternative. Black edges indicate concordance, whereas red edges signal conflict. Clade and terminal concept labels are added in accordance with the present study. Moreover, consistency or inconsistency of the edge concordance/conflict analysis with the RCC–5 alignments (Figs 7 to 13) are signaled via a green "+" circle and a black "–" circle, respectively. See also S19 File.

**Fig 15:**
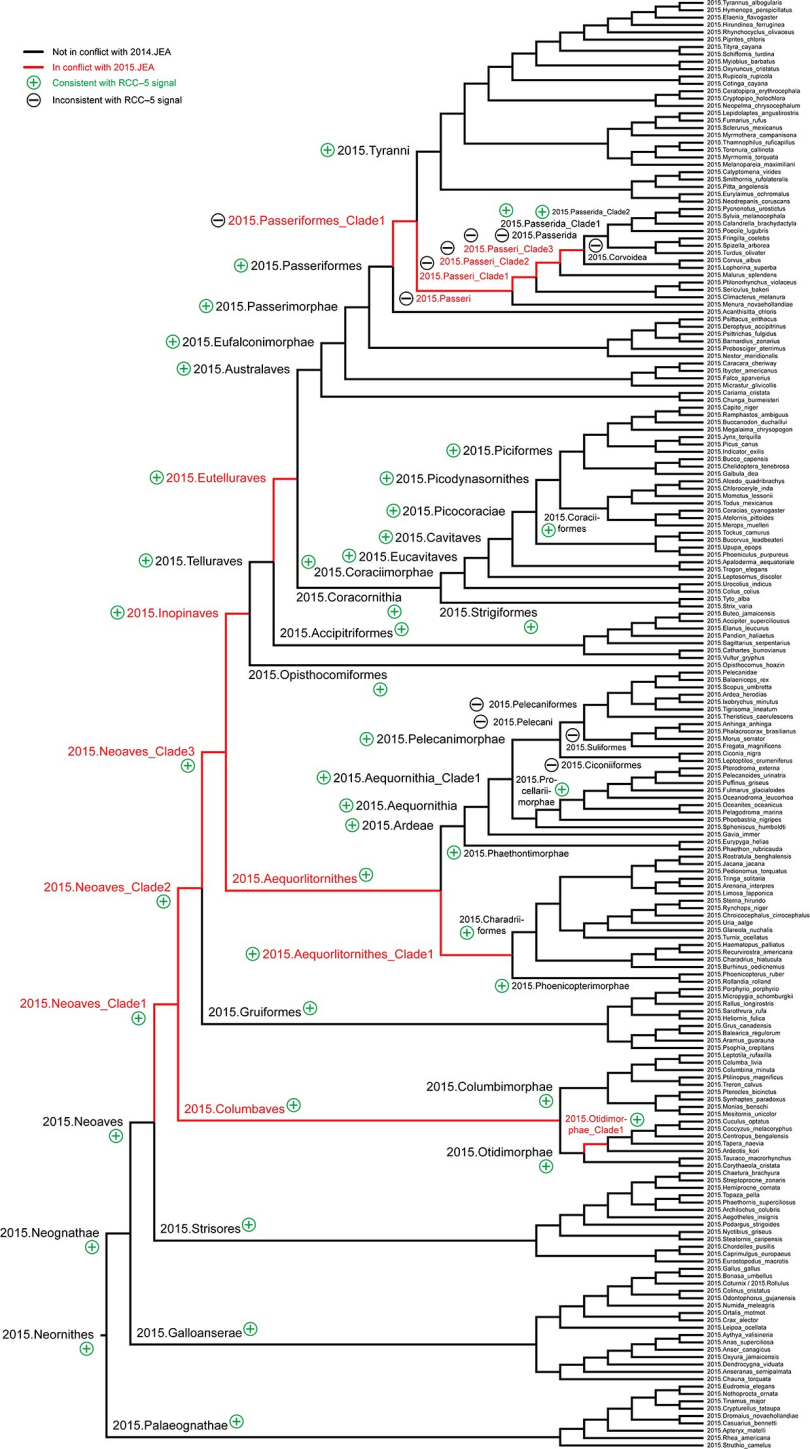
Conflict visualization for Avian phylogenomic relationships, using the method of [11, 15, 23], with 2015.PEA as the primary source phylogeny and 2014.JEA as the alternative. Display conventions as in Fig 14. See also S20 File.

We applied this approach in both directions, i.e. starting with 2014.JEA as primary source and identifying edges therein that conflict with those of 2015.PEA, and vice-versa. The visualizations are shown in Figs 14 and 15, respectively.

Most of the red edges in Fig 15, which is based on the more densely sampled tree sec. 2015.PEA, are consistent with the overlapping RCC–5 relationships shown in Figs 7 to 13. However, within the 2015.Pelicanimorphae, certain RCC–5 overlaps (Fig 7) are not recovered ("false positives"). In addition, numerous edges within the 2015.Passeriformes are shown as conflicting ("false negatives") but are congruent refinements based on the RCC–5 alignment (Fig 8).

Using the less densely sampled tree sec. 2014.JEA as the base topology creates is instructive (Fig 14). Here, a much larger subset of the topology 'backbone' is inferred by the OToL algorithm as conflicting–an outcome that would appear inconsistent. For instance, 2014.{Neoaves, Ardeae, Coracornithia} are shown as conflicting edges in Fig 14, when 2015.{Neoaves, Ardeae, Coracornithia} are concordant edges in Fig 15. The inconsistencies are caused by the addition of terminals sec. 2015.PEA that have no matches in 2014.JEA's sampled tips and tree, and will therefore attach as children to a higher-level parent in the OToL taxonomy. The latter is used to place terminals that are differentially sampled between sources. For instance, 2015.Ciconiiformes–which has no close match in 2014.JEA–may end up attaching as a child of 2014.Neognathae instead of 2014.Pelecanimorphae (Fig 7). Hence the OToL *taxonomy* is used to represent concept intensionality, but it cannot do so reliably if it lacks relevant input concepts. At the time of analysis, the OToL taxonomy lacked a name/concept for "Neoaves". This means that the 2015./2014.Neoaves ingroup/outgroup bipartitions will be inconsistent in evaluating the placement of 2015.Ciconiiformes, showing conflict in Fig 14 but not in Fig 15.

## Discussion

### Key phylogenomic conflict representation conventions

We review the key conventions of our approach before discussing services that can be derived from our alignments.

1. Using the taxonomic concept label convention of [14] allows us to individuate each concept entailed in 2014.JEA and 2015.PEA, even if the taxonomic or clade concept *name* components are identical, as in 2015.Pelicaniformes < 2014.Pelicaniformes.
2. Because our main intention is to represent phylogenomic congruence and conflict across these inferred phylogenies, there is no need to speak of sameness in any profound sense, such as referring to the "same {clades, nodes, species, taxa}". Such language is best used once we shift from modeling similarities and differences between human-made phylogenomic theories, to hopefully (but not necessarily) robust evolutionary inferences. We thereby avoid blurring the lines between two important communication goals best met by maintaining complementary manners of speaking [21].
3. Linking concepts via *is_a* (parent/child) relationships permits the assembly of single-source hierarchies, whereas RCC–5 articulations express the relative congruence of concept regions across multi-source hierarchies. Uncertainty can be accommodated via disjunctions of the base five relations [33].
4. Under parent coverage, differential child-level sampling will propagate up to yield incongruent relationships among parent-level clade concepts [14, 26, 29]. Local relaxation of the coverage constraint can mitigate this effect. However, this requires expert judgment [30], reflected in input articulations that stipulate counter-factual circumstances. We can thereby indirectly model *intensional* (property-based) node concept definitions in RCC–5, and obtain instances of clade concept congruence in spite of incongruent terminal sampling (Figs 1–4).
5. Because every clade concept region to be aligned requires a label suited for human communication, we need to supply such labels when the sources fail to do so. A pragmatic solution is to utilize the next available higher-level name and add the suffix "_Clade#", as in 2015.Neoaves_Clade1 or 2014.Passerea_Clade3 (Tables 1 and 2). This may involve deciding on a breadth- vs. depth-first approach, and having a rule to prioritize between sibling nodes.
6. In some instances, the source may provide a clade concept label for a non-monophyletic tree region. Representing such mismatches is achieved by providing an(other) alignment between (1) the reference classification and (2) the phylogeny to which the labels are incongruently applied (Figs 5 and 6).
7. Multi-rooted, bottom-up, and incremental partitions may be required to manage the logic reasoning complexity of large or even global alignments [28]. Consistent alignments of higher-level concept hierarchies (Fig 11), can be derived from this bottom-up approach by propagating the inferred parent-level articulations while pruning the children used for aligning lower-level partitions [14, 28, 51].
8. Overlapping relationships among higher-level clade concepts can be represented using either whole-concept or split concept resolution (compare Figs 9 and 10; Figs 12 and 13). The latter option provides a uniquely powerful syntax to partition and label the alignment regions created by concept overlap (Tables 3 and 4).
9. The reasoner-inferred MIR are useful for quantifying all pairwise instances where the *names* used by each source succeed, or fail, in matching the signal of the RCC–5 relationships (Table 5).
10. The alignments can be compared with other conflict representation methods, such as the OToL concordance/conflict visualizations [11, 15, 23]. This is particularly illustrative in cases where differential sampling of low-level concepts generates unequal assessments between the OToL and RCC–5 approaches (Figs 14 and 15). The latter is more reliable in cases where expert judgment is needed to represent higher-level concept intensionality under strongly divergent sampling schemes.

### Knowledge representation services

What can we gain from this approach, both narrowly for this use case and for future data integration in systematics?

Data representation designs have inherent trade-offs. Unlike other semi-/automated phylogenomic conflict visualization methods [13, 23, 24], the above approach requires extensive *upfront* application of human expertise to obtain the intended outcomes. In return, the RCC–5 alignments deliver a level of explicitness and verbal precision exceeding that of published alternatives [4, 5, 6, 9, 16, 17]. We can not just verbalize all instances of congruence and conflict, but transparently document and therefore understand their provenance in a global alignment (Figs 11 and 13). In other words, the RCC–5 alignments provide a logically tractable means to identify and also *explain* the extent of conflict.

We can derive novel data services from the alignment products. (Note that these services are *envisioned* but not yet implemented in a web-based platform.) Example queries include the following. (1) Show all congruent regions of the alignment and their clade concept labels. (2) Modify this query to only apply to alignment regions subsumed under one particular concept and source, such as 2014.Columbea. (3) For any subset region of the global alignment (e.g., 2015./2014.Australaves), show the lowest-level pairs of children that are sampled congruently, versus those that are sampled incongruently. (4) Highlight within such an alignment region all clade concepts for which parent coverage is relaxed, and which show congruence as a result of this action. (5) Highlight sets of concepts where incongruence is due to differential granularity (sampling), versus actual overlap. (6) Identify and rank concepts that participate in the greatest number of overlapping relationships (Table 3). (7) Identify and rank the longest chains of nested, overlapping concept sets (Fig 12). (8) Highlight the congruent, lowest-level concept pairs whose incongruent placement into higher-level regions causes the chains of overlap. (9) List all split-concept resolution labels in complementary triplets {A*B, A\b, B\a}, and provide for each the two immediate children and (again) the set of lower-level, whole-concept resolution regions that are differentially distributed by the split (Fig 13 and Table 4). (10) Identify clade names that are unreliable across the source phylogenies; including identical clade name pairs that participate in concept labels with an incongruent relationship, or different clade names whose concept labels have a congruent relationship (Table 5).

All of the above queries, and many others we could propose, are enabled by our RCC–5 representation and reasoning conventions, which therefore present a new foundation for building logic-based, machine-scalable data integration services for the age of phylogenomics. Conceptualizing node identity and congruence this way addresses a gap in current systematic theory that is not adequately filled by other syntactic solutions.

**Linnaean naming.** We have shown elsewhere that homonymy and synonymy relationships are unreliable indicators of congruence [14, 26, 32]. Code-enforced Linnaean naming is designed to fixate the meaning of names by ostension, while allowing the intensional components to remain ambiguous [21, 54, 55, 56, 57]. This trade-off effectively shifts the burden of disambiguating varying intensionalities associated with Linnaean names onto an additional, interpreting agent–typically human experts. Our RCC–5 alignment approach can be viewed as a way to formalize the disambiguation effort, so that it can attain machine-interpretability.

**Phyloreferencing.** Similarly, node-based *phyloreferences* [58, 59, 60] are not well suited to reconstruct an alignment such as that of 2015./2014.Pelecanimorphae (Fig 7). This would require: (1) an elaborate notion of phyloreference *homonymy* and *synonymy* (e.g., 2015.Pelecanifores versus 2014.Pelecaniformes, or 2015.Strisores versus 2014.Caprimulgimorphae); (2) node-based definitions with inclusion/exclusion constraints that cover all terminals in the phylogeny; and (3) synapomorphy-based definitions at higher levels to model the local relaxation of coverage constraints. All of these functions may be feasible in principle with phyloreferences, provided that human experts are permitted to enact them. However, it may be fair to say that phyloreferences were not mainly designed to bring out fine differences between node concepts across multiple phylogenies. They are best utilized when concept evolution and conflict are not the main drivers of an information system design.

### Role of trained judgment

The two largest alignments of 2015./2014.Neornithes (without) / 2015./2014.Telluraves jointly entail 895 concepts and 95 instances of relaxed parent coverage. They provide us with 97 congruent regions in the global alignment, of which 85 regions are obtained only because of the indirect modeling of intensional node definitions. The contingency of the alignment outcome on expert intentions is neither surprising nor trivial. We should therefore explore this dependency more deeply.

Redelings and Holder [23: pp. 5–6] comment on the OToL synthesis method: "Any approach to supertree construction must deal with the need to adjudicate between conflicting input trees. We choose to deal with conflict by ranking the input trees, and preferring to include edges from higher-ranked trees. The merits of using tree ranking are questionable because the system does not mediate conflicts based on the relative amount of evidence for each alternative. […] In order to produce a comprehensive supertree, we also require a rooted taxonomy tree in addition to the ranked list of rooted input trees. Unlike other input trees, the taxonomy tree is required to contain all taxa, and thus has the maximal leaf set. We make the taxonomy tree the lowest ranked tree. […] Our method must resolve conflicts in order to construct a single supertree. However, the rank information used to resolve conflicts is an input to the method, not an output from the method. We thus perform curation-based conflict resolution, not inference-based conflict resolution."

Clearly, the outcomes of the OToL synthesis method are also deeply dependent on expert input regarding the relative ranking of input phylogenies and of the OToL taxonomy [24]. We have shown (Figs 14 and 15) that these choices can lead to inconsistent outcomes whenever the sequence of input trees determines how concordance and conflict are negotiated by the algorithms. If the less densely sampled tree is prioritized, and the taxonomy cannot accommodate all components of a lower-ranked tree, then the method will show more conflict in comparison to an inverse input sequence. Any global rule of priority among trees is a poor proxy for modeling individual node concept intensionality, which requires making reliable, local decisions between (1) conflict due to differential granularity versus (2) conflict due to overlap.

We can now return to the challenge posed in the Introduction. How do we build a data service for phylogenomic knowledge in the face of persistent conflict? Our answer is novel in the following sense. Assuming that such a service is desirable, we show that achieving it fundamentally depends on making and expressing upfront empirical commitments about the intensionalities of clade concepts whose children are incongruently sampled. Without embedding these judgments into the alignment input, we lose the 85 congruent parent regions recovered under relaxed parent coverage. We furthermore lose the ability to distinguish the former from more than 340 alignment regions that are *not* congruent. And we lose the power to express the nature of this residual conflict–granularity versus overlaps–and how to resolve it.

In other words, the first step for building the phylogenomic data knowledge service will be to recognize that conceptualizations of node identity within such a system just cannot be provided through some mechanical, 'objective' criterion. Instead, we need an inclusive standard of objectivity that embraces trained judgment as an integral part of identifying and linking node concepts [30]. In that sense, phylogenomic syntheses *are* inference-based (*contra* [23]) and also driven by a specific purpose. As integrative biologists, our goal in providing RCC–5 alignments is to maximize intensional node congruence. There may not be a more reliable criterion for achieving this than expert judgment, which draws on complex and context-specific theoretical knowledge [40, 43, 61]. Logic representation and reasoning can help render these constraints explicit and consistent, and expose implicit articulations through the MIR which encompass all node concepts in an alignment. But logic cannot substitute the expert aligners' intensional aims and definitions.

Building a phylogenomic data knowledge service forces us to become experts about externally generated results that *conflict* with those which we may (currently) publish or endorse. We need to become experts of another author team's node concepts, to the point where we are comfortable with expressing counter-factual statements regarding their intensionalities, in spite of incongruent child sampling. This will require a profound but necessary adjustment in achieving a culture of synthesis in systematics that no longer manages conflict this way: "If we do not agree, then it is either our view over yours, or we just collapse all conflicting node concepts into polytomies". In contrast, we need to develop the following culture of synthesis: "We may not agree with you, but we understand your phylogenomic inference well enough to express our dis-/agreements in a logic-compatible syntax. Therefore, we are prepared to assert and refine articulations from our concepts to yours for the purpose of maximizing intensional node congruence". Only then can we expect to also maximize the empirical translatability of biological data linked to diverging phylogenomic hypotheses.

Shifting towards the latter attitude will be more challenging than providing the operational logic to enable scalable alignments. Automation of certain workflow components is certainly possible. Ultimately, the logic or technical issues are not the hardest bottlenecks to overcome. Designers of future data environments capable of verbalizing phylogenomic conflict and synthesis need to reflect on how to promote a culture where experts routinely re-/assess the intensionalities of node concepts published by peers. If we wish to track progress and conflict across phylogenomic inferences, we first need to design a value system that better enables and motivates experts to do so.

### Response to reviewers

He we discuss various reviewer comments that merit a response but would break up the main flow of the narrative if inserted earlier. We take liberty to assign a header to each comment.

**Phylogenetic clade definitions and taxonomic concepts are fundamentally mismatched.** One reviewer pointed out that clade hypotheses are about branching patterns and relationships of descent, and therefore are mismatched with our notion of node intensionality. We disagree in the following sense. We believe that we are not conflating two fundamentally different kinds of clade conceptualizations, as much as bringing out with the RCC–5 alignments one aspect in the dual, or hybrid nature of clade concepts. The latter are not either this or that–with parallels to the taxa as classes-versus-individuals literature–both can be *both*, depending on the pragmatic interest [36, 37, 62]. For the purpose of synthesis and integration, modeling the intensional aspect of clade concepts is critical. We see this purpose reflected (e.g.) in the matching of high-level terminals in [3].

**No mechanism for quantitatively expressing uncertainty about tree topology.** The same reviewer pointed out that we select single point estimate topologies for each author team, thereby not accounting for the complex likelihood surfaces of the reconstructions and the relative uncertainty of each topology. Applied to what we show here, this criticism is valid. However, it would be feasible perform RCC–5 alignments on a cluster of paired topology alternatives with similar likelihood values. The products can be compared in order to manage uncertainty, through identification of stable versus variable regions across multiple alignments. If most of the variation occurs at higher levels, this would mean that the vast majority of our low-level RCC–5 input articulations could be reused.

**Phylogenetic conflict is not limited to two trees.** Another reviewed pointed out the need to align more than two phylogenies in situations where many recent reconstructions are available to inform a synthesis [5, 6, 11]. While the current logic toolkit handles three or more input trees in principle, there certainly are unrealized opportunities to model transitive relationships (example: for concepts A, B, C in the input trees T1, T2, T3: *if* A_T1_ = = B_T2_ *and* B_T2_ = = C_T3_ *then* A_T1_ = = C_T3_). 'Smartly' breaking down alignments of three or more trees while exploiting transitive relationships, as well as visualizing the outcomes accessible ways, are important future improvements for this approach.

**"Not every clade [concept] is worth labeling and discussing".** We can agree with that assessment. But, having a framework to do so is critical to evaluating the *feasibility* of a phylogenomic data knowledge service, and should not trail behind discussions regarding its *desirability*. If we have no formalized means of translating Fig 1 of [3] into a machine-accessible language (Fig 11), then we cannot fully understand the costs and benefits of building the service.

**Incentivizing alignment production.** One reviewer pointed out that efforts to align multiple trees are costly, and inquired about our suggestions for incentivizing such expert contributions. An initial answer would point to the creation of an e-journal, where multi-phylogeny and -taxonomy alignments can be published either as stand-alone articles or in association with separate publications of new tree reconstructions. The platform of a formal journal best responds to expert needs to receive academic credit [63]. Knowledge systems such as [64] could represent the information input and output. The most valuable product of such an e-journal are the expert-vetted sets of RCC–5 articulations, which represent a new kind of "systematic intelligence". Scientists and commercial publishers may utilize this intelligence to improve the precision and recall of systematically structured data [54], where business models would focus on the latter clients for revenue. Needless to say, these are ideas that will take time to concretize and test.

## Supporting information

S1 File **(A)** Reasoner input constraints for the 2015./2014.Psittaciformes alignment, with coverage globally applied. **(B)** Input visualization for the 2015./2014.Psittaciformes alignment, with coverage globally applied.
(ZIP)

S2 File **(A)** Alignment visualization for the 2015./2014.Psittaciformes alignment, with coverage globally applied. **(B)** Set of Maximally Informative Relations (MIR) inferred for the 2015./2014.Psittaciformes alignment, with coverage globally applied. Total = 108 MIR.
(ZIP)

S3 File **(A)** Reasoner input constraints for the 2015./2014.Psittaciformes alignment, with coverage locally relaxed. Includes information on run commands; and 4 instances of "no coverage". **(B)** Input visualization for the 2015./2014.Psittaciformes alignment, with coverage locally relaxed.
(ZIP)

S4 File **(A)** Alignment visualization for the 2015./2014.Psittaciformes alignment, with coverage locally relaxed. **(B)** Set of Maximally Informative Relations (MIR) inferred for the 2015./2014.Psittaciformes alignment, with coverage locally relaxed. Total = 160 MIR.
(ZIP)

S5 File **(A)** Reasoner input constraints for the alignment of passeriform clade concepts ("Phylo2015") sec. 2015.PEA with the corresponding classification concepts ("Class2015") sec. Gill & Donsker (2015); including the (paraphyletic) Class2015.Eurylaimidae. Includes information on run commands; and 0 instances of "no coverage". **(B)** Input visualization for the alignment of passeriform clade concepts ("Phylo2015") sec. 2015.PEA with the corresponding classification concepts ("Class2015") sec. Gill & Donsker (2015); including the (paraphyletic) Class2015.Eurylaimidae.
(ZIP)

S6 File **(A)** Alignment visualization for the alignment of passeriform clade concepts ("Phylo2015") sec. 2015.PEA with the corresponding classification concepts ("Class2015") sec. Gill & Donsker (2015); including the (paraphyletic) Class2015.Eurylaimidae. **(B)** Set of Maximally Informative Relations (MIR) inferred for the alignment of passeriform clade concepts ("Phylo2015") sec. 2015.PEA with the corresponding classification concepts ("Class2015") sec. Gill & Donsker (2015); including the (paraphyletic) Class2015.Eurylaimidae. Total = 63 MIR.
(ZIP)

S7 File Supporting files for the alignment of tyrannoid clade concepts ("Phylo2015") sec. 2015.PEA with the corresponding classification concepts ("Class2015") sec. Gill & Donsker (2015); including the (paraphyletic) Class2015.Tityridae. **(A)** Reasoner input constraints. Includes information on run commands; and 0 instances of "no coverage". **(B)** Input visualization. **(C)** Alignment visualization. **(D)** Set of Maximally Informative Relations (MIR). Total = 140 MIR.
(ZIP)

S8 File **(A)** Reasoner input constraints for the alignment of procellariiform clade concepts ("Phylo2015") sec. 2015.PEA with the corresponding classification concepts ("Class2015") sec. Gill & Donsker (2015); including the (paraphyletic) Class2015.Hydrobatidae and Class2015.Procellariidae. Includes information on run commands; and 0 instances of "no coverage". **(B)** Input visualization for the alignment of procellariiform clade concepts ("Phylo2015") sec. 2015.PEA with the corresponding classification concepts ("Class2015") sec. Gill & Donsker (2015); including the (paraphyletic) Class2015.Hydrobatidae and Class2015.Procellariidae. **(C)** Alignment visualization for the alignment of procellariiform clade concepts ("Phylo2015") sec. 2015.PEA with the corresponding classification concepts ("Class2015") sec. Gill & Donsker (2015); including the (paraphyletic) Class2015.Hydrobatidae and Class2015.Procellariidae. **(D)** Set of Maximally Informative Relations (MIR) inferred for the alignment of procellariiform clade concepts ("Phylo2015") sec. 2015.PEA with the corresponding classification concepts ("Class2015") sec. Gill & Donsker (2015); including the (paraphyletic) Class2015.Hydrobatidae and Class2015.Procellariidae. Total = 221 MIR.
(ZIP)

S9 File **(A)** Reasoner input constraints for the alignment of caprimulgiform clade concepts ("Phylo2015") sec. 2015.PEA with the corresponding classification concepts ("Class2015") sec. Gill & Donsker (2015); including the (paraphyletic) Class2015.Caprimulgiformes. Includes information on run commands; and 0 instances of "no coverage". **(B)** Input visualization for the alignment of caprimulgiform clade concepts ("Phylo2015") sec. 2015.PEA with the corresponding classification concepts ("Class2015") sec. Gill & Donsker (2015); including the (paraphyletic) Class2015.Caprimulgiformes. **(C)** Alignment visualization for the alignment of caprimulgiform clade concepts ("Phylo2015") sec. 2015.PEA with the corresponding classification concepts ("Class2015") sec. Gill & Donsker (2015); including the (paraphyletic) Class2015.Caprimulgiformes. **(D)** Set of Maximally Informative Relations (MIR) inferred for the alignment of caprimulgiform clade concepts ("Phylo2015") sec. 2015.PEA with the corresponding classification concepts ("Class2015") sec. Gill & Donsker (2015); including the (paraphyletic) Class2015.Caprimulgiformes. Total = 672 MIR.
(ZIP)

S10 File **(A)** Reasoner input constraints for the 2015./2014.Neornithes alignment (excepting 2015./2014.Telluraves), with coverage locally relaxed. Includes information on run commands; and 58 instances of "no coverage". **(B)** Input visualization for the 2015./2014.Neornithes alignment (excepting 2015./2014.Telluraves), with coverage locally relaxed. **(C)** Alignment visualization for the 2015./2014.Neornithes alignment (excepting 2015./2014.Telluraves), with coverage locally relaxed. **(D)** Set of Maximally Informative Relations (MIR) inferred for the 2015./2014.Neornithes alignment (excepting 2015./2014.Telluraves), with coverage locally relaxed. Total = 68,208 MIR.
(ZIP)

S11 File **(A)** Reasoner input constraints for the 2015./2014.Telluraves alignment, with coverage locally relaxed. Includes information on run commands; and 37 instances of "no coverage". **(B)** Input visualization for the 2015./2014.Telluraves alignment, with coverage locally relaxed. **(C)** Alignment visualization for the 2015./2014.Telluraves alignment, with coverage locally relaxed. **(D)** Set of Maximally Informative Relations (MIR) inferred for the 2015./2014.Telluraves alignment, with coverage locally relaxed. Total = 32,864 MIR.
(ZIP)

S12 File **(A)** Reasoner input constraints for the 2015./2014.Pelecanimorphae alignment, with coverage locally relaxed. Includes information on run commands; and 2 instances of "no coverage". **(B)** Input visualization for the 2015./2014.Pelecanimorphae alignment, with coverage locally relaxed. **(C)** Alignment visualization for the 2015./2014.Pelecanimorphae alignment, with coverage locally relaxed. **(D)** Set of Maximally Informative Relations (MIR) inferred for the 2015./2014.Pelecanimorphae alignment, with coverage locally relaxed. Total = 200 MIR.
(ZIP)

S13 File **(A)** Reasoner input constraints for the 2015.Passeri/2014.Passeriformes_Clade2 alignment, with coverage locally relaxed. Includes information on run commands; and 1 instance of "no coverage". **(B)** Input visualization for the 2015.Passeri/2014.Passeriformes_Clade2 alignment, with coverage locally relaxed. **(C)** Alignment visualization for the 2015.Passeri/2014.Passeriformes_Clade2 alignment, with coverage locally relaxed. **(D)** Set of Maximally Informative Relations (MIR) inferred for the 2015.Passeri/2014.Passeriformes_Clade2 alignment, with coverage locally relaxed. Total = 140 MIR.
(ZIP)

S14 File **(A)** Reasoner input constraints for the 2015./2014.Telluraves alignment (higher-level subset), under whole-concept resolution. Includes information on run commands; and 0 instances of "no coverage". **(B)** Input visualization for the 2015./2014.Telluraves alignment (higher-level subset), under whole-concept resolution. **(C)** Alignment visualization for the 2015./2014.Telluraves alignment (higher-level subset), under whole-concept resolution. **(D)** Set of Maximally Informative Relations (MIR) inferred for the 2015./2014.Telluraves alignment (higher-level subset), under whole-concept resolution. Total = 81 MIR.
(ZIP)

S15 File **(A)** Reasoner input constraints for the 2015./2014.Telluraves alignment (higher-level subset), under split-concept resolution. Includes information on run commands; and 0 instances of "no coverage". **(B)** Input visualization for the 2015./2014.Telluraves alignment (higher-level subset), under split-concept resolution. **(C)** Alignment visualization for the 2015./2014.Telluraves alignment (higher-level subset), under split-concept resolution. **(D)** Set of Maximally Informative Relations (MIR) inferred for the 2015./2014.Telluraves alignment (higher-level subset), under split-concept resolution. Total = 81 MIR.
(ZIP)

S16 File **(A)** Reasoner input constraints for the 2015./2014.Neornithes alignment, under whole-concept resolution, ranging from the root to the ordinal level (with exceptions where needed). Includes information on run commands; and 4 instances of "no coverage". **(B)** Input visualization for the 2015./2014.Neornithes alignment, under whole-concept resolution, ranging from the root to the ordinal level (with exceptions where needed). **(C)** Alignment visualization for the 2015./2014.Neornithes alignment, under whole-concept resolution, ranging from the root to the ordinal level (with exceptions where needed). **(D)** Set of Maximally Informative Relations (MIR) inferred for the 2015./2014.Neornithes alignment, under whole-concept resolution, ranging from the root to the ordinal level (with exceptions where needed). Total = 8,051 MIR.
(ZIP)

S17 File **(A)** Reasoner input constraints for the 2015./2014.Neoaves alignment, under whole-concept resolution, limited to the main conflict region. Includes information on run commands; and 0 instances of "no coverage". **(B)** Input visualization for the 2015./2014.Neoaves alignment, under whole-concept resolution, limited to the main conflict region. **(C)** Alignment visualization for the 2015./2014.Neoaves alignment, under whole-concept resolution, limited to the main conflict region. **(D)** Set of Maximally Informative Relations (MIR) inferred for the 2015./2014.Neoaves alignment, under whole-concept resolution, limited to the main conflict region. Total = 441 MIR.
(ZIP)

S18 File **(A)** Reasoner input constraints for the 2015./2014.Neoaves alignment, under split-concept resolution, limited to the main conflict region. Includes information on run commands; and 0 instances of "no coverage". **(B)** Input visualization for the 2015./2014.Neoaves alignment, under split-concept resolution, limited to the main conflict region. **(C)** Alignment visualization for the 2015./2014.Neoaves alignment, under split-concept resolution, limited to the main conflict region. **(D)** Set of Maximally Informative Relations (MIR) inferred for the 2015./2014.Neoaves alignment, under split-concept resolution, limited to the main conflict region. Total = 441 MIR.
(ZIP)

S19 File Output tree file (visualized using FigTree) of the OToL conflict visualization method, with 2014.JEA as the primary source phylogeny and 2015.PEA as the alternative. (TRE)

S20 File Output tree file (visualized using FigTree) of the OToL conflict visualization method, with 2015.PEA as the primary source phylogeny and 2014.JEA as the alternative. (TRE)
