# Supplementary figures and images for: Verbalizing phylogenomic conflict: Representation of node congruence across competing reconstructions of the neoavian explosion
Source: PLoS Comput Biol. 2019 Feb 15;15(2):e1006493. doi: 10.1371/journal.pcbi.1006493 (PMC6395011; doi:10.1371/journal.pcbi.1006493)

| Nodes         |    |
|---------------|----|
| 2015          | 18 |
| 2014          | 6  |
| Edges         |    |
| is_a (2015)   | 17 |
| is_a (2014)   | 5  |
| articulations | 8  |

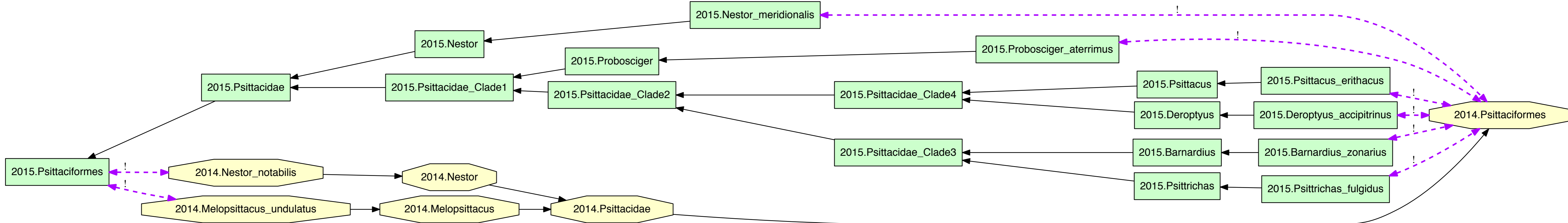

Supplement: S1 File — (A) Reasoner input constraints for the 2015./2014.Psittaciformes alignment, with coverage globally applied. (B) Input visualization for the 2015./2014.Psittaciformes alignment, with coverage globally applied. (ZIP) [file pcbi.1006493.s001.zip › S1B-Psittaciformes-Coverage-Applied.pdf]

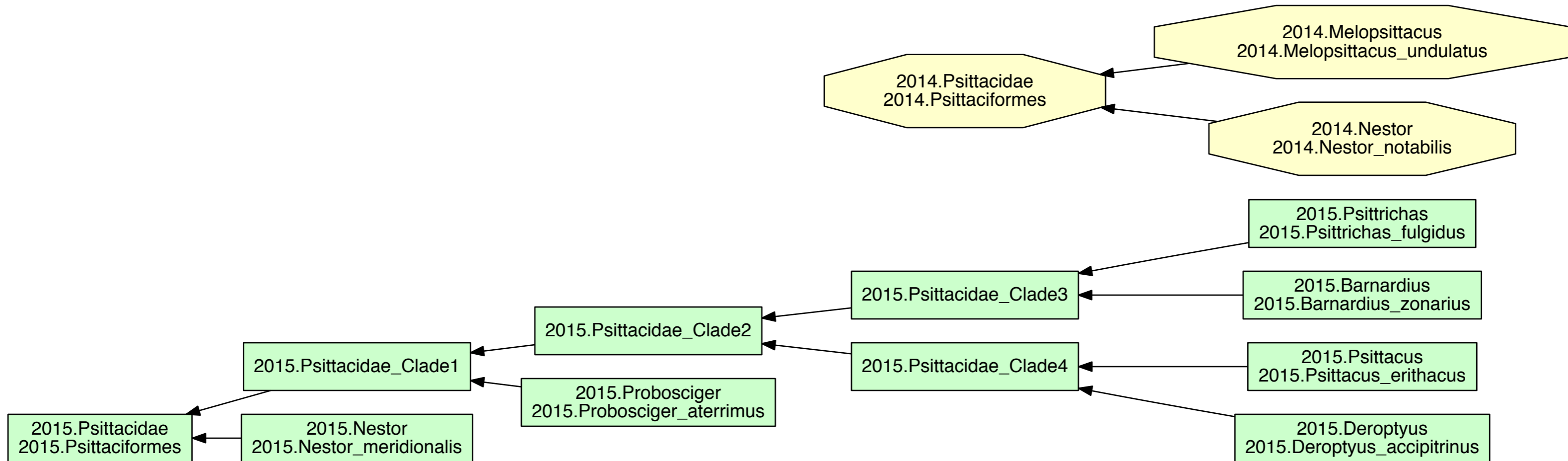

| Nodes        |    |
|--------------|----|
| 2015         | 11 |
| 2014         | 3  |
| Edges        |    |
| is_a (input) | 12 |

Supplement: S2 File — (A) Alignment visualization for the 2015./2014.Psittaciformes alignment, with coverage globally applied. (B) Set of Maximally Informative Relations (MIR) inferred for the 2015./2014.Psittaciformes alignment, with coverage globally applied. Total = 108 MIR. (ZIP) [file pcbi.1006493.s002.zip › S2A-Psittaciformes-Coverage-Applied-0-mnpw.pdf]

| Nodes         |    |
|---------------|----|
| 2015          | 20 |
| 2014          | 8  |
| Edges         |    |
| is_a (2015)   | 19 |
| is_a (2014)   | 7  |
| articulations | 19 |

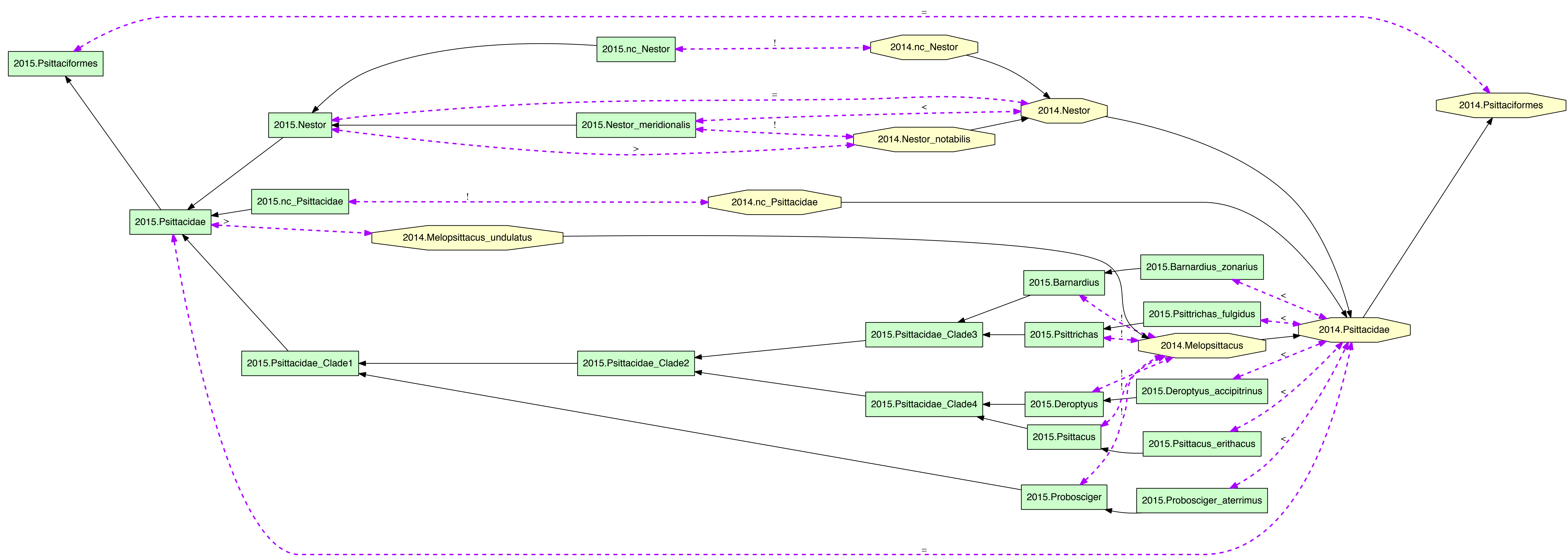

Supplement: S3 File — (A) Reasoner input constraints for the 2015./2014.Psittaciformes alignment, with coverage locally relaxed. Includes information on run commands; and 4 instances of "no coverage". (B) Input visualization for the 2015./2014.Psittaciformes alignment, with coverage locally relaxed. (ZIP) [file pcbi.1006493.s003.zip › S3B-Psittaciformes-Coverage-Relaxed.pdf]

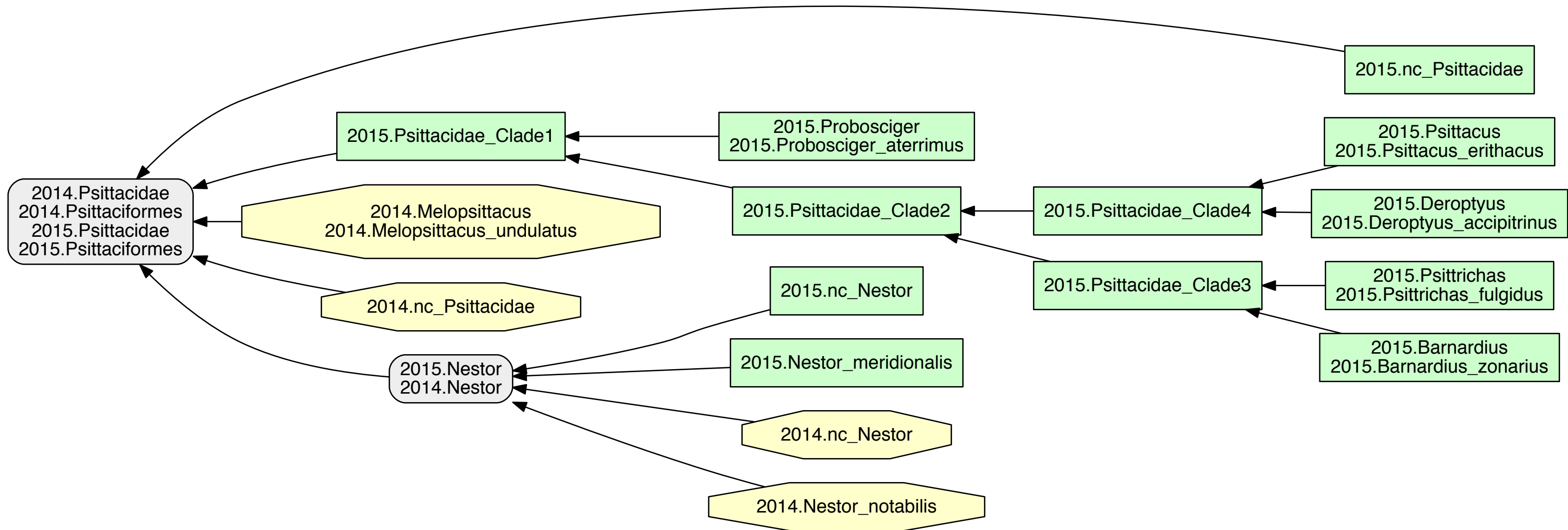

| Nodes        |    |
|--------------|----|
| 2015         | 12 |
| 2014         | 4  |
| congruent    | 2  |
| Edges        |    |
| is_a (input) | 17 |

Supplement: S4 File — (A) Alignment visualization for the 2015./2014.Psittaciformes alignment, with coverage locally relaxed. (B) Set of Maximally Informative Relations (MIR) inferred for the 2015./2014.Psittaciformes alignment, with coverage locally relaxed. Total = 160 MIR. (ZIP) [file pcbi.1006493.s004.zip › S4A-Psittaciformes-Coverage-Relaxed-0-mnpw.pdf]

| Nodes            |   |
|------------------|---|
| Phylo2015        | 9 |
| Class2015        | 7 |
| Edges            |   |
| is_a (Phylo2015) | 8 |
| is_a (Class2015) | 6 |
| articulations    | 6 |

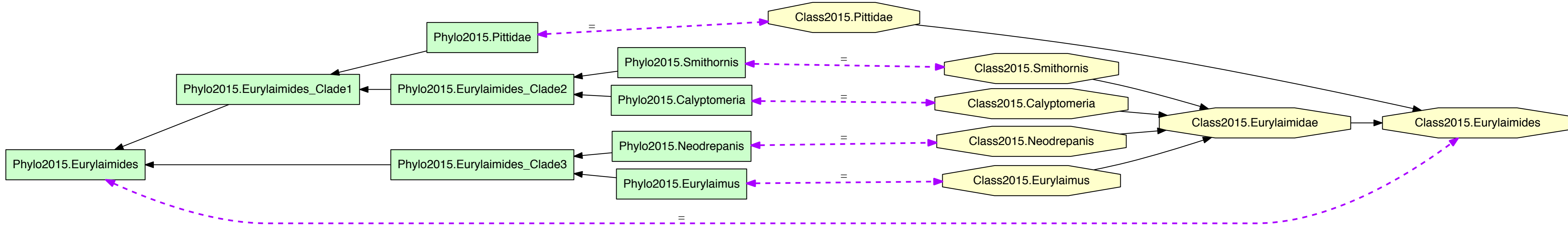

Supplement: S5 File — (A) Reasoner input constraints for the alignment of passeriform clade concepts ("Phylo2015") sec. 2015.PEA with the corresponding classification concepts ("Class2015") sec. Gill & Donsker (2015); including the (paraphyletic) Class2015.Eurylaimidae. Includes information on run commands; and 0 instances of "no coverage". (B) Input visualization for the alignment of passeriform clade concepts ("Phylo2015") sec. 2015.PEA with the corresponding classification concepts ("Class2015") sec. Gill & Donsker (2015); including the (paraphyletic) Class2015.Eurylaimidae. (ZIP) [file pcbi.1006493.s005.zip › S5B-Eurylaimidae-Paraphyletic.pdf]

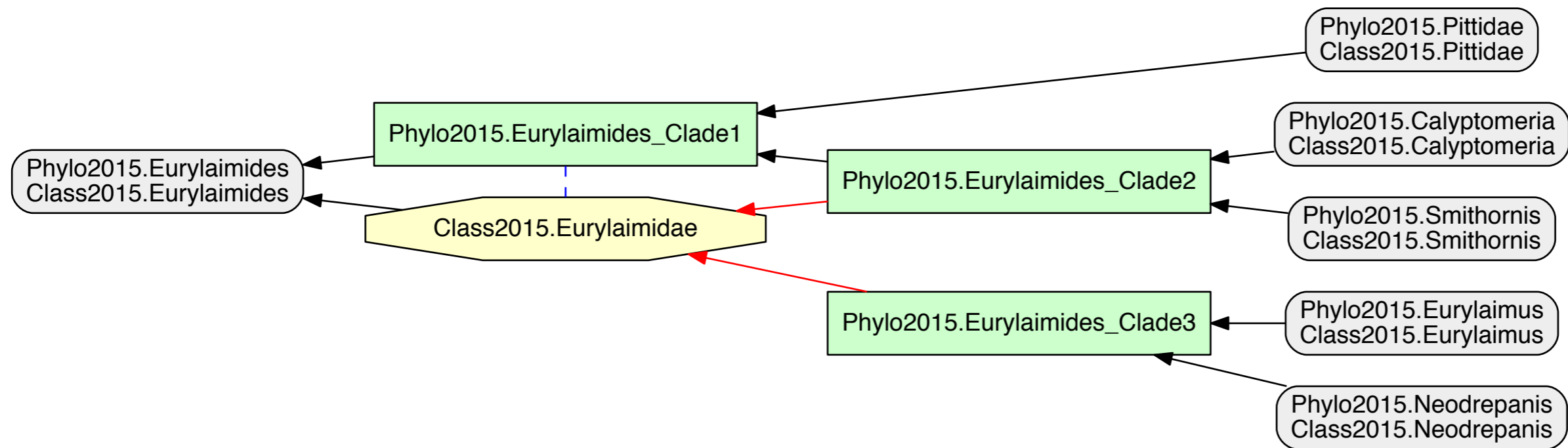

| Nodes           |   |
|-----------------|---|
| congruent       | 6 |
| Phylo2015       | 3 |
| Class2015       | 1 |
| Edges           |   |
| overlaps        | 1 |
| is_a (inferred) | 2 |
| is_a (input)    | 8 |

Supplement: S6 File — (A) Alignment visualization for the alignment of passeriform clade concepts ("Phylo2015") sec. 2015.PEA with the corresponding classification concepts ("Class2015") sec. Gill & Donsker (2015); including the (paraphyletic) Class2015.Eurylaimidae. (B) Set of Maximally Informative Relations (MIR) inferred for the alignment of passeriform clade concepts ("Phylo2015") sec. 2015.PEA with the corresponding classification concepts ("Class2015") sec. Gill & Donsker (2015); including the (paraphyletic) Class2015.Eurylaimidae. Total = 63 MIR. (ZIP) [file pcbi.1006493.s006.zip › S6A-Eurylaimidae-Paraphyletic-0-mnpw.pdf]

| Nodes            |    |  |
|------------------|----|--|
| Phylo2015        | 14 |  |
| Class2015        | 10 |  |
| Edges            |    |  |
| is_a (Phylo2015) | 13 |  |
| is_a (Class2015) | 9  |  |
| articulations    | 8  |  |

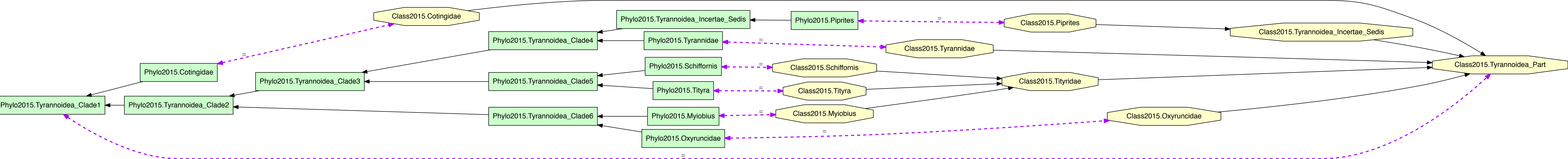

Supplement: S7 File — Supporting files for the alignment of tyrannoid clade concepts ("Phylo2015") sec. 2015.PEA with the corresponding classification concepts ("Class2015") sec. Gill & Donsker (2015); including the (paraphyletic) Class2015.Tityridae. (A) Reasoner input constraints. Includes information on run commands; and 0 instances of "no coverage". (B) Input visualization. (C) Alignment visualization. (D) Set of Maximally Informative Relations (MIR). Total = 140 MIR. (ZIP) [file pcbi.1006493.s007.zip › S7B-Tityridae-Paraphyletic.pdf]

| Nodes            |    |
|------------------|----|
| Phylo2015        | 17 |
| Class2015        | 13 |
| Edges            |    |
| is_a (Phylo2015) | 16 |
| is_a (Class2015) | 12 |
| articulations    | 9  |

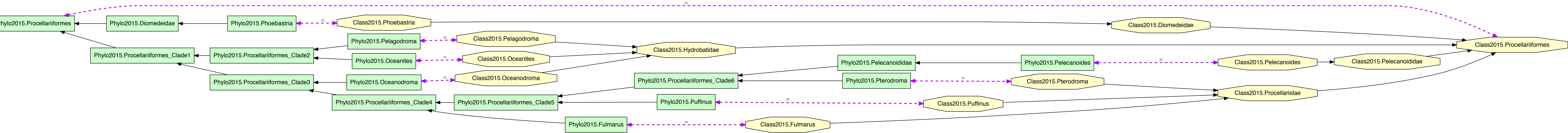

Supplement: S8 File — (A) Reasoner input constraints for the alignment of procellariiform clade concepts ("Phylo2015") sec. 2015.PEA with the corresponding classification concepts ("Class2015") sec. Gill & Donsker (2015); including the (paraphyletic) Class2015.Hydrobatidae and Class2015.Procellariidae. Includes information on run commands; and 0 instances of "no coverage". (B) Input visualization for the alignment of procellariiform clade concepts ("Phylo2015") sec. 2015.PEA with the corresponding classification concepts ("Class2015") sec. Gill & Donsker (2015); including the (paraphyletic) Class2015.Hydrobatidae and Class2015.Procellariidae. (C) Alignment visualization for the alignment of procellariiform clade concepts ("Phylo2015") sec. 2015.PEA with the corresponding classification concepts ("Class2015") sec. Gill & Donsker (2015); including the (paraphyletic) Class2015.Hydrobatidae and Class2015.Procellariidae. (D) Set of Maximally Informative Relations (MIR) inferred for the alignment of procellariiform clade concepts ("Phylo2015") sec. 2015.PEA with the corresponding classification concepts ("Class2015") sec. Gill & Donsker (2015); including the (paraphyletic) Class2015.Hydrobatidae and Class2015.Procellariidae. Total = 221 MIR. (ZIP) [file pcbi.1006493.s008.zip › S8B-Hydrobatidae-Procellariidae-Paraphyletic.pdf]

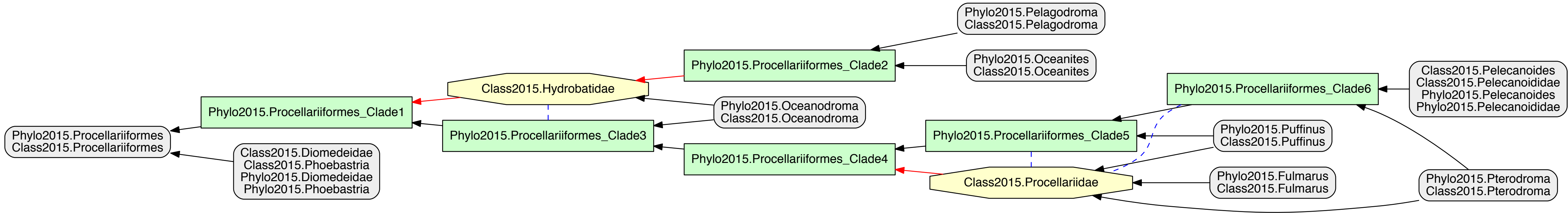

| Nodes           |    |
|-----------------|----|
| congruent       | 9  |
| Phylo2015       | 6  |
| Class2015       | 2  |
| Edges           |    |
| is_a (inferred) | 3  |
| is_a (input)    | 16 |
| overlaps        | 3  |

Supplement: S8 File — (A) Reasoner input constraints for the alignment of procellariiform clade concepts ("Phylo2015") sec. 2015.PEA with the corresponding classification concepts ("Class2015") sec. Gill & Donsker (2015); including the (paraphyletic) Class2015.Hydrobatidae and Class2015.Procellariidae. Includes information on run commands; and 0 instances of "no coverage". (B) Input visualization for the alignment of procellariiform clade concepts ("Phylo2015") sec. 2015.PEA with the corresponding classification concepts ("Class2015") sec. Gill & Donsker (2015); including the (paraphyletic) Class2015.Hydrobatidae and Class2015.Procellariidae. (C) Alignment visualization for the alignment of procellariiform clade concepts ("Phylo2015") sec. 2015.PEA with the corresponding classification concepts ("Class2015") sec. Gill & Donsker (2015); including the (paraphyletic) Class2015.Hydrobatidae and Class2015.Procellariidae. (D) Set of Maximally Informative Relations (MIR) inferred for the alignment of procellariiform clade concepts ("Phylo2015") sec. 2015.PEA with the corresponding classification concepts ("Class2015") sec. Gill & Donsker (2015); including the (paraphyletic) Class2015.Hydrobatidae and Class2015.Procellariidae. Total = 221 MIR. (ZIP) [file pcbi.1006493.s008.zip › S8C-Hydrobatidae-Procellariidae-Paraphyletic-0-mnpw.pdf]

|                  |    |
|------------------|----|
| <b>Nodes</b>     |    |
| Phylo2015        | 28 |
| Class2015        | 24 |
| <b>Edges</b>     |    |
| is_a (Phylo2015) | 27 |
| is_a (Class2015) | 23 |
| articulations    | 14 |

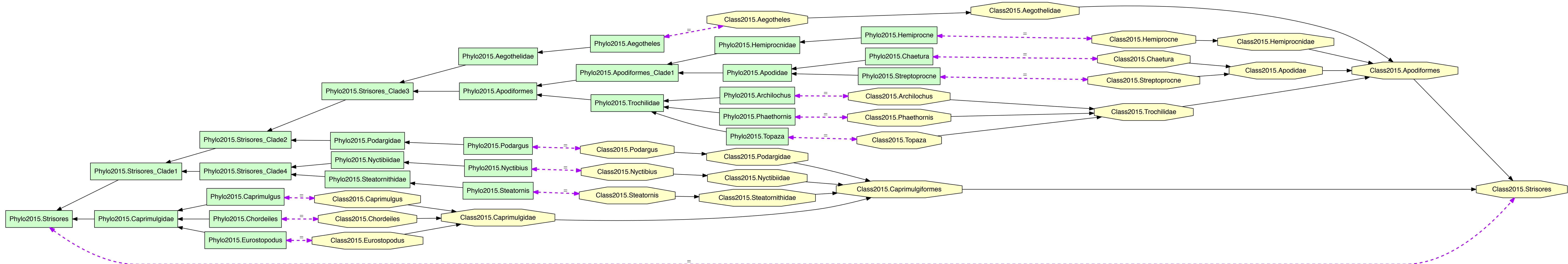

Supplement: S9 File — (A) Reasoner input constraints for the alignment of caprimulgiform clade concepts ("Phylo2015") sec. 2015.PEA with the corresponding classification concepts ("Class2015") sec. Gill & Donsker (2015); including the (paraphyletic) Class2015.Caprimulgiformes. Includes information on run commands; and 0 instances of "no coverage". (B) Input visualization for the alignment of caprimulgiform clade concepts ("Phylo2015") sec. 2015.PEA with the corresponding classification concepts ("Class2015") sec. Gill & Donsker (2015); including the (paraphyletic) Class2015.Caprimulgiformes. (C) Alignment visualization for the alignment of caprimulgiform clade concepts ("Phylo2015") sec. 2015.PEA with the corresponding classification concepts ("Class2015") sec. Gill & Donsker (2015); including the (paraphyletic) Class2015.Caprimulgiformes. (D) Set of Maximally Informative Relations (MIR) inferred for the alignment of caprimulgiform clade concepts ("Phylo2015") sec. 2015.PEA with the corresponding classification concepts ("Class2015") sec. Gill & Donsker (2015); including the (paraphyletic) Class2015.Caprimulgiformes. Total = 672 MIR. (ZIP) [file pcbi.1006493.s009.zip › S9B-Caprimulgiformes-Paraphyletic.pdf]

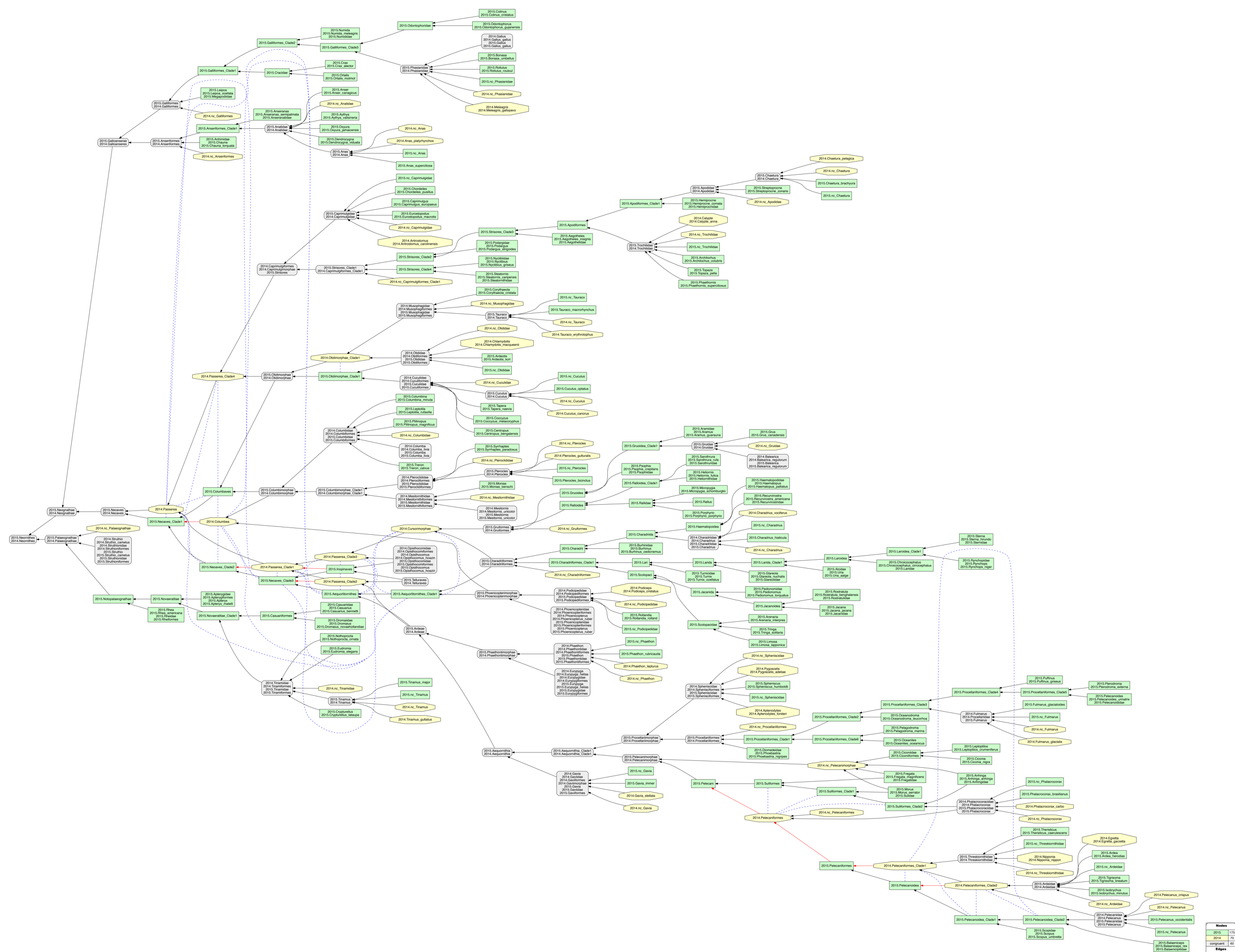

Supplement: S10 File — (A) Reasoner input constraints for the 2015./2014.Neornithes alignment (excepting 2015./2014.Telluraves), with coverage locally relaxed. Includes information on run commands; and 58 instances of "no coverage". (B) Input visualization for the 2015./2014.Neornithes alignment (excepting 2015./2014.Telluraves), with coverage locally relaxed. (C) Alignment visualization for the 2015./2014.Neornithes alignment (excepting 2015./2014.Telluraves), with coverage locally relaxed. (D) Set of Maximally Informative Relations (MIR) inferred for the 2015./2014.Neornithes alignment (excepting 2015./2014.Telluraves), with coverage locally relaxed. Total = 68,208 MIR. (ZIP) [file pcbi.1006493.s010.zip › S10C-Neornithes-Excepting-Telluraves-0-mnpw.pdf]

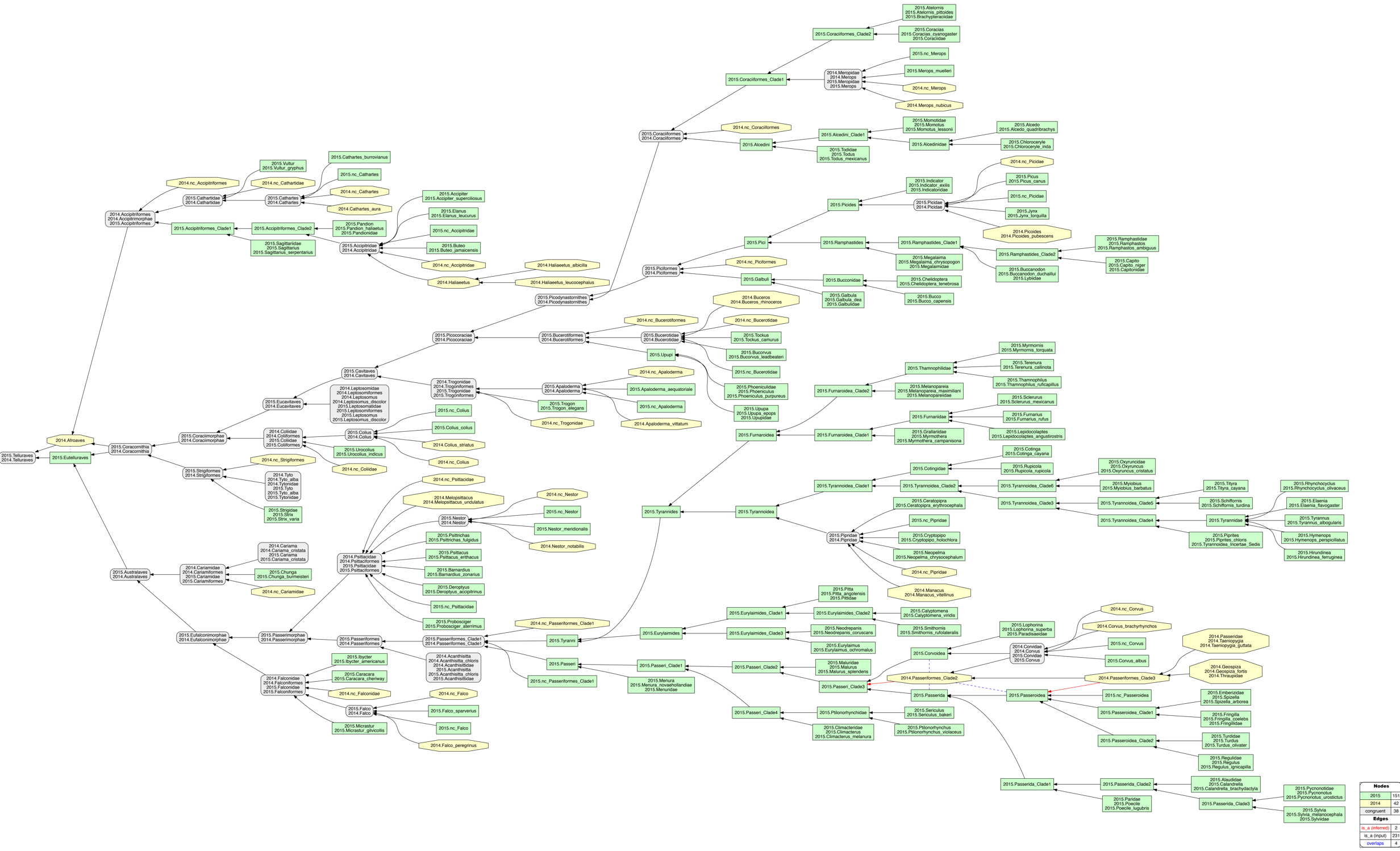

| Nodes           |     |
|-----------------|-----|
| 2015            | 151 |
| 2014            | 42  |
| congruent       | 38  |
| Edges           |     |
| is_a (inferred) | 2   |
| is_a (input)    | 231 |
| overlaps        | 4   |

Supplement: S11 File — (A) Reasoner input constraints for the 2015./2014.Telluraves alignment, with coverage locally relaxed. Includes information on run commands; and 37 instances of "no coverage". (B) Input visualization for the 2015./2014.Telluraves alignment, with coverage locally relaxed. (C) Alignment visualization for the 2015./2014.Telluraves alignment, with coverage locally relaxed. (D) Set of Maximally Informative Relations (MIR) inferred for the 2015./2014.Telluraves alignment, with coverage locally relaxed. Total = 32,864 MIR. (ZIP) [file pcbi.1006493.s011.zip › S11C-Telluraves-0-mnpw.pdf]

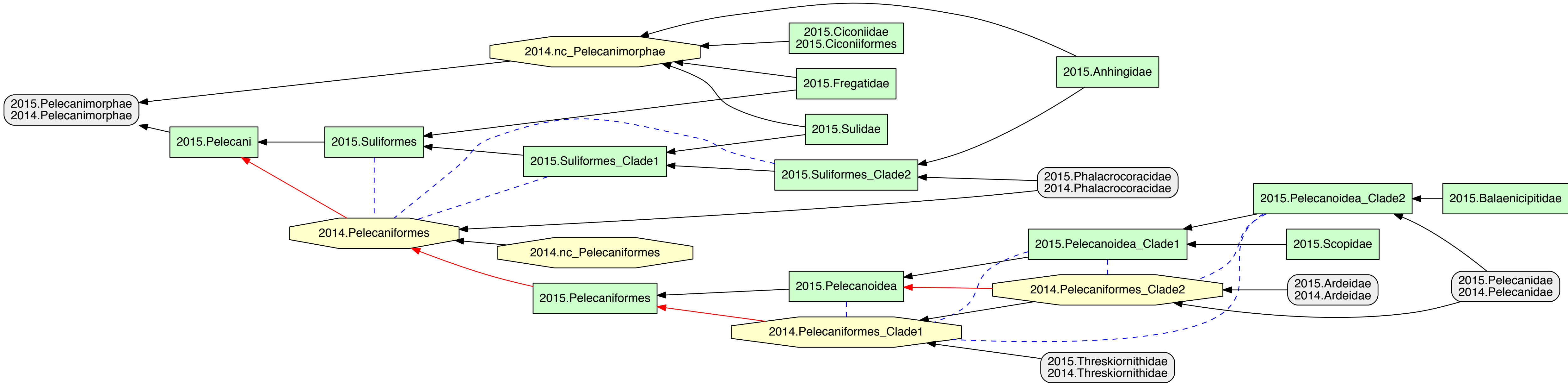

| Nodes           |    |
|-----------------|----|
| 2015            | 14 |
| 2014            | 5  |
| congruent       | 5  |
| Edges           |    |
| is_a (inferred) | 4  |
| is_a (input)    | 25 |
| overlaps        | 8  |

Supplement: S12 File — (A) Reasoner input constraints for the 2015./2014.Pelecanimorphae alignment, with coverage locally relaxed. Includes information on run commands; and 2 instances of "no coverage". (B) Input visualization for the 2015./2014.Pelecanimorphae alignment, with coverage locally relaxed. (C) Alignment visualization for the 2015./2014.Pelecanimorphae alignment, with coverage locally relaxed. (D) Set of Maximally Informative Relations (MIR) inferred for the 2015./2014.Pelecanimorphae alignment, with coverage locally relaxed. Total = 200 MIR. (ZIP) [file pcbi.1006493.s012.zip › S12C-Pelecanimorphae-Overlap-0-mnpw.pdf]

| Nodes         |    |
|---------------|----|
| 2015          | 20 |
| 2014          | 10 |
| Edges         |    |
| is_a (2015)   | 19 |
| is_a (2014)   | 9  |
| articulations | 16 |

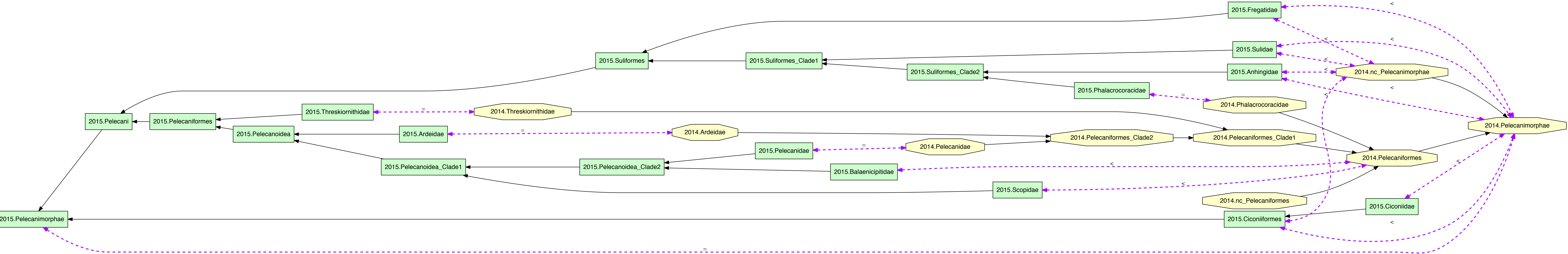

Supplement: S12 File — (A) Reasoner input constraints for the 2015./2014.Pelecanimorphae alignment, with coverage locally relaxed. Includes information on run commands; and 2 instances of "no coverage". (B) Input visualization for the 2015./2014.Pelecanimorphae alignment, with coverage locally relaxed. (C) Alignment visualization for the 2015./2014.Pelecanimorphae alignment, with coverage locally relaxed. (D) Set of Maximally Informative Relations (MIR) inferred for the 2015./2014.Pelecanimorphae alignment, with coverage locally relaxed. Total = 200 MIR. (ZIP) [file pcbi.1006493.s012.zip › S12B-Pelecanimorphae-Overlap.pdf]

| Nodes         |    |
|---------------|----|
| 2015          | 28 |
| 2014          | 5  |
| Edges         |    |
| is_a (2015)   | 27 |
| is_a (2014)   | 4  |
| articulations | 29 |

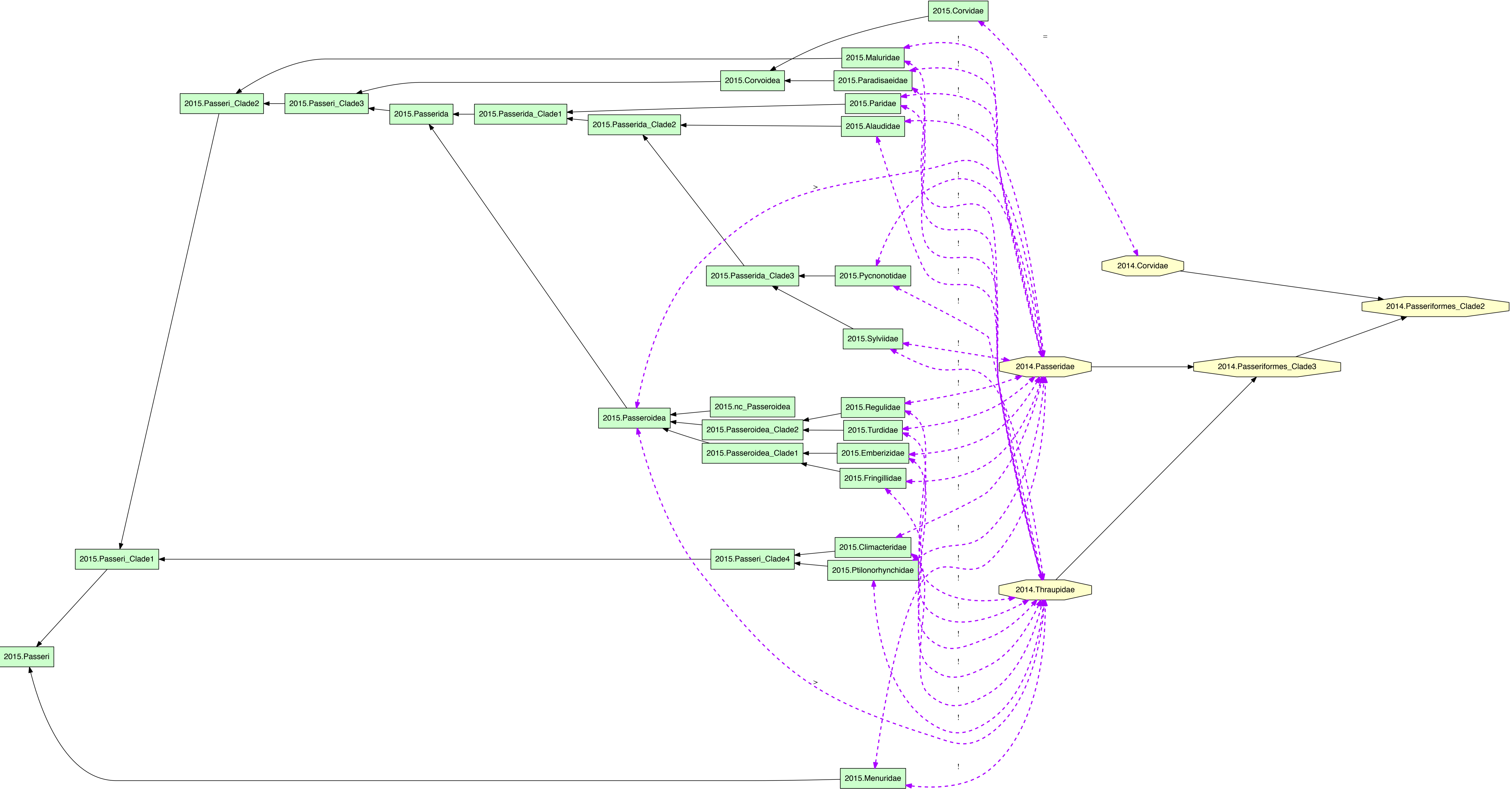

Supplement: S13 File — (A) Reasoner input constraints for the 2015.Passeri/2014.Passeriformes_Clade2 alignment, with coverage locally relaxed. Includes information on run commands; and 1 instance of "no coverage". (B) Input visualization for the 2015.Passeri/2014.Passeriformes_Clade2 alignment, with coverage locally relaxed. (C) Alignment visualization for the 2015.Passeri/2014.Passeriformes_Clade2 alignment, with coverage locally relaxed. (D) Set of Maximally Informative Relations (MIR) inferred for the 2015.Passeri/2014.Passeriformes_Clade2 alignment, with coverage locally relaxed. Total = 140 MIR. (ZIP) [file pcbi.1006493.s013.zip › S13B-Passeri-Overlap.pdf]

| Nodes         |   |
|---------------|---|
| 2015          | 9 |
| 2014          | 9 |
| Edges         |   |
| is_a (2015)   | 8 |
| is_a (2014)   | 8 |
| default       | 1 |
| articulations | 9 |

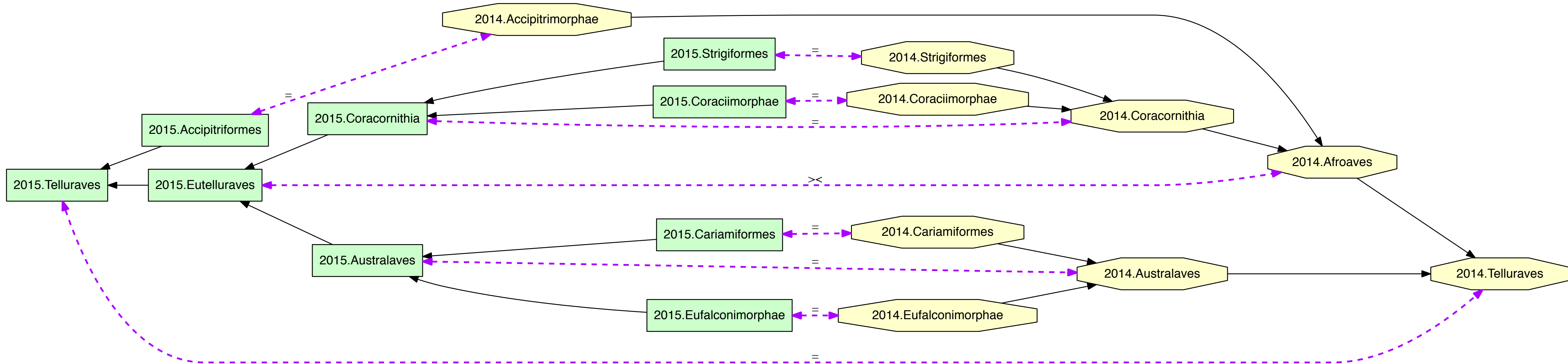

Supplement: S14 File — (A) Reasoner input constraints for the 2015./2014.Telluraves alignment (higher-level subset), under whole-concept resolution. Includes information on run commands; and 0 instances of "no coverage". (B) Input visualization for the 2015./2014.Telluraves alignment (higher-level subset), under whole-concept resolution. (C) Alignment visualization for the 2015./2014.Telluraves alignment (higher-level subset), under whole-concept resolution. (D) Set of Maximally Informative Relations (MIR) inferred for the 2015./2014.Telluraves alignment (higher-level subset), under whole-concept resolution. Total = 81 MIR. (ZIP) [file pcbi.1006493.s014.zip › S14B-Telluraves-Overlap-Whole-Concept-Resolution.pdf]

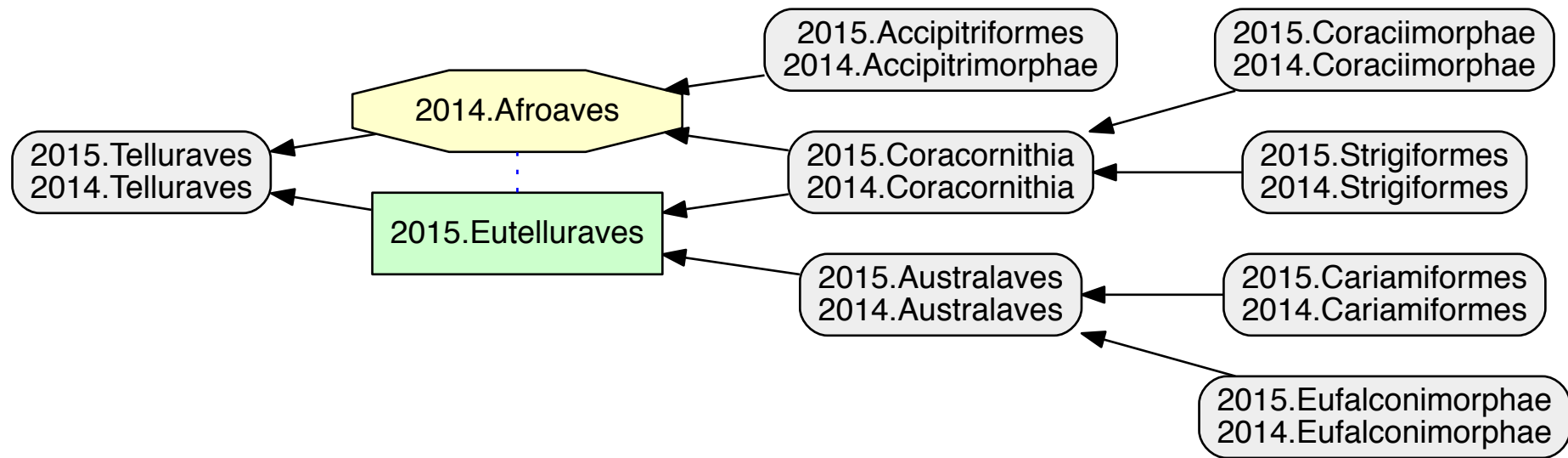

| Nodes        |    |
|--------------|----|
| 2015         | 1  |
| 2014         | 1  |
| congruent    | 8  |
| Edges        |    |
| is_a (input) | 10 |
| overlaps     | 1  |

Supplement: S14 File — (A) Reasoner input constraints for the 2015./2014.Telluraves alignment (higher-level subset), under whole-concept resolution. Includes information on run commands; and 0 instances of "no coverage". (B) Input visualization for the 2015./2014.Telluraves alignment (higher-level subset), under whole-concept resolution. (C) Alignment visualization for the 2015./2014.Telluraves alignment (higher-level subset), under whole-concept resolution. (D) Set of Maximally Informative Relations (MIR) inferred for the 2015./2014.Telluraves alignment (higher-level subset), under whole-concept resolution. Total = 81 MIR. (ZIP) [file pcbi.1006493.s014.zip › S14C-Telluraves-Overlap-Whole-Concept-Resolution-0-mnpw.pdf]

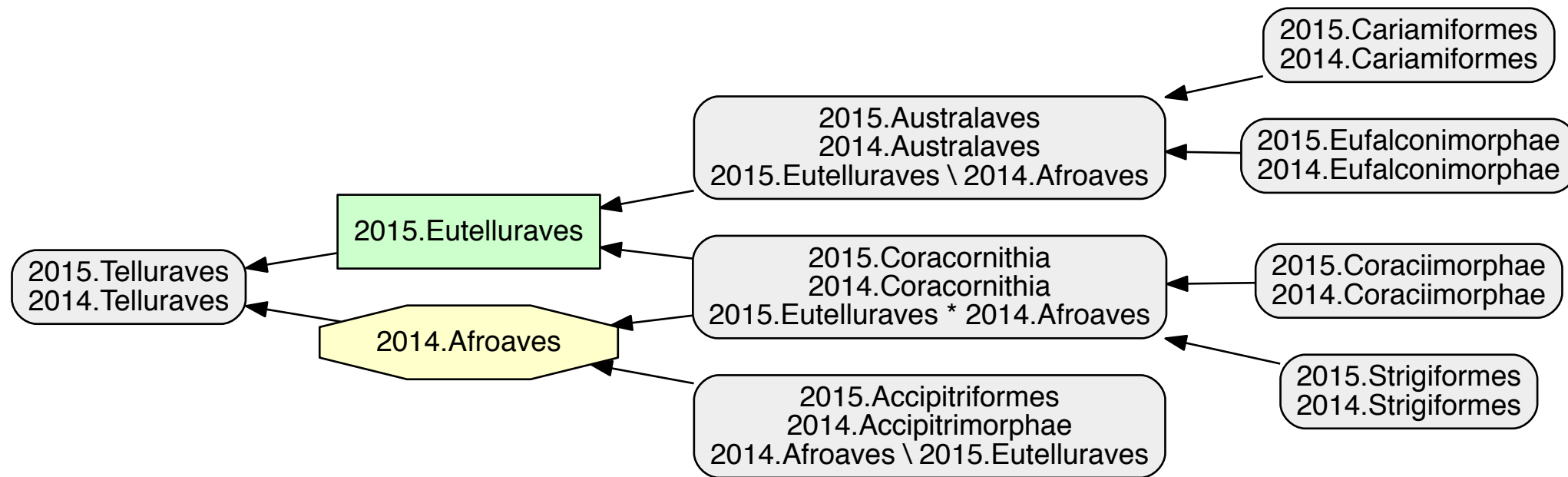

| Nodes        |    |
|--------------|----|
| 2015         | 1  |
| 2014         | 1  |
| congruent    | 8  |
| Edges        |    |
| is_a (input) | 10 |

Supplement: S15 File — (A) Reasoner input constraints for the 2015./2014.Telluraves alignment (higher-level subset), under split-concept resolution. Includes information on run commands; and 0 instances of "no coverage". (B) Input visualization for the 2015./2014.Telluraves alignment (higher-level subset), under split-concept resolution. (C) Alignment visualization for the 2015./2014.Telluraves alignment (higher-level subset), under split-concept resolution. (D) Set of Maximally Informative Relations (MIR) inferred for the 2015./2014.Telluraves alignment (higher-level subset), under split-concept resolution. Total = 81 MIR. (ZIP) [file pcbi.1006493.s015.zip › S15C-Telluraves-Overlap-Split-Concept-Resolution-0-mncb.pdf]

| Nodes         |    |
|---------------|----|
| 2015          | 21 |
| 2014          | 21 |
| Edges         |    |
| is_a (2015)   | 20 |
| is_a (2014)   | 20 |
| articulations | 13 |

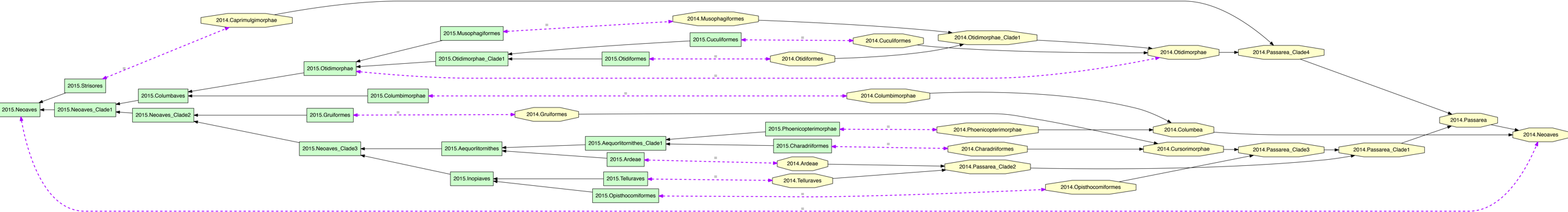

Supplement: S17 File — (A) Reasoner input constraints for the 2015./2014.Neoaves alignment, under whole-concept resolution, limited to the main conflict region. Includes information on run commands; and 0 instances of "no coverage". (B) Input visualization for the 2015./2014.Neoaves alignment, under whole-concept resolution, limited to the main conflict region. (C) Alignment visualization for the 2015./2014.Neoaves alignment, under whole-concept resolution, limited to the main conflict region. (D) Set of Maximally Informative Relations (MIR) inferred for the 2015./2014.Neoaves alignment, under whole-concept resolution, limited to the main conflict region. Total = 441 MIR. (ZIP) [file pcbi.1006493.s017.zip › S17B-Neoaves-Conflict-Region-Overlap-Whole-Concept-Resolution.pdf]

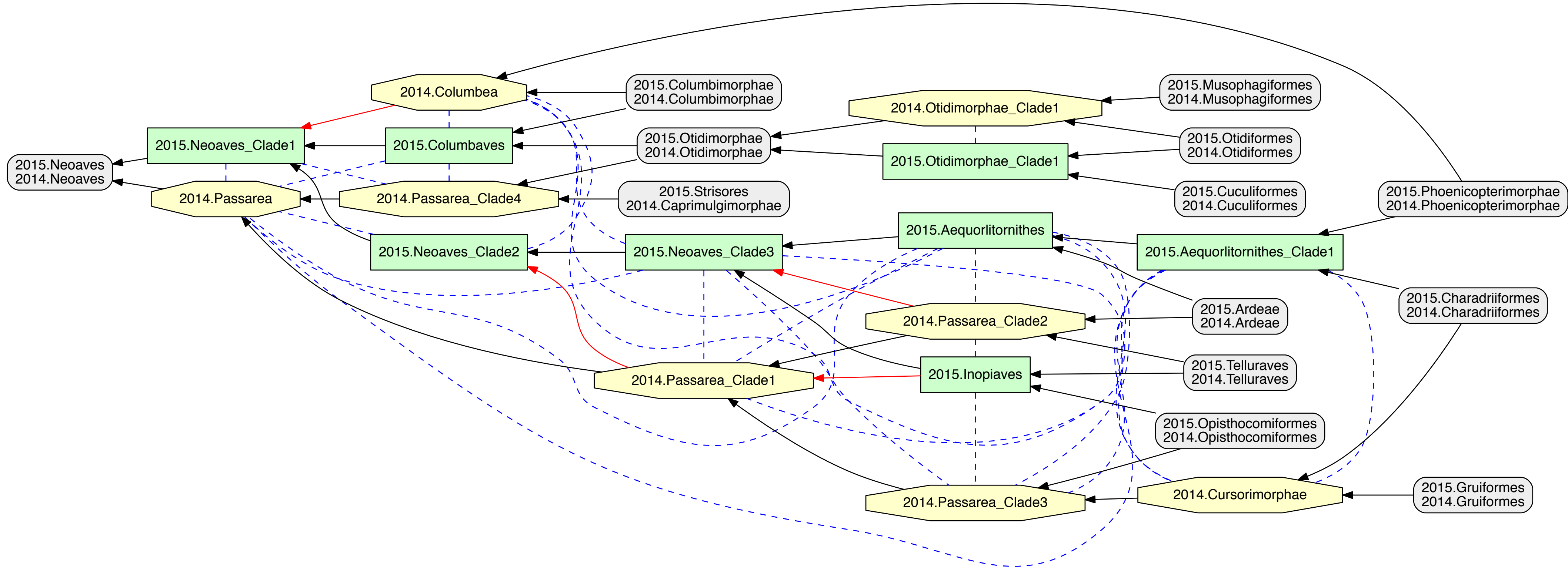

| Nodes           |    |  |
|-----------------|----|--|
| 2015            | 8  |  |
| 2014            | 8  |  |
| congruent       | 13 |  |
| Edges           |    |  |
| is_a (inferred) | 4  |  |
| overlaps        | 26 |  |
| is_a (input)    | 35 |  |

Supplement: S17 File — (A) Reasoner input constraints for the 2015./2014.Neoaves alignment, under whole-concept resolution, limited to the main conflict region. Includes information on run commands; and 0 instances of "no coverage". (B) Input visualization for the 2015./2014.Neoaves alignment, under whole-concept resolution, limited to the main conflict region. (C) Alignment visualization for the 2015./2014.Neoaves alignment, under whole-concept resolution, limited to the main conflict region. (D) Set of Maximally Informative Relations (MIR) inferred for the 2015./2014.Neoaves alignment, under whole-concept resolution, limited to the main conflict region. Total = 441 MIR. (ZIP) [file pcbi.1006493.s017.zip › S17C-Neoaves-Conflict-Region-Overlap-Whole-Concept-Resolution-0-mnpw.pdf]
