# Supplementary material for: Verbalizing phylogenomic conflict: Representation of node congruence across competing reconstructions of the neoavian explosion
Source: PLoS Comput Biol. 2019 Feb 15;15(2):e1006493. doi: 10.1371/journal.pcbi.1006493 (PMC6395011; doi:10.1371/journal.pcbi.1006493)
Supplement: S9 File — (A) Reasoner input constraints for the alignment of caprimulgiform clade concepts ("Phylo2015") sec. 2015.PEA with the corresponding classification concepts ("Class2015") sec. Gill & Donsker (2015); including the (paraphyletic) Class2015.Caprimulgiformes. Includes information on run commands; and 0 instances of "no coverage". (B) Input visualization for the alignment of caprimulgiform clade concepts ("Phylo2015") sec. 2015.PEA with the corresponding classification concepts ("Class2015") sec. Gill & Donsker (2015); including the (paraphyletic) Class2015.Caprimulgiformes. (C) Alignment visualization for the alignment of caprimulgiform clade concepts ("Phylo2015") sec. 2015.PEA with the corresponding classification concepts ("Class2015") sec. Gill & Donsker (2015); including the (paraphyletic) Class2015.Caprimulgiformes. (D) Set of Maximally Informative Relations (MIR) inferred for the alignment of caprimulgiform clade concepts ("Phylo2015") sec. 2015.PEA with the corresponding classification concepts ("Class2015") sec. Gill & Donsker (2015); including the (paraphyletic) Class2015.Caprimulgiformes. Total = 672 MIR. (ZIP) [file S9C-Caprimulgiformes-Paraphyletic-0-mnpw.pdf › S9C-Caprimulgiformes-Paraphyletic-0-mnpw.pdf]

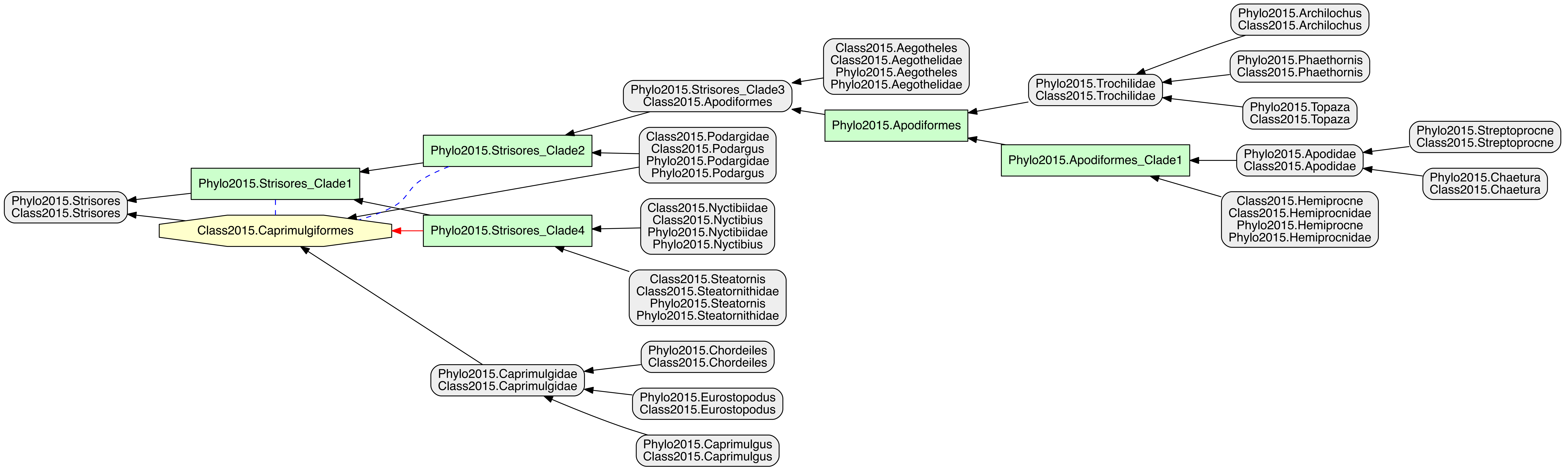

Nodes	
congruent	18
Phylo2015	5
Class2015	1
**Edges**	
overlaps	2
is_a (input)	24
is_a (inferred)	1
