# Supplementary material for: Verbalizing phylogenomic conflict: Representation of node congruence across competing reconstructions of the neoavian explosion
Source: PLoS Comput Biol. 2019 Feb 15;15(2):e1006493. doi: 10.1371/journal.pcbi.1006493 (PMC6395011; doi:10.1371/journal.pcbi.1006493)

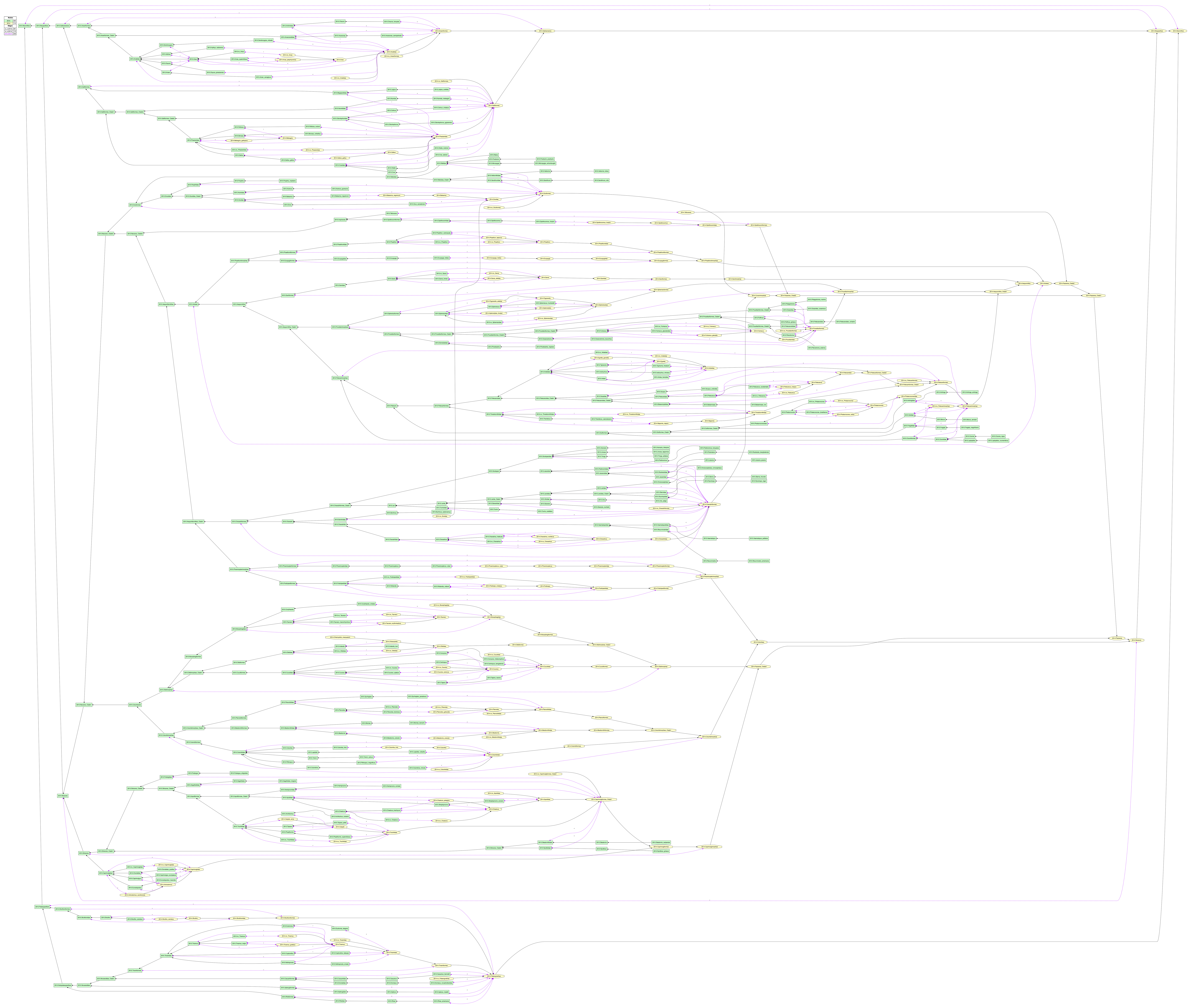

Supplement: S10 File — (A) Reasoner input constraints for the 2015./2014.Neornithes alignment (excepting 2015./2014.Telluraves), with coverage locally relaxed. Includes information on run commands; and 58 instances of "no coverage". (B) Input visualization for the 2015./2014.Neornithes alignment (excepting 2015./2014.Telluraves), with coverage locally relaxed. (C) Alignment visualization for the 2015./2014.Neornithes alignment (excepting 2015./2014.Telluraves), with coverage locally relaxed. (D) Set of Maximally Informative Relations (MIR) inferred for the 2015./2014.Neornithes alignment (excepting 2015./2014.Telluraves), with coverage locally relaxed. Total = 68,208 MIR. (ZIP) [file S10B-Neornithes-Excepting-Telluraves.pdf › S10B-Neornithes-Excepting-Telluraves.pdf]
