# Supplementary material for: Verbalizing phylogenomic conflict: Representation of node congruence across competing reconstructions of the neoavian explosion
Source: PLoS Comput Biol. 2019 Feb 15;15(2):e1006493. doi: 10.1371/journal.pcbi.1006493 (PMC6395011; doi:10.1371/journal.pcbi.1006493)

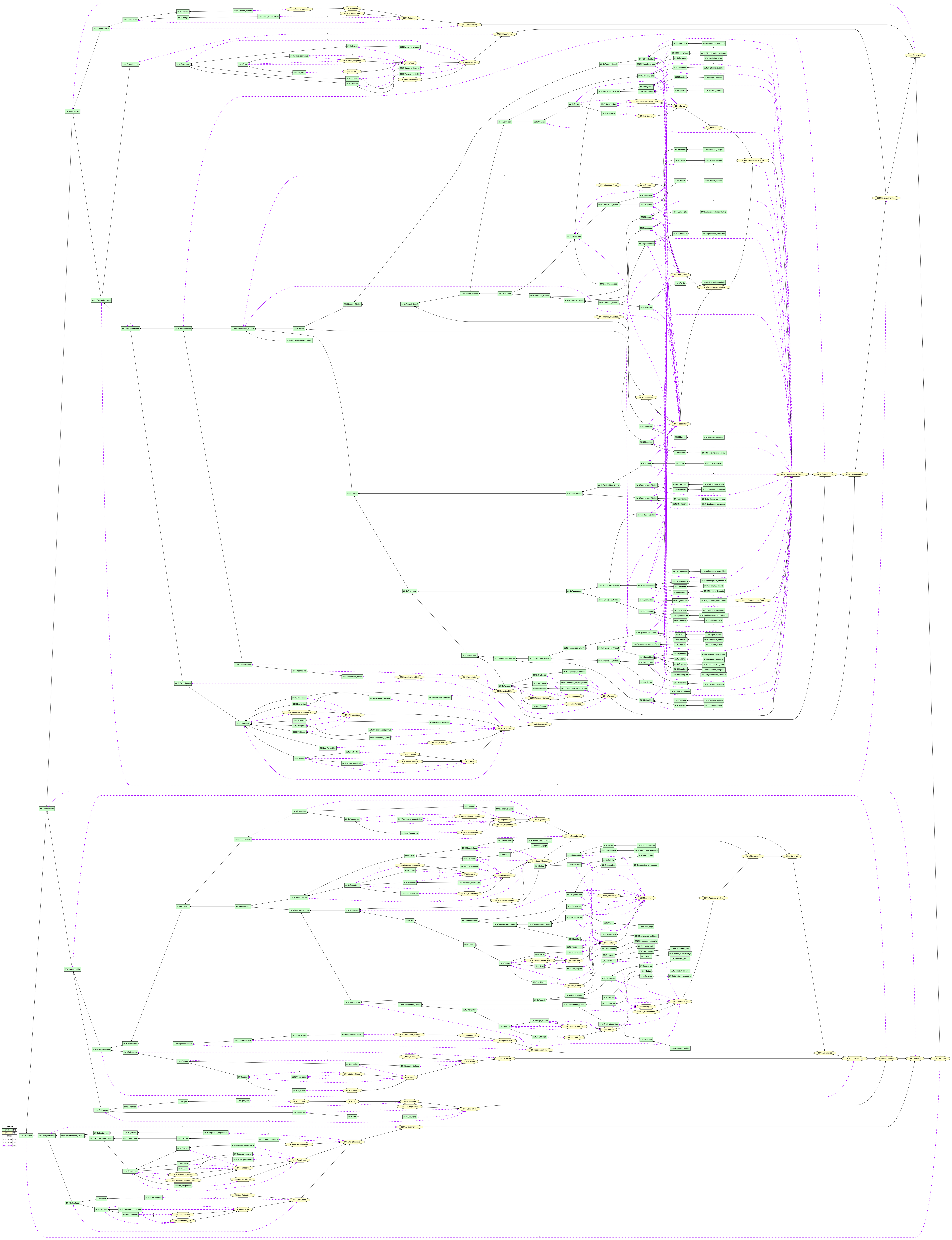

Supplement: S11 File — (A) Reasoner input constraints for the 2015./2014.Telluraves alignment, with coverage locally relaxed. Includes information on run commands; and 37 instances of "no coverage". (B) Input visualization for the 2015./2014.Telluraves alignment, with coverage locally relaxed. (C) Alignment visualization for the 2015./2014.Telluraves alignment, with coverage locally relaxed. (D) Set of Maximally Informative Relations (MIR) inferred for the 2015./2014.Telluraves alignment, with coverage locally relaxed. Total = 32,864 MIR. (ZIP) [file S11B-Telluraves.pdf › S11B-Telluraves.pdf]
