# Supplementary material for: Verbalizing phylogenomic conflict: Representation of node congruence across competing reconstructions of the neoavian explosion
Source: PLoS Comput Biol. 2019 Feb 15;15(2):e1006493. doi: 10.1371/journal.pcbi.1006493 (PMC6395011; doi:10.1371/journal.pcbi.1006493)
Supplement: S13 File — (A) Reasoner input constraints for the 2015.Passeri/2014.Passeriformes_Clade2 alignment, with coverage locally relaxed. Includes information on run commands; and 1 instance of "no coverage". (B) Input visualization for the 2015.Passeri/2014.Passeriformes_Clade2 alignment, with coverage locally relaxed. (C) Alignment visualization for the 2015.Passeri/2014.Passeriformes_Clade2 alignment, with coverage locally relaxed. (D) Set of Maximally Informative Relations (MIR) inferred for the 2015.Passeri/2014.Passeriformes_Clade2 alignment, with coverage locally relaxed. Total = 140 MIR. (ZIP) [file S13C-Passeri-Overlap-0-mnpw.pdf › S13C-Passeri-Overlap-0-mnpw.pdf]

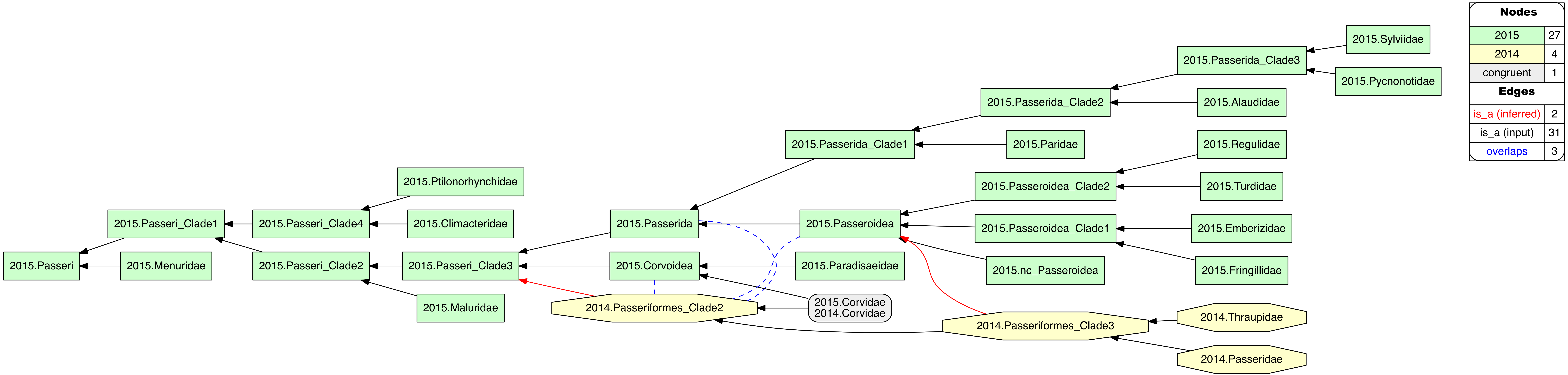

Nodes
2015 27
2014 4
congruent 1
Edges
is_a (inferred) 2
is_a (input) 31
overlaps 3
2015.Sylviidae
2015.Passerida_Clade3
2015.Pycnonotidae
2015.Passerida_Clade2
2015.Alaudidae
2015.Passerida_Clade1
2015.Paridae
2015.Regulidae
2015.Passeroidea_Clade2
2015.Turdidae
2015.Ptilonorhynchidae
2015.Passeri_Clade1
2015.Passeri_Clade4
2015.Climacteridae
2015.Passerida
2015.Passeroidea
2015.Passeroidea_Clade1
2015.Emberizidae
2015.Passeri
2015.Menuridae
2015.Passeri_Clade2
2015.Passeri_Clade3
2015.Corvoidea
2015.Paradisaeidae
2015.nc_Passeroidea
2015.Fringillidae
2015.Maluridae
2014.Passeriformes_Clade2
2015.Corvidae
2014.Corvidae
2014.Passeriformes_Clade3
2014.Thraupidae
2014.Passeridae
