# Supplementary material for: Verbalizing phylogenomic conflict: Representation of node congruence across competing reconstructions of the neoavian explosion
Source: PLoS Comput Biol. 2019 Feb 15;15(2):e1006493. doi: 10.1371/journal.pcbi.1006493 (PMC6395011; doi:10.1371/journal.pcbi.1006493)
Supplement: S16 File — (A) Reasoner input constraints for the 2015./2014.Neornithes alignment, under whole-concept resolution, ranging from the root to the ordinal level (with exceptions where needed). Includes information on run commands; and 4 instances of "no coverage". (B) Input visualization for the 2015./2014.Neornithes alignment, under whole-concept resolution, ranging from the root to the ordinal level (with exceptions where needed). (C) Alignment visualization for the 2015./2014.Neornithes alignment, under whole-concept resolution, ranging from the root to the ordinal level (with exceptions where needed). (D) Set of Maximally Informative Relations (MIR) inferred for the 2015./2014.Neornithes alignment, under whole-concept resolution, ranging from the root to the ordinal level (with exceptions where needed). Total = 8,051 MIR. (ZIP) [file S16C-Neornithes-Higher-Level-Overlap-0-mnpw.pdf › S16C-Neornithes-Higher-Level-Overlap-0-mnpw.pdf]

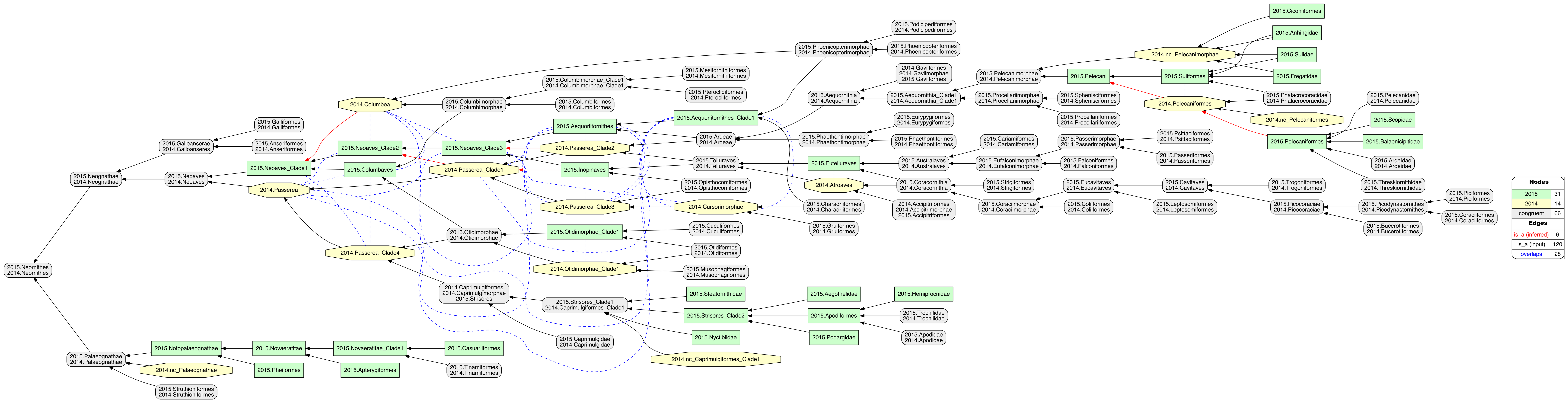

2015.Neornithes 2014.Neornithes
2015.Neognathae 2014.Neognathae
2015.Palaeognathae 2014.Palaeognathae
2015.Galloanserae 2014.Galloanserae
2015.Galliformes 2014.Galliformes
2015.Anseriformes 2014.Anseriformes
2015.Neoaves 2014.Neoaves
2014.Passerea
2015.Neoaves_Clade1
2015.Neoaves_Clade2
2015.Neoaves_Clade3
2015.Columbaves
2014.Columbea
2015.Columbimorphae 2014.Columbimorphae
2015.Columbimorphae_Clade1 2014.Columbimorphae_Clade1
2015.Mesitornithiformes 2014.Mesitornithiformes
2015.Pterocliformes 2014.Pterocliformes
2015.Columbiformes 2014.Columbiformes
2015.Phoenicopterimorphae 2014.Phoenicopterimorphae
2015.Podicipediformes 2014.Podicipediformes
2015.Phoenicopteriformes 2014.Phoenicopteriformes
2014.Passerea_Clade1
2014.Passerea_Clade2
2014.Passerea_Clade3
2014.Passerea_Clade4
2015.Aequorlitornithes
2015.Aequorlitornithes_Clade1
2015.Inopinaves
2015.Ardeae 2014.Ardeae
2015.Telluraves 2014.Telluraves
2015.Opisthocomiformes 2014.Opisthocomiformes
2014.Cursorimorphae
2015.Otidimorphae 2014.Otidimorphae
2015.Otidimorphae_Clade1
2014.Otidimorphae_Clade1
2015.Cuculiformes 2014.Cuculiformes
2015.Otidiformes 2014.Otidiformes
2015.Musophagiformes 2014.Musophagiformes
2014.Caprimulgiformes 2014.Caprimulgimorphae 2015.Strisores
2015.Strisores_Clade1 2014.Caprimulgiformes_Clade1
2015.Caprimulgidae 2014.Caprimulgidae
2014.nc_Caprimulgiformes_Clade1
2015.Steatornithidae
2015.Strisores_Clade2
2015.Nyctibiidae
2015.Aegothelidae
2015.Apodiformes
2015.Podargidae
2015.Hemiprocnidae
2015.Trochilidae 2014.Trochilidae
2015.Apodidae 2014.Apodidae
2015.Notopalaeognathae
2015.Novaeratitae
2015.Novaeratitae_Clade1
2015.Casuariiformes
2015.Rheiformes
2015.Apterygiformes
2015.Tinamiformes 2014.Tinamiformes
2014.nc_Palaeognathae
2015.Struthioniformes 2014.Struthioniformes
2015.Aequornithia 2014.Aequornithia
2014.Gaviiformes 2014.Gaviimorphae 2015.Gaviiformes
2015.Aequornithia_Clade1 2014.Aequornithia_Clade1
2015.Procellariimorphae 2014.Procellariimorphae
2015.Sphenisciformes 2014.Sphenisciformes
2015.Procellariiformes 2014.Procellariiformes
2015.Pelecanimorphae 2014.Pelecanimorphae
2015.Pelecani
2015.Suliformes
2014.nc_Pelecanimorphae
2015.Ciconiiformes
2015.Anhingidae
2015.Sulidae
2015.Fregatidae
2015.Phalacrocoracidae 2014.Phalacrocoracidae
2014.Pelecaniformes
2014.nc_Pelecaniformes
2015.Pelecaniformes
2015.Pelecanidae 2014.Pelecanidae
2015.Scopidae
2015.Balaenicipitidae
2015.Ardeidae 2014.Ardeidae
2015.Threskiornithidae 2014.Threskiornithidae
2015.Phaethontimorphae 2014.Phaethontimorphae
2015.Eurypygiformes 2014.Eurypygiformes
2015.Phaethontiformes 2014.Phaethontiformes
2015.Charadriiformes 2014.Charadriiformes
2015.Gruiformes 2014.Gruiformes
2015.Eutelluraves
2014.Afroaves
2015.Australaves 2014.Australaves
2015.Cariamiformes 2014.Cariamiformes
2015.Eufalconimorphae 2014.Eufalconimorphae
2015.Passerimorphae 2014.Passerimorphae
2015.Psittaciformes 2014.Psittaciformes
2015.Passeriformes 2014.Passeriformes
2015.Falconiformes 2014.Falconiformes
2015.Coracornithia 2014.Coracornithia
2015.Accipitriformes 2014.Accipitrimorphae 2015.Accipitriformes
2015.Strigiformes 2014.Strigiformes
2015.Coraciimorphae 2014.Coraciimorphae
2015.Coliiformes 2014.Coliiformes
2015.Eucavitaves 2014.Eucavitaves
2015.Cavitaves 2014.Cavitaves
2015.Leptosomiformes 2014.Leptosomiformes
2015.Trogoniformes 2014.Trogoniformes
2015.Picocoraciae 2014.Picocoraciae
2015.Picodynastornithes 2014.Picodynastornithes
2015.Piciformes 2014.Piciformes
2015.Coraciiformes 2014.Coraciiformes
2015.Bucerotiformes 2014.Bucerotiformes
Nodes
2015 31
2014 14
congruent 66
Edges
is_a (inferred) 6
is_a (input) 120
overlaps 28
