# Supplementary material for: Verbalizing phylogenomic conflict: Representation of node congruence across competing reconstructions of the neoavian explosion
Source: PLoS Comput Biol. 2019 Feb 15;15(2):e1006493. doi: 10.1371/journal.pcbi.1006493 (PMC6395011; doi:10.1371/journal.pcbi.1006493)
Supplement: S16 File — (A) Reasoner input constraints for the 2015./2014.Neornithes alignment, under whole-concept resolution, ranging from the root to the ordinal level (with exceptions where needed). Includes information on run commands; and 4 instances of "no coverage". (B) Input visualization for the 2015./2014.Neornithes alignment, under whole-concept resolution, ranging from the root to the ordinal level (with exceptions where needed). (C) Alignment visualization for the 2015./2014.Neornithes alignment, under whole-concept resolution, ranging from the root to the ordinal level (with exceptions where needed). (D) Set of Maximally Informative Relations (MIR) inferred for the 2015./2014.Neornithes alignment, under whole-concept resolution, ranging from the root to the ordinal level (with exceptions where needed). Total = 8,051 MIR. (ZIP) [file S16B-Neornithes-Higher-Level-Overlap.pdf › S16B-Neornithes-Higher-Level-Overlap.pdf]

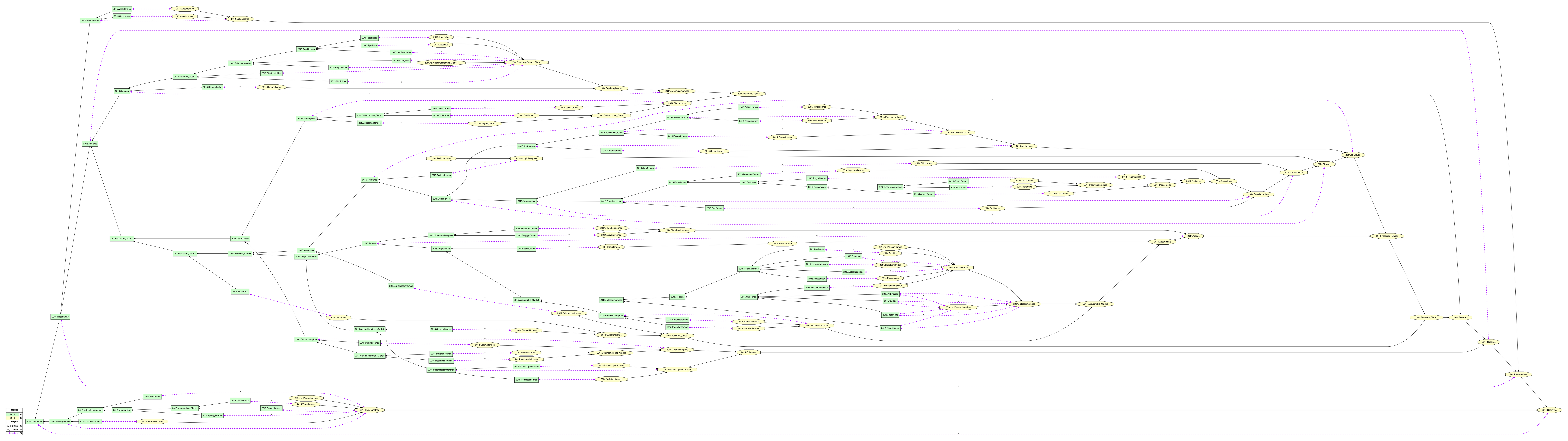
Nodes
2015 97
2014 83
Edges
is_a (2015) 96
is_a (2014) 82
articulations 74
2015.Neornithes
2015.Palaeognathae
2015.Notopalaeognathae
2015.Novaeratitae
2015.Struthioniformes
2015.Galloanserae
2015.Anseriformes
2015.Galliformes
2014.Galloanserae
2014.Palaeognathae
2014.Neornithes
2014.Neoaves
2015.Neoaves
2015.Neognathae
2014.Neognathae
