# Supplementary material for: Verbalizing phylogenomic conflict: Representation of node congruence across competing reconstructions of the neoavian explosion
Source: PLoS Comput Biol. 2019 Feb 15;15(2):e1006493. doi: 10.1371/journal.pcbi.1006493 (PMC6395011; doi:10.1371/journal.pcbi.1006493)
Supplement: S18 File — (A) Reasoner input constraints for the 2015./2014.Neoaves alignment, under split-concept resolution, limited to the main conflict region. Includes information on run commands; and 0 instances of "no coverage". (B) Input visualization for the 2015./2014.Neoaves alignment, under split-concept resolution, limited to the main conflict region. (C) Alignment visualization for the 2015./2014.Neoaves alignment, under split-concept resolution, limited to the main conflict region. (D) Set of Maximally Informative Relations (MIR) inferred for the 2015./2014.Neoaves alignment, under split-concept resolution, limited to the main conflict region. Total = 441 MIR. (ZIP) [file S18C-Neoaves-Conflict-Region-Overlap-Split-Concept-Resolution-0-mncb.pdf › S18C-Neoaves-Conflict-Region-Overlap-Split-Concept-Resolution-0-mncb.pdf]

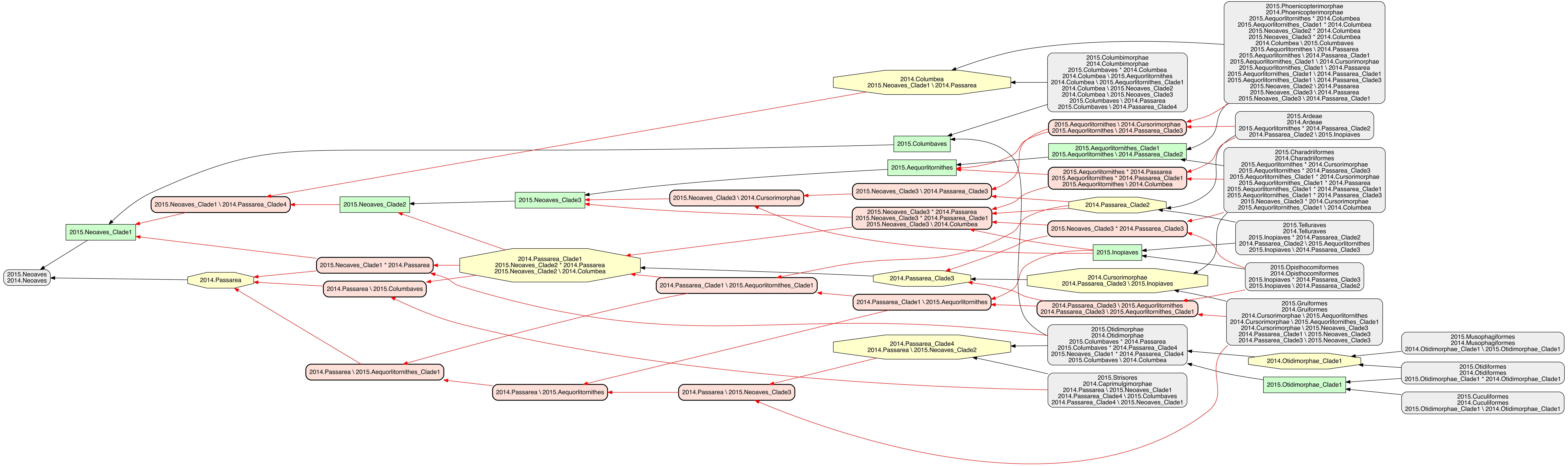

Nodes	
2015	8
2014	8
congruent	13
NEW	15
**Edges**	
is_a (input)	28
is_a (inferred)	46
